# Synthesis of 5-amino-*N*′-(9*H*-fluoren-9-ylidene)-8-nitro-7-aryl-1,2,3,7-tetrahydroimidazo[1,2-*a*]pyridine-6-carbohydrazide derivatives based on heterocyclic ketene aminals†

**DOI:** 10.1039/c8ra09308c

**Published:** 2018-12-11

**Authors:** Hajar Hosseini, Mohammad Bayat

**Affiliations:** Department of Chemistry, Faculty of Science, Imam Khomeini International University Qazvin Iran bayat_mo@yahoo.com m.bayat@sci.ikiu.ac.ir +98 (28) 33780040

## Abstract

A new class of tetrahydroimidazo[1,2-*a*]pyridine derivatives has been successfully prepared *via* a five-component domino reaction using cyanoacetohydrazide, 9-fluorenone, aromatic aldehydes, 1,1-bis(methylthio)-2-nitroethene and ethylenediamine in ethanol at reflux. The new efficient cascade approach involves a sequence of *N*,*N*-acetal formation, Knoevenagel condensation, Michael addition, imine–enamine tautomerization and *N*-cyclization as key steps. The merit of this protocol is highlighted by its available and economical starting compounds, operational simplicity, clean reaction profile and tolerance of a wide diversity of functional groups.

## Abstract 2

A new class of tetrahydroimidazo[1,2-*a*]pyridine derivatives has been successfully prepared *via* a five-component domino reaction using cyanoacetohydrazide, 9-fluorenone, aromatic aldehydes, 1,1-bis(methylthio)-2-nitroethene and ethylenediamine in ethanol at reflux.

## Graphical abstract

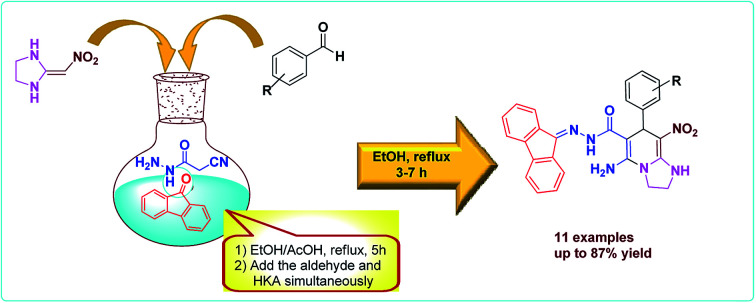

## Introduction

**Fig. 1:**
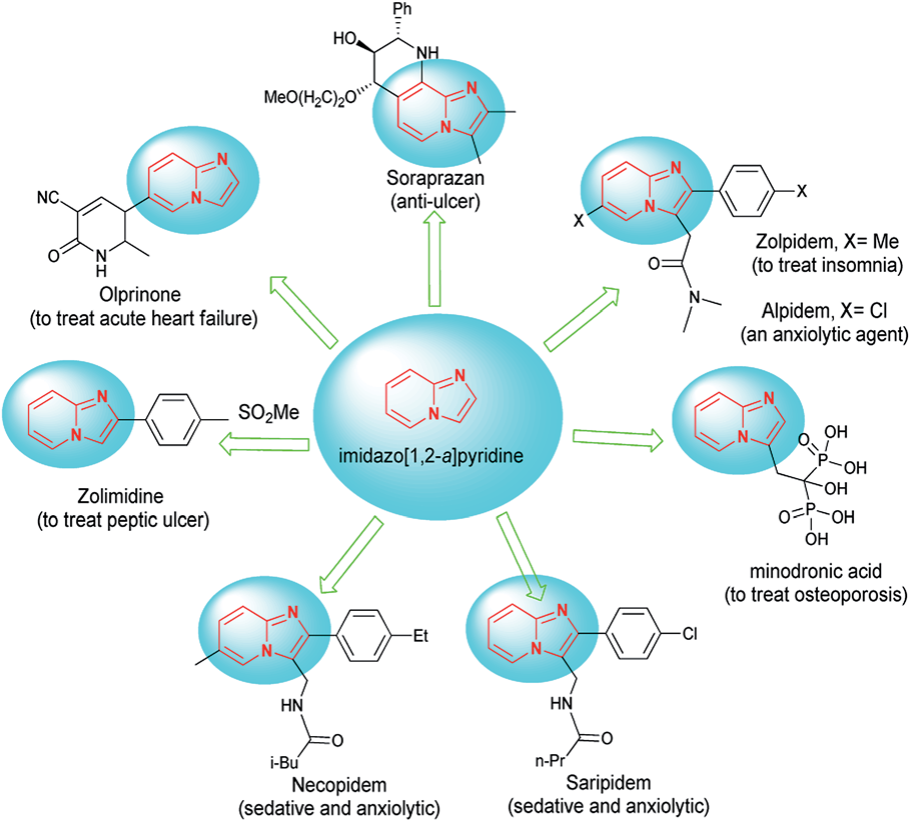
Drugs containing the imidazo[1,2-*a*]pyridine core.

Imidazopyridines have shown a broad spectrum of pharmacological and biological activities.^1^ Among the various derivatives, the imidazo[1,2-*a*]pyridine framework is likely the most important construction due to its vital role as a key structure in drugs and biologically active compounds with properties such as anti-inflammatory,^2,3^ antiviral,^4–6^ antiulcer agents,^7,8^ antifungal,^9^ anticancer,^10^ anxiolytic,^11^ anti-ulcer,^12^ and antiprotozoal.^13^ They are included in marketed drugs such as the clinical anti-ulcer compound zolpidem and alpidem,^14^ olprinone,^15^ zolimidine,^16^ necopidem and saripidem,^17^ soraprazan and minodronic acid^18^ (Fig. 1).

The design of reactions that minimize the number of synthetic steps for the rapid formation of functionalized molecules is one of the goals of modern synthesis. One way to achieve this purpose involves the development of multicomponent processes. Multicomponent reactions (MCRs) present a wide range of possibilities for the construction of complex molecules in a single step. The benefits of this approach include minimum time, labor and cost, high atom economy, and straight experimental procedures.^19^ These advantages are highlights for multicomponent cascade reactions, which contain *in situ* production of an intermediate with a reactive site for subsequent variations.^20^

By now, various synthetic methods have been developed to prepare imidazo[1,2-*a*]pyridines. The common strategies were the cyclocondensations of 2-aminopyridines with α-halocarbonyl compounds,^21^ 1,3-dicarbonyl compounds,^22^ nitroolefins or alkynes.^23^ Besides, the condensation of 2-aminopyridines, aldehydes and isonitriles or alkynes in a one-pot process was also an efficient method for the synthesis of imidazo[1,2-*a*]pyridines.^24^

There are still many efforts to the development of new methods for the synthesis of imidazo[1,2-*a*]pyridine derivatives with a variety of substituents at two rings. Some other novel synthetic approaches have been established in recent years for the synthesis of tetrahydroimidazo[1,2-*a*]pyridines by heterocyclic ketene aminals (HKAs).^25–29^ Heterocyclic ketene aminals (HKAs) have been proven to be efficient synthons in the synthesis of heterocyclic systems. During the past few years, reactions of cyclic ketene aminals with a variety of bis-electrophilic compounds have been applied to make five- and six-membered fused heterocycles.^30^

As a part of our current studies on synthesis of novel heterocyclic compounds using cyanoacetohydrazide, we describe herein an efficient one-pot five-component synthesis of novel imidazo[1,2-*a*]pyridine-6-carbohydrazides *via in situ* preparation of nitroketene aminal. These structures are completely new and there is no report on their synthesis.

## Results and discussion

We have developed an efficient synthesis of tetrahydroimidazo[1,2-*a*]pyridine-6-carbohydrazides *via* a one-pot five-component reaction. We used cyanoacetohydrazide 1, 9-fluorenone 2, aromatic aldehyde 3, 1,1-bis(methylthio)-2-nitroethene 4 and ethylenediamine 5 for the synthesis of title compounds.

### Optimization of the conditions

Initially, to identify the optimum reaction condition, 4-fluorobenzaldehyde was used as model substrate (since 4-fluorobenzaldehyde has clear reaction with obvious TLC at appropriate *R*_f_ value). At first, ethanol was used as solvent without any catalyst at reflux conditions and it was observed the desired product was not formed (Table 1, entry 1). The use of piperidine catalyst resulted in a yield of 40% in the product (entry 2). In order to improve yield, two other types of catalysts were used. With p-TSA, the five-component product did not form, and with acetic acid in a mixture of water and ethanol the efficiency did not change significantly (entry 3 and 5). The use of water and ethanol or water or acetonitrile without any catalyst resulted in no product formation (entry 4, 7 and 8). It was found that the reaction proceeded with high yield to formation of 5-amino-*N*′-(9*H*-fluoren-9-ylidene)-7-(4-fluorophenyl)-8-nitro-1,2,3,7-tetrahydroimidazo[1,2-*a*]pyridine-6-carbohydrazide 6a when ethanol was used as solvent and acetic acid was applied as catalyst at reflux conditions (entry 6).

**Table 1:** Optimization conditions for the formation of 6a^a^ using 4-fluorobenzaldehyde

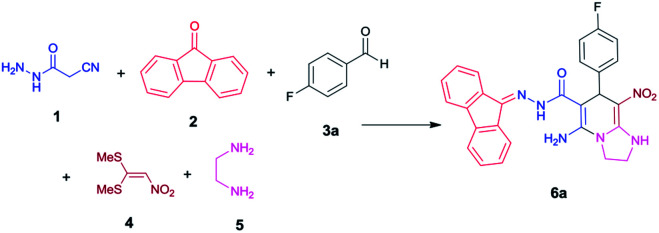					
Entry	Solvent	Catalyst (mol%)	Time (h)	Temp. (°C)	Yield (%)
1	EtOH	—	24	78	No reaction
2	EtOH	Piperidine	24	78	40
3	EtOH	p-TSA	24	78	No reaction
4	H_2_O/EtOH (1 : 1, v/v)	—	24	78	No reaction
5	H_2_O/EtOH (1 : 1, v/v)	AcOH	24	78	35
**6**	**EtOH**	**AcOH**	**9**	**78**	**87**
7	H_2_O	—	24	100	No reaction
8	CH_3_CN	—	24	82	No reaction

a Reagents and conditions: cyanoacetohydrazide (1 mmol), 9-fluorenone (1 mmol), 4-fluorobenzaldehyde (1 mmol), 1,1-bis(methylthio)-2-nitroethene (1 mmol), ethylenediamine (1 mmol), solvent (20 mL), catalyst (0.2 mmol).

It should be noted that initially a two-component reaction of cyanoacetohydrazide and 9-fluorenone is performed in the presence of acetic acid and then, without separating the product, aldehyde and ketene aminal are added.

**Scheme 1:**
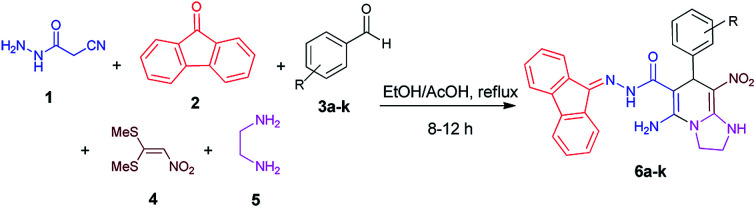
Synthetic scheme for the generation of products 6a–k.

With information obtained from optimization conditions table, we could synthesize target compounds 6a–k using cyanoacetohydrazide 1, 9-fluorenone 2, various aromatic aldehydes 3a–k, 1,1-bis(methylthio)-2-nitroethene 4 and ethylenediamine 5 as starting materials (Scheme 1).

The reactions were completed after 8–12 h overall to afford corresponding heterocyclic systems 6a–k in good to high yields (65–87%). The results are summarized in Table 2.

**Table 2:** Compounds 6a–k^a^

Entry	Aromatic aldehyde	Product	Time (h)	Yield (%)	Mp (°C)
1	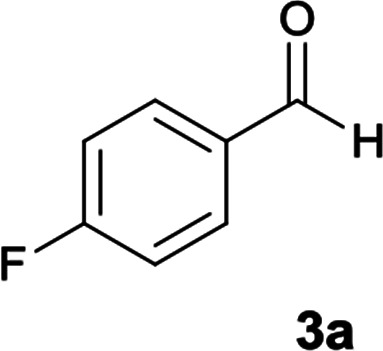	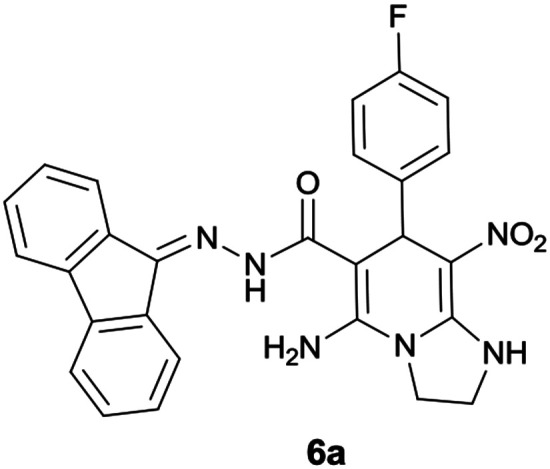	9	87	246–248
2	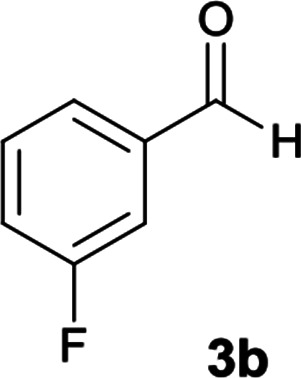	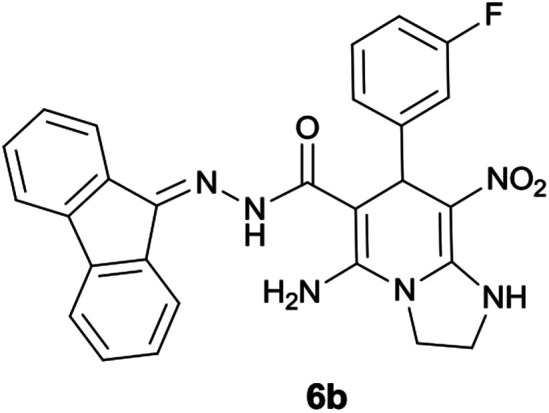	10	80	240–242
3	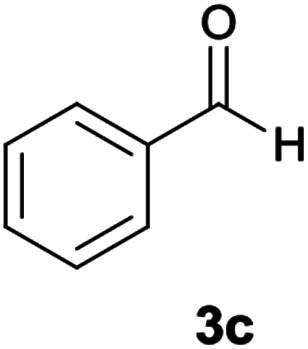	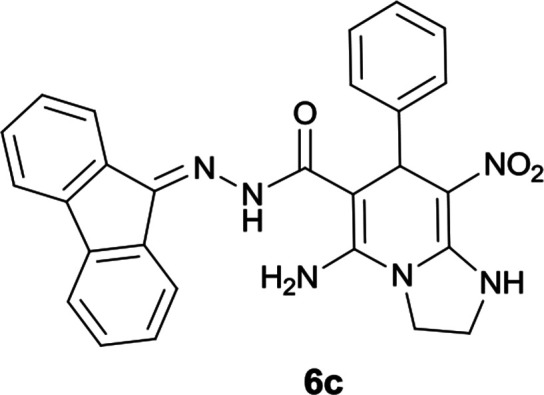	11	75	249–251
4	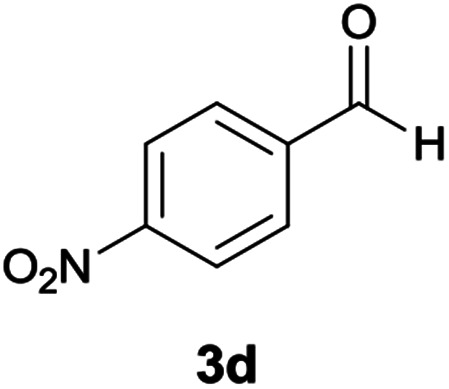	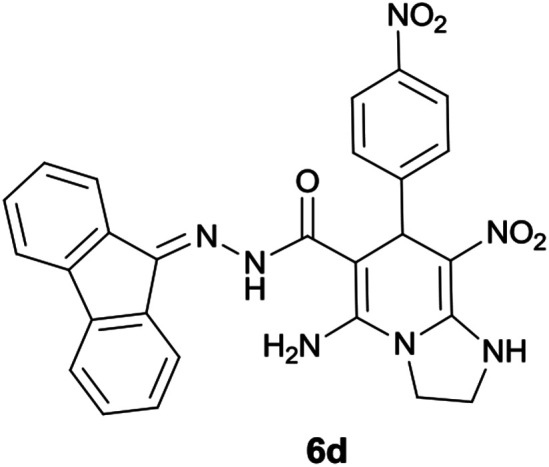	8	87	225–228
5	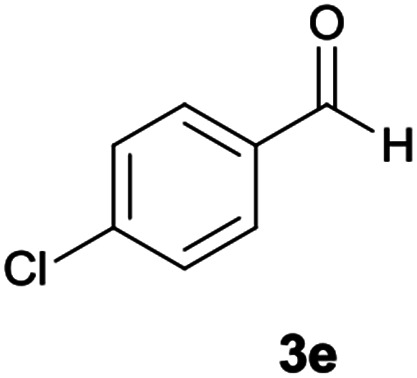	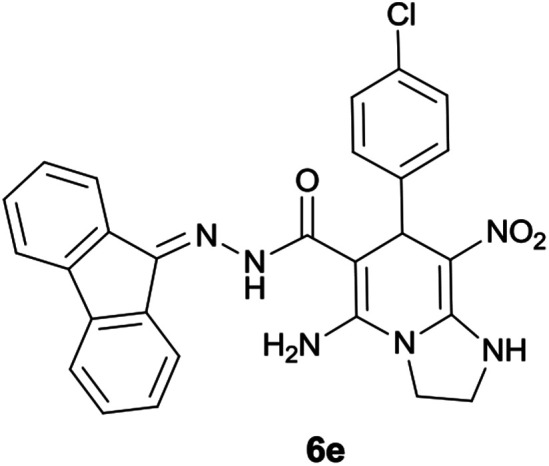	9	85	210–212
6	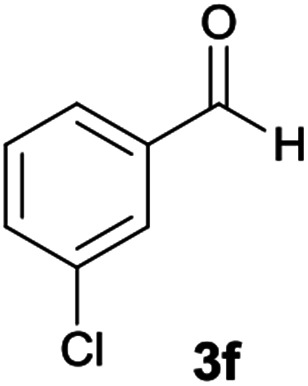	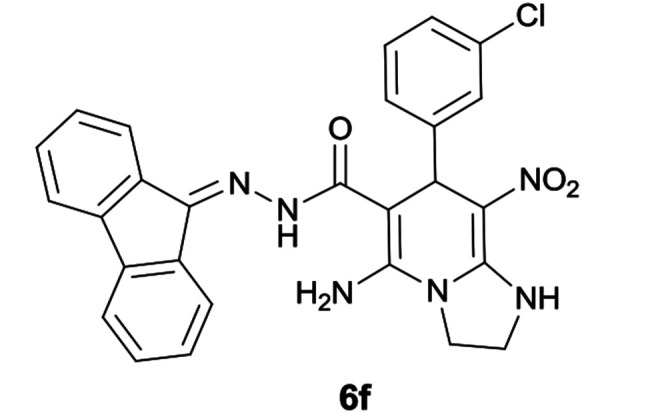	10	78	209–211
7	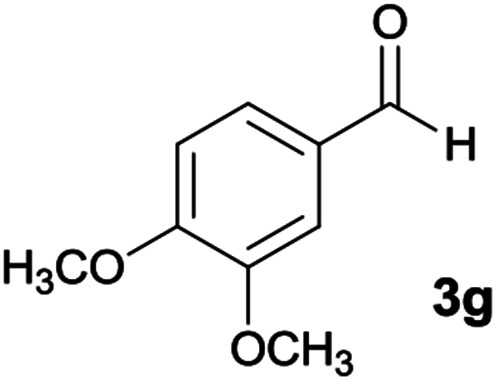	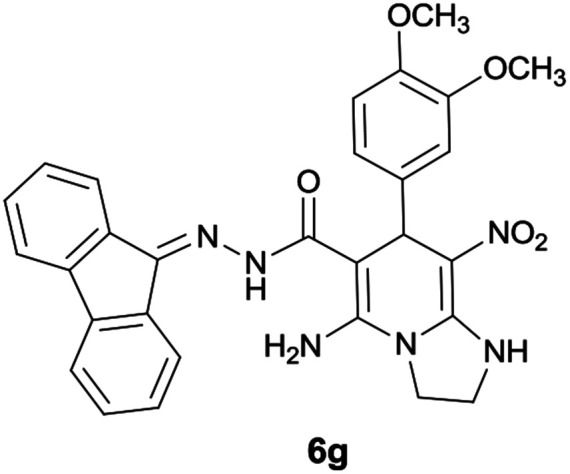	12	65	218–220
8	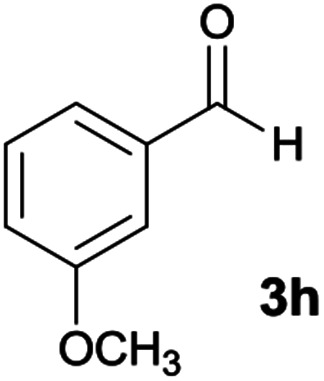	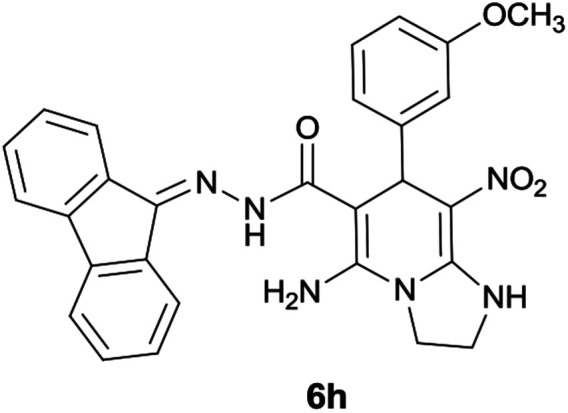	12	70	198–200
9	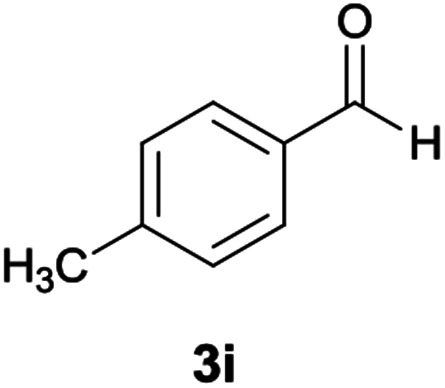	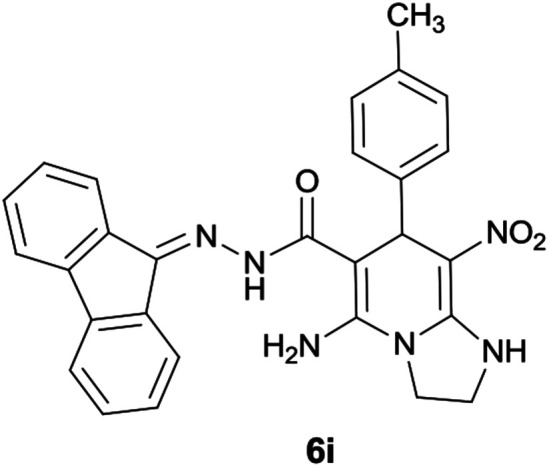	11	83	218–220
10	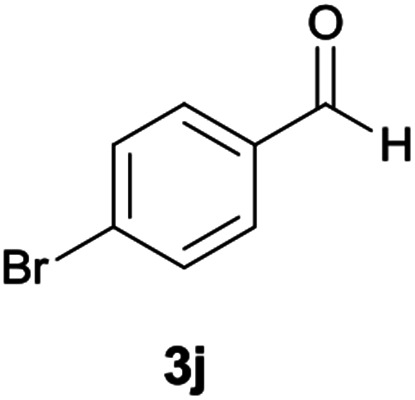	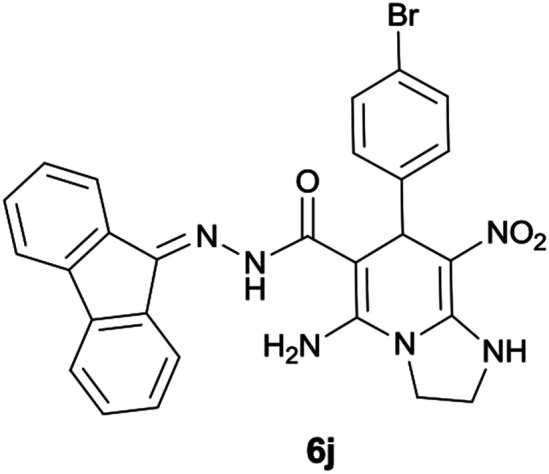	9	80	212–214
11	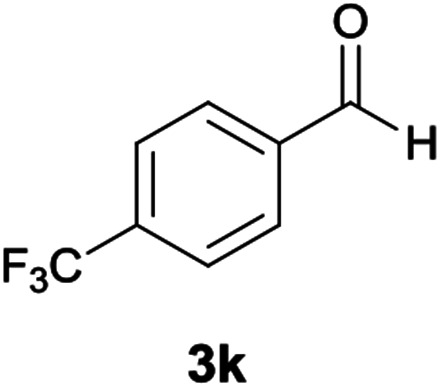	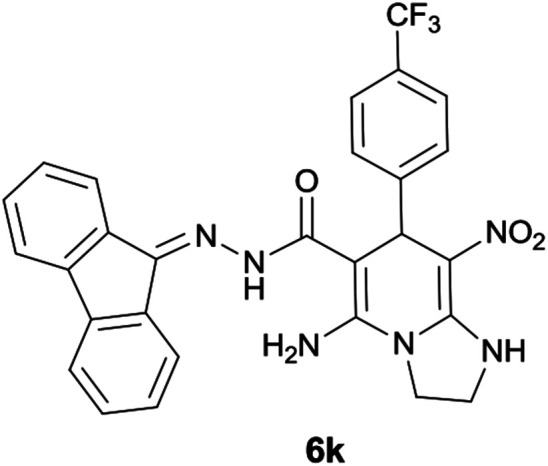	9	75	226–229

a The reaction was performed using cyanoacetohydrazide (1 mmol), 9-fluorenone (1 mmol), aromatic aldehyde (1 mmol), 1,1-bis(methylthio)-2-nitroethene (1 mmol), ethylenediamine (1 mmol), EtOH (20 mL), AcOH (1 mL), reflux.

### Effect of substituents

This reaction was performed with other derivatives of diamines (1,3-diaminopropane, 1,4-diaminobutane and 1,2-diaminocyclohexane) under the same conditions, which did not result in the desired product. Also the reaction with *ortho* derivatives of benzaldehyde (2-chloro and 2-nitro) did not produce the product, probably due to steric effects. For aldehydes with an electron-withdrawing group on *para* position of ring (nitro and halogens), the reaction rate is the highest and with methoxy group, the rate is the lowest.

It was found that the most important side product in these reactions was a four-component structure that was previously synthesized using two equivalents of aldehyde which will be further explained in the Mechanism section.

### Structure determination

The structures of compounds 6a–k were deduced from their IR, ^1^H NMR, ^13^C NMR spectroscopic and mass spectrometric data (see the ESI†).

**Fig. 2:**
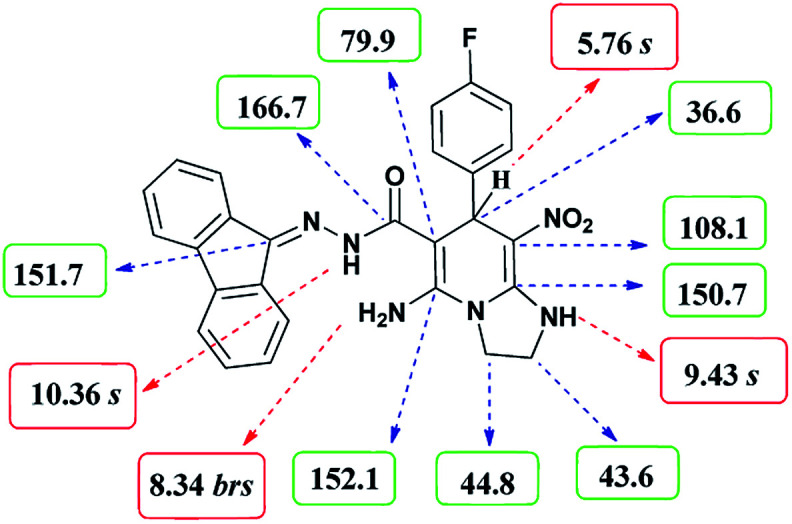
^1^H and ^13^C NMR chemical shifts of 6a.

The formation of proposed products 6a–k is clearly confirmed by the ^1^H and ^13^C NMR spectra of the crude products. As a representative case, the key signals of ^1^H and ^13^C NMR chemical shifts for 5-amino-*N*′-(9*H*-fluoren-9-ylidene)-7-(4-fluorophenyl)-8-nitro-1,2,3,7-tetrahydroimidazo[1,2-*a*]pyridine-6-carbohydrazide 6a are shown in Fig. 2.

The ^1^H NMR spectrum of 6a showed two NH groups at *δ* 9.43 and 10.36 ppm. The NH_2_ group appeared at *δ* 8.34 ppm. The protons of three aromatic rings were seen at *δ* 6.99–7.84 ppm. The proton of CH at pyridine ring was observed at *δ* 5.76 ppm. Two protons of two methylene groups appeared at *δ* 3.75–3.86 and 4.04–4.07 ppm.

The ^1^H-decoupled ^13^C NMR spectrum of 6a displayed 25 distinct signals in accordance to desired structure. The characteristic signals of three aliphatic carbons (CH and two CH_2_ groups) were observed at *δ* 36.6, 43.6 and 44.8 ppm respectively. Two signals at *δ* 79.9 and 108.1 ppm were related to C=*C*–CO and C–NO_2_ respectively. The carbonyl group appeared at *δ* 166.7 ppm (Fig. 2).

The mass spectrum of 6a displayed a molecular-ion peak at *m*/*z* 496 in agreement with the proposed product. The IR spectrum of this compound showed absorption bands at 3431, 3344, 3272 due to NH and NH_2_ groups, stretching vibration of aliphatic C–H bands at 2920, strong absorption of carbonyl group at 1654, stretching vibration of C=C of aromatic ring at 1445 and C–N stretching band at 1259 cm^−1^. Two absorption bands due to nitro group appeared at 1363 and 1528 cm^−1^.

### Mechanism

**Scheme 2:**
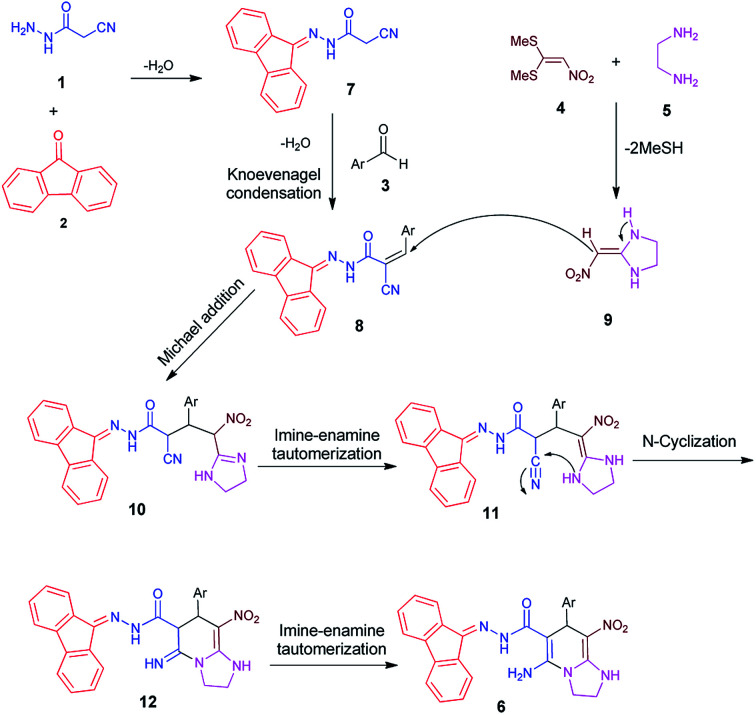
Proposed mechanism for the formation of products 6.

A typical plausible mechanism for the formation of 5-amino-*N*′-(9*H*-fluoren-9-ylidene)-7-aryl-8-nitro-1,2,3,7-tetrahydroimidazo[1,2-*a*]pyridine-6-carbohydrazide 6 is depicted in Scheme 2. On the basis of well-established chemistry of 1,1-bis(methylthio)-2-nitroethene, initially, addition of ethylenediamine 5 to 1,1-bis(methylthio)-2-nitroethene 4 leads to the formation of ketene aminal 9. On the other hand condensation of cyanoacetohydrazide 1 with 9-fluorenone 2 leads to hydrazone 7. Further, with increasing aldehyde 3, the Knoevenagel condensation affords intermediate 8. Then, Michael addition of ketene aminal 9 to adduct 8 leads to the intermediate 10, which undergoes successive imine–enamine tautomerization followed by an intramolecular cyclization *via* nucleophilic addition of –NH to nitrile group. Finally, another imine–enamine tautomerization gives the corresponding products 6 (Scheme 2). The most important side product in these reactions was a four-component structure without participation of 9-fluorenone.^25^ To prevent the formation of this product, firstly, the reaction of cyanoacetohydrazide with fluorenone was completed in the acidic medium within 5 hours, and then the aldehyde and the corresponding enamine are added simultaneously.

## Experimental

### Materials

All commercially available reagents and other solvents were purchased from Aldrich and Merck Chemical Co. and used without further purification. The NMR spectra were recorded with a Bruker DRX-300 AVANCE instrument (300 MHz for ^1^H and 75.4 MHz for ^13^C) with DMSO-*d*_6_ as solvent. Chemical shifts are given in ppm (*δ*) relative to internal TMS, and coupling constant (*J*) are reported in Hertz (Hz). Melting points were measured with an electrothermal 9100 apparatus. Mass spectra were recorded with an Agilent 5975C VL MSD with Triple-Axis detector operating at an ionization potential of 70 eV. IR spectra were measured with Bruker Tensor 27 spectrometer. Elemental analyses for C, H and N were performed using a PerkinElmer 2004 series [II] CHN elemental analyzer.

### General procedure of the synthesis of tetrahydroimidazo[1,2-*a*]pyridine-6-carbohydrazide derivatives

A mixture of ethylenediamine (66 mL, 1 mmol), 1,1-bis(methylthio)-2-nitroethylene (0.165 g, 1 mmol) and 10 mL EtOH in a 50 mL flask was refluxed for 6 hours. In another 50 mL flask the stoichiometric mixture of cyanoacetohydrazide (1 mmol, 0.99 g) and 9-fluorenone (1 mmol, 0.180 g) in EtOH (10 mL) and AcOH (1 mL) was stirred at reflux conditions for 5 hours. After this time, it is observed that precipitate is formed and TLC shows the consumption of the starting components. Then, aromatic aldehyde (1 mmol) and the first solution (HKA) was added to this mixture simultaneously. The progress of the reaction was monitored by TLC using ethyl acetate/*n*-hexane (1 : 1). After completion of the reaction, the precipitated product was collected by filtration and washed with warm ethanol to give the pure products 6a–k.

#### 5-Amino-*N*′-(9*H*-fluoren-9-ylidene)-7-(4-fluorophenyl)-8-nitro-1,2,3,7-tetrahydroimidazo[1,2-*a*]pyridine-6-carbohydrazide (6a)

Orange solid; yield: 0.431 g (87%); mp: 246–248 °C; IR (KBr) (*ν*_max_/cm^−1^): 3431, 3344, 3272, 2920, 1654, 1528, 1445, 1363, 1259, 1160; ^1^H NMR (300 MHz, DMSO): *δ* 3.75–3.86 (2H, m, CH_2_), 4.04–4.07 (2H, m, CH_2_), 5.76 (1H, s, CH), 6.99–7.45 (9H, m, ArH), 7.68 (1H, d, *J* = 7.2 Hz, ArH), 7.78 (1H, d, *J* = 7.5 Hz, ArH), 7.83 (1H, d, *J* = 7.5 Hz, ArH), 8.34 (2H, brs, NH_2_), 9.43 (1H, s, NH), 10.36 (1H, s, NH); ^13^C{^1^H} NMR (75.4 MHz, DMSO): *δ* 36.6 (CH), 43.6 (CH_2_–NH), 44.8 (CH_2_–N), 79.9 (C=**C**–CO), 108.1 (C=**C**–NO_2_), 115.0, 120.6, 120.9, 121.8, 126.9, 128.0, 128.5, 130.0, 130.1, 130.3, 131.1, 137.4, 139.5, 141.2, 141.8, 160.0 (Ar), 150.7 (C=**C**–NH), 151.7 (C=N), 152.1 (C=**C**–NH_2_), 166.7 (C=O); MS (EI, 70 eV): *m*/*z* (%) = 496 (0.07) [M]^+^, 453 (0.17), 407 (0.10), 356 (100), 327 (35), 276 (4), 254 (4), 230 (4), 194 (3), 178 (12), 164 (15), 150 (2), 133 (1), 97 (1), 69 (1); anal. calcd for C_27_H_21_FN_6_O_3_: C, 65.32; H, 4.26; N, 16.93. Found: C, 65.1; H, 4.2; N, 16.7.

#### 5-Amino-*N*′-(9*H*-fluoren-9-ylidene)-7-(3-fluorophenyl)-8-nitro-1,2,3,7-tetrahydroimidazo[1,2-*a*]pyridine-6-carbohydrazide (6b)

Pale orange solid; yield: 0.396 g (80%); mp: 240–242 °C; IR (KBr) (*ν*_max_/cm^−1^): 3352, 3267, 3152, 2918, 2856, 1650, 1468, 1333, 1252, 1159; ^1^H NMR (300 MHz, DMSO): *δ* 3.75–3.86 (2H, m, CH_2_), 4.04–4.10 (2H, m, CH_2_), 5.70 (1H, s, CH), 7.11–7.89 (12H, m, ArH), 8.37 (2H, brs, NH_2_), 9.40 (1H, s, NH), 10.33 (1H, s, NH); ^13^C{^1^H} NMR (75.4 MHz, DMSO): *δ* 37.0 (CH), 43.6 (CH_2_–NH), 44.8 (CH_2_–N), 79.5 (C=**C**–CO), 107.6 (C=**C**–NO_2_), 114.9, 120.6, 120.9, 121.8, 124.2, 127.0, 128.5, 130.1, 130.3, 131.1, 137.4, 139.5, 141.2, 148.5 (Ar), 150.8 (C=**C**–NH), 151.8 (C=N), 152.3 (C=**C**–NH_2_), 166.8 (C=O); MS (EI, 70 eV): *m*/*z* (%) = 496 (0.03) [M]^+^, 453 (1), 407 (0.4), 356 (100), 327 (40), 276 (40), 230 (56), 194 (79), 178 (24), 164 (90), 150 (9), 133 (9), 95 (7), 69 (8); anal. calcd for C_27_H_21_FN_6_O_3_: C, 65.32; H, 4.26; N, 16.93. Found: C, 65.7; H, 4.1; N, 16.8.

#### 5-Amino-*N*′-(9*H*-fluoren-9-ylidene)-8-nitro-7-phenyl-1,2,3,7-tetrahydroimidazo[1,2-*a*]pyridine-6-carbohydrazide (6c)

Yellow solid; yield: 0.358 g (75%); mp: 249–250 °C; IR (KBr) (*ν*_max_/cm^−1^): 3354, 3264, 3155, 2920, 1649, 1468, 1330, 1255; ^1^H NMR (300 MHz, DMSO): *δ* 3.72–3.90 (2H, m, CH_2_), 4.05–4.11 (2H, m, CH_2_), 5.68 (1H, s, CH), 7.16–7.52 (9H, m, ArH), 7.67 (1H, d, *J* = 7.2 Hz, ArH), 7.78 (1H, d, *J* = 7.5 Hz, ArH), 7.83 (1H, d, *J* = 7.2 Hz, ArH), 8.31 (2H, brs, NH_2_), 9.40 (1H, s, NH), 10.27 (1H, s, NH); ^13^C{^1^H} NMR (75.4 MHz, DMSO): *δ* 37.3 (CH), 43.6 (CH_2_–NH), 44.8 (CH_2_–N), 80.0 (C=**C**–CO), 108.2 (C=**C**–NO_2_), 120.6, 120.9, 121.7, 126.9, 127.1, 128.2, 128.5, 128.7, 129.1, 130.0, 130.2, 131.0, 137.4, 139.4, 141.1, 145.5 (Ar), 149.9 (C=**C**–NH), 151.8 (C=N), 152.2 (C=**C**–NH_2_), 166.7 (C=O); MS (EI, 70 eV): *m*/*z* (%) = 478 (0.02) [M]^+^, 435 (2), 419 (0.9), 356 (78), 327 (35), 258 (33), 212 (54), 194 (67), 178 (22), 164 (100), 150 (8), 115 (8), 96 (0.9), 77 (3); anal. calcd for C_27_H_22_N_6_O_3_: C, 67.77; H, 4.63; N, 17.56. Found: C, 67.4; H, 4.9; N, 17.3.

#### 5-Amino-*N*′-(9*H*-fluoren-9-ylidene)-8-nitro-7-(4-nitrophenyl)-1,2,3,7-tetrahydroimidazo[1,2-*a*]pyridine-6-carbohydrazide (6d)

Orange solid; yield: 0.455 g (87%); mp: 225–228 °C; ^1^H NMR (300 MHz, DMSO): *δ* 3.78–3.88 (2H, m, CH_2_), 4.06–4.09 (2H, m, CH_2_), 5.87 (1H, s, CH), 7.02–7.82 (12H, m, ArH), 8.33 (2H, brs, NH_2_), 9.49 (1H, s, NH), 10.50 (1H, s, NH); ^13^C{^1^H} NMR (75.4 MHz, DMSO): *δ* 37.6 (CH), 43.7 (CH_2_–NH), 44.8 (CH_2_–N), 79.4 (C=**C**–CO), 107.0 (C=**C**–NO_2_), 120.6, 120.9, 121.8, 123.7, 127.0, 128.6, 129.6, 130.2, 130.4, 131.1, 137.4, 139.6, 141.3, 146.5 (Ar), 151.7 (C=**C**–NH), 151.8 (C=N), 152.1 (C=**C**–NH_2_), 153.4 (C_Ar_–NO_2_), 166.8 (C=O); anal. calcd for C_27_H_21_N_7_O_5_: C, 61.95; H, 4.04; N, 18.73. Found: C, 61.6; H, 4.3; N, 18.5.

#### 5-Amino-7-(4-chlorophenyl)-*N′*-(9*H*-fluoren-9-ylidene)-8-nitro-1,2,3,7-tetrahydroimidazo[1,2-*a*]pyridine-6-carbohydrazide (6e)

Light red solid; yield: 0.435 g (85%); mp: 210–212 °C; ^1^H NMR (300 MHz, DMSO): *δ* 3.75–3.83 (2H, m, CH_2_), 4.04–4.10 (2H, m, CH_2_), 5.70 (1H, s, CH), 7.10–7.50 (9H, m, ArH), 7.68 (1H, d, *J* = 7.2 Hz, ArH), 7.78 (1H, d, *J* = 7.2 Hz, ArH), 7.83 (1H, d, *J* = 7.5 Hz, ArH), 8.30 (2H, brs, NH_2_), 9.41 (1H, s, NH), 10.32 (1H, s, NH); ^13^C{^1^H} NMR (75.4 MHz, DMSO): *δ* 36.8 (CH), 43.6 (CH_2_–NH), 44.8 (CH_2_–N), 79.8 (C=**C**–CO), 107.7 (C=**C**–NO_2_), 120.6, 120.9, 121.8, 126.9, 127.9, 128.4, 128.5, 129.2, 130.1, 130.3, 131.1, 131.5, 137.4, 139.5, 141.2, 144.6 (Ar), 150.9 (C=C–NH), 151.7 (C=N), 152.1 (C=**C**–NH_2_), 166.7 (C=O); MS (EI, 70 eV): *m*/*z* (%) = 356 (9), 327 (5), 292 (21), 275 (19), 246 (41), 220 (35), 194 (15), 164 (100), 135 (6), 95 (5), 69 (9), 44 (21); anal. calcd for C_27_H_21_ClN_6_O_3_: C, 63.22; H, 4.13; N, 16.38. Found: C, 63.6; H, 4; 5 N, 16.1.

#### 5-Amino-7-(3-chlorophenyl)-*N*′-(9*H*-fluoren-9-ylidene)-8-nitro-1,2,3,7-tetrahydroimidazo[1,2-*a*]pyridine-6-carbohydrazide (6f)

Light orange solid; yield: 0.399 g (78%); mp: 209–211 °C; IR (KBr) (*ν*_max_/cm^−1^): 3414, 3351, 3169, 2919, 2855, 1653, 1442, 1352, 1254, 774; ^1^H NMR (300 MHz, DMSO): *δ* 3.76–3.86 (2H, m, CH_2_), 4.04–4.09 (2H, m, CH_2_), 5.74 (1H, s, CH), 7.12–7.53 (9H, m, ArH), 7.68 (1H, d, *J* = 7.2 Hz, ArH), 7.78 (1H, d, *J* = 7.5 Hz, ArH), 7.83 (1H, d, *J* = 7.5 Hz, ArH), 8.34 (2H, brs, NH_2_), 9.44 (1H, s, NH), 10.38 (1H, s, NH); ^13^C{^1^H} NMR (75.4 MHz, DMSO): *δ* 37.1 (CH), 43.6 (CH_2_–NH), 44.8 (CH_2_–N), 79.4 (C=C–CO), 107.5 (C=C–NO_2_), 120.6, 120.9, 121.8, 126.8, 127.0, 128.1, 128.2, 128.5, 130.1, 130.3, 130.5, 131.0, 133.0, 137.4, 139.5, 141.2, 148.1 (Ar), 150.9 (C=C–NH), 151.7 (C=N), 152.3 (C=C–NH_2_), 166.8 (C=O); anal. calcd for C_27_H_21_ClN_6_O_3_: C, 63.22; H, 4.13; N, 16.38. Found: C, 63.4; H, 4.4; N, 16.3.

#### 5-Amino-7-(3,4-dimethoxyphenyl)-*N*′-(9*H*-fluoren-9-ylidene)-8-nitro-1,2,3,7-tetrahydroimidazo[1,2-*a*]pyridine-6-carbohydrazide (6g)

Orange solid; yield: 0.349 g (65%); mp: 218–221 °C; ^1^H NMR (300 MHz, DMSO): *δ* 3.61 (3H, s, OCH_3_), 3.66 (3H, s, OCH_3_), 3.74–3.89 (2H, m, CH_2_), 4.04–4.10 (2H, m, CH_2_), 5.63 (1H, s, CH), 6.73–7.48 (8H, m, ArH), 7.68 (1H, d, *J* = 7.5 Hz, ArH), 7.78 (1H, d, *J* = 7.2 Hz, ArH), 7.83 (1H, d, *J* = 7.8 Hz, ArH), 8.34 (2H, brs, NH_2_), 9.38 (1H, s, NH), 10.12 (1H, s, NH); ^13^C{^1^H} NMR (75.4 MHz, DMSO): *δ* 36.7 (CH), 43.6 (CH_2_–NH), 44.8 (CH_2_–N), 55.7 (OCH_3_), 56.0 (OCH_3_), 79.8 (C=C–CO), 108.3 (C=C–NO_2_), 112.3, 112.7, 119.7, 120.6, 121.0, 121.7, 126.7, 127.9, 128.5, 129.3, 130.1, 131.1, 137.4, 138.0, 139.4, 141.2, 148.1, 148.7 (Ar), 149.3 (C=C–NH), 151.8 (C=N), 152.3 (C=C–NH_2_), 166.6 (C=O); anal. calcd for C_29_H_26_N_6_O_5_: C, 64.68; H, 4.87; N, 15.60. Found: C, 64.3; H, 4.6; N, 15.7.

#### 5-Amino-*N*′-(9*H*-fluoren-9-ylidene)-7-(3-methoxyphenyl)-8-nitro-1,2,3,7-tetrahydroimidazo[1,2-*a*]pyridine-6-carbohydrazide (6h)

Light orange solid; yield: 0.355 g (70%); mp: 198–200 °C; IR (KBr) (*ν*_max_/cm^−1^): 3358, 3262, 3158, 2921, 2859, 1649, 1452, 1332, 1254, 1159; ^1^H NMR (300 MHz, DMSO): *δ* 3.65 (3H, s, OCH_3_), 3.74–3.84 (2H, m, CH_2_), 4.05–4.09 (2H, m, CH_2_), 5.67 (1H, s, CH), 6.75–7.46 (9H, m, ArH), 7.67 (1H, d, *J* = 7.2 Hz, ArH), 7.78 (1H, d, *J* = 7.2 Hz, ArH), 7.83 (1H, d, *J* = 7.5 Hz, ArH), 8.32 (2H, brs, NH_2_), 9.39 (1H, s, NH), 10.25 (1H, s, NH); ^13^C{^1^H} NMR (75.4 MHz, DMSO): *δ* 37.2 (CH), 43.6 (CH_2_–NH), 44.8 (CH_2_–N), 55.2 (OCH_3_), 79.8 (C=C–CO), 108.0 (C=C–NO_2_), 111.6, 114.8, 120.2, 120.6, 120.9, 121.7, 126.9, 128.1, 128.5, 129.7, 130.0, 130.2, 131.0, 137.4, 139.4, 141.1, 147.1 (Ar), 150.8 (C=C–NH), 151.8 (C=N), 152.3 (C=C–NH_2_), 159.5 (C_Ar_–OMe), 166.7 (C=O); MS (EI, 70 eV): *m*/*z* (%) = 508 (0.06) [M]^+^, 435 (0.1), 356 (4), 327 (1), 292 (5), 276 (4), 220 (7), 194 (6), 163 (100), 134 (55), 105 (14), 85 (7), 57 (12); anal. calcd for C_28_H_24_N_6_O_4_: C, 66.13; H, 4.76; N, 16.53. Found: C, 66.3; H, 4.5; N, 16.3.

#### 5-Amino-*N*′-(9*H*-fluoren-9-ylidene)-8-nitro-7-(*p*-tolyl)-1,2,3,7-tetrahydroimidazo[1,2-*a*]pyridine-6-carbohydrazide (6i)

Light brown solid; yield: 0.408 g (83%); mp: 218–220 °C; IR (KBr) (*ν*_max_/cm^−1^): 3349, 2917, 1661, 1607, 1511, 1448, 1352, 1261, 1123; ^1^H NMR (300 MHz, DMSO): *δ* 2.20 (3H, s, CH_3_), 3.74–3.86 (2H, m, CH_2_), 4.04–4.10 (2H, m, CH_2_), 5.61 (1H, s, CH), 7.08–7.47 (9H, m, ArH), 7.67 (1H, d, *J* = 7.5 Hz, ArH), 7.78 (1H, d, *J* = 7.5 Hz, ArH), 7.83 (1H, d, *J* = 7.8 Hz, ArH), 8.29 (2H, brs, NH_2_), 9.37 (1H, s, NH), 10.21 (1H, s, NH); ^13^C{^1^H} NMR (75.4 MHz, DMSO): *δ* 21.1 (CH_3_), 36.9 (CH), 43.5 (CH_2_–NH), 44.8 (CH_2_–N), 80.1 (C=C–CO), 108.3 (C=C–NO_2_), 120.6, 120.9, 121.7, 126.9, 128.1, 128.5, 129.0, 130.0, 130.1, 131.0, 136.1, 137.4, 139.4, 141.1, 142.5 (Ar), 149.6 (C=C–NH), 151.7 (C=N), 152.2 (C=C–NH_2_), 166.6 (C=O); MS (EI, 70 eV): *m*/*z* (%) = 492 (0.04) [M]^+^, 435 (0.08), 356 (100), 327 (39), 272 (20), 226 (30), 194 (50), 165 (59), 115 (7), 69 (8); anal. calcd for C_28_H_24_N_6_O_3_: C, 68.28; H, 4.91; N, 17.06. Found: C, 68.7; H, 4.6; N, 17.2.

#### 5-Amino-7-(4-bromophenyl)-*N*′-(9*H*-fluoren-9-ylidene)-8-nitro-1,2,3,7-tetrahydroimidazo[1,2-*a*]pyridine-6-carbohydrazide (6j)

Yellow solid; yield: 0.444 g (80%); mp: 212–214 °C; ^1^H NMR (300 MHz, DMSO): *δ* 3.75–3.83 (2H, m, CH_2_), 4.03–4.06 (2H, m, CH_2_), 5.69 (1H, s, CH), 7.05–7.84 (12H, m, ArH), 8.29 (2H, brs, NH_2_), 9.42 (1H, s, NH), 10.33 (1H, s, NH); ^13^C{^1^H} NMR (75.4 MHz, DMSO): *δ* 36.9 (CH), 43.6 (CH_2_–NH), 44.8 (CH_2_–N), 79.7 (C=C–CO), 107.7 (C=C–NO_2_), 120.0, 120.6, 120.9, 121.8, 126.9, 127.9, 128.5, 130.1, 130.3, 130.5, 131.1, 131.3, 137.4, 139.6, 141.2, 145.0 (Ar), 151.0 (C=C–NH), 151.7 (C=N), 152.1 (C=C–NH_2_), 166.7 (C=O); anal. calcd for C_27_H_21_BrN_6_O_3_: C, 58.18; H, 3.80; N, 15.08. Found: C, 58.4; H, 3.5; N, 15.3.

#### 5-Amino-*N*′-(9*H*-fluoren-9-ylidene)-8-nitro-7-(4-(trifluoromethyl)phenyl)-1,2,3,7-tetrahydroimidazo[1,2-*a*]pyridine-6-carbohydrazide (6k)

Dark yellow solid; yield: 0.409 g (75%); mp: 226–229 °C; ^1^H NMR (300 MHz, DMSO): *δ* 3.76–3.87 (2H, m, CH_2_), 4.05–4.11 (2H, m, CH_2_), 5.81 (1H, s, CH), 6.99–7.85 (12H, m, ArH), 8.31 (2H, brs, NH_2_), 9.46 (1H, s, NH), 10.44 (1H, s, NH); ^13^C{^1^H} NMR (75.4 MHz, DMSO): *δ* 36.6 (CH), 43.6 (CH_2_–NH), 44.8 (CH_2_–N), 79.9 (C=C–CO), 108.0 (C=C–NO_2_), 115.0, 115.2, 120.6, 120.9, 120.9, 121.8, 126.9, 128.5, 130.0, 130.1, 130.3, 131.0, 137.4, 139.5, 141.2, 141.8 (Ar), 150.7 (C=C–NH), 151.7 (C=N), 152.1 (C=C–NH_2_), 166.7 (C=O); anal. calcd for C_28_H_21_FN_6_O_3_: C, 61.54; H, 3.87; N, 15.38. Found: C, 61.8; H, 3.7; N, 15.2.

## Conclusion

A green and highly efficient method for the synthesis of imidazo[1,2-*a*]pyridine core has been created by annulation of heterocyclic ketene aminals (HKAs) and a three-component product of cyanoacetohydrazide, 9-fluorenone and aromatic aldehydes that is produced under reaction conditions and can not be separated. The reactions are completed within 8–12 h in EtOH in the presence of acetic acid at reflux conditions. This methodology provides a novel approach for easy construction of highly substituted imidazopyridines in good yields. The present synthesis shows significant properties such as high regioselectivity, cascade one-pot reaction, relatively short reaction times, simple purification of products and high atom economy.

## Conflicts of interest

The authors declare no competing financial interest.

## Supplementary Material

RA-008-C8RA09308C-s001
