# Supplementary material for: Synthesis of 5-amino-N′-(9H-fluoren-9-ylidene)-8-nitro-7-aryl-1,2,3,7-tetrahydroimidazo[1,2-a]pyridine-6-carbohydrazide derivatives based on heterocyclic ketene aminals
Source: RSC Adv. 2018 Dec 11;8(72):41218–25. doi: 10.1039/c8ra09308c (PMC9091710; doi:10.1039/c8ra09308c)
Supplement: RA-008-C8RA09308C-s001 [file RA-008-C8RA09308C-s001.pdf]

Electronic Supplementary Material (ESI) for RSC Advances.
This journal is © The Royal Society of Chemistry 2018

Supporting Information

Synthesis of 5-amino-*N'*-(9*H*-fluoren-9-ylidene)-8-nitro-7-aryl-1,2,3,7-tetrahydroimidazo[1,2-*a*]pyridine-6-carbohydrazide derivatives based on heterocyclic ketene aminals

Hajar Hosseini, Mohammad Bayat

The Table of Contents

Title	Page
Title, author's name, address and table of contents	1-2
Experimental Section; General remarks	2
**Figure 1.** Structure of all products **6a-k**	3
^{1}H and ^{13}C NMR and IR and Mass spectrums of **6a**	4-7
^{1}H and ^{13}C NMR and IR and Mass spectrums of **6b**	8-11
^{1}H and ^{13}C NMR and IR and Mass spectrums of **6c**	12-15
^{1}H and ^{13}C NMR spectrums of **6d**	16-17
^{1}H and ^{13}C NMR and Mass spectrums of **6e**	18-20
^{1}H and ^{13}C NMR and IR and Mass spectrums of **6f**	21-24
^{1}H and D_2O exchange and ^{13}C NMR of **6g**	25-26
^{1}H and ^{13}C NMR and IR and Mass spectrums of **6h**	27-30
^{1}H and D_2O exchange and ^{13}C NMR and IR and Mass spectrums of **6i**	31-34
^{1}H and ^{13}C NMR spectrums of **6j**	35-36
^{1}H and ^{13}C NMR spectrums of **6k**	37-38

Experimental Section

General remarks:

Melting points were measured on an Electrothermal 9100 apparatus. Mass spectra were recorded with an Agilent 5975C VL MSD with Triple-Axis Detector operating at an ionization potential of 70 eV. ^1H and ^{13}C NMR spectra were measured (DMSO) with a Bruker DRX-300 AVANCE spectrometer at 300 and 75 MHz, respectively. IR spectra were recorded on a Bruker Tensor 27, ῡ in cm^{-1}. All NMR spectra at room temperature were determined in DMSO-d_6. Chemical shifts are reported in parts per million (δ) downfield from an internal tetramethylsilane reference. Coupling constants (J values) are reported in hertz (Hz), and spin multiplicities are indicated by the following symbols: s (singlet), d (doublet), t (triplet), q (quartet), m (multiplet). All chemicals were purchased from Merck or Aldrich and were used without further purification.

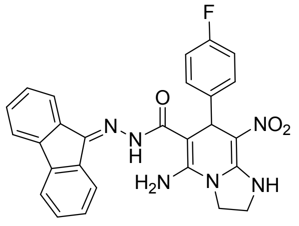

6a

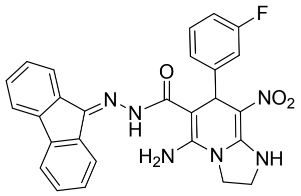

6b

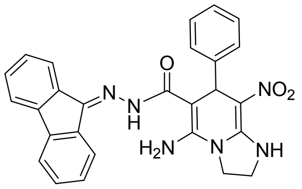

6c

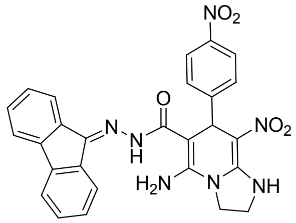

6d

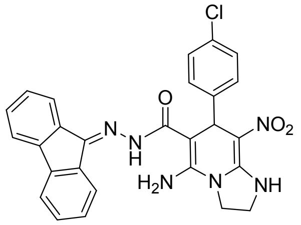

6e

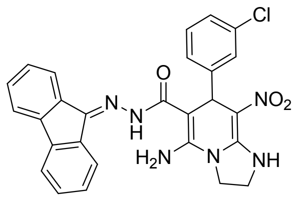

6f

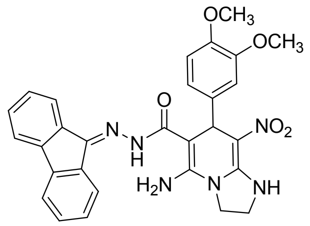

6g

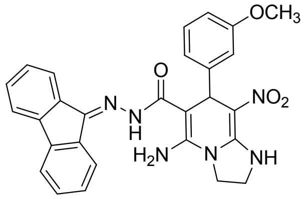

6h

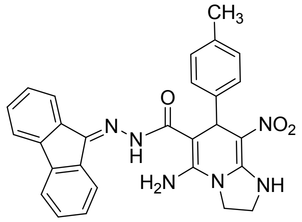

6i

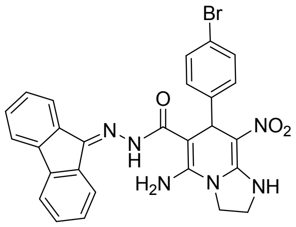

6j

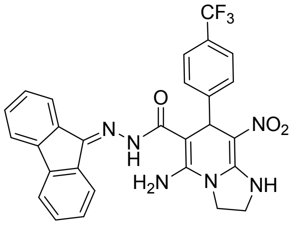

6k

Figure 1. Structure of all products **6a-k.**

The structures of all products **6a-k** were deduced from their IR, mass, ^{1}H NMR, and ^{13}C NMR spectra (see the following images).

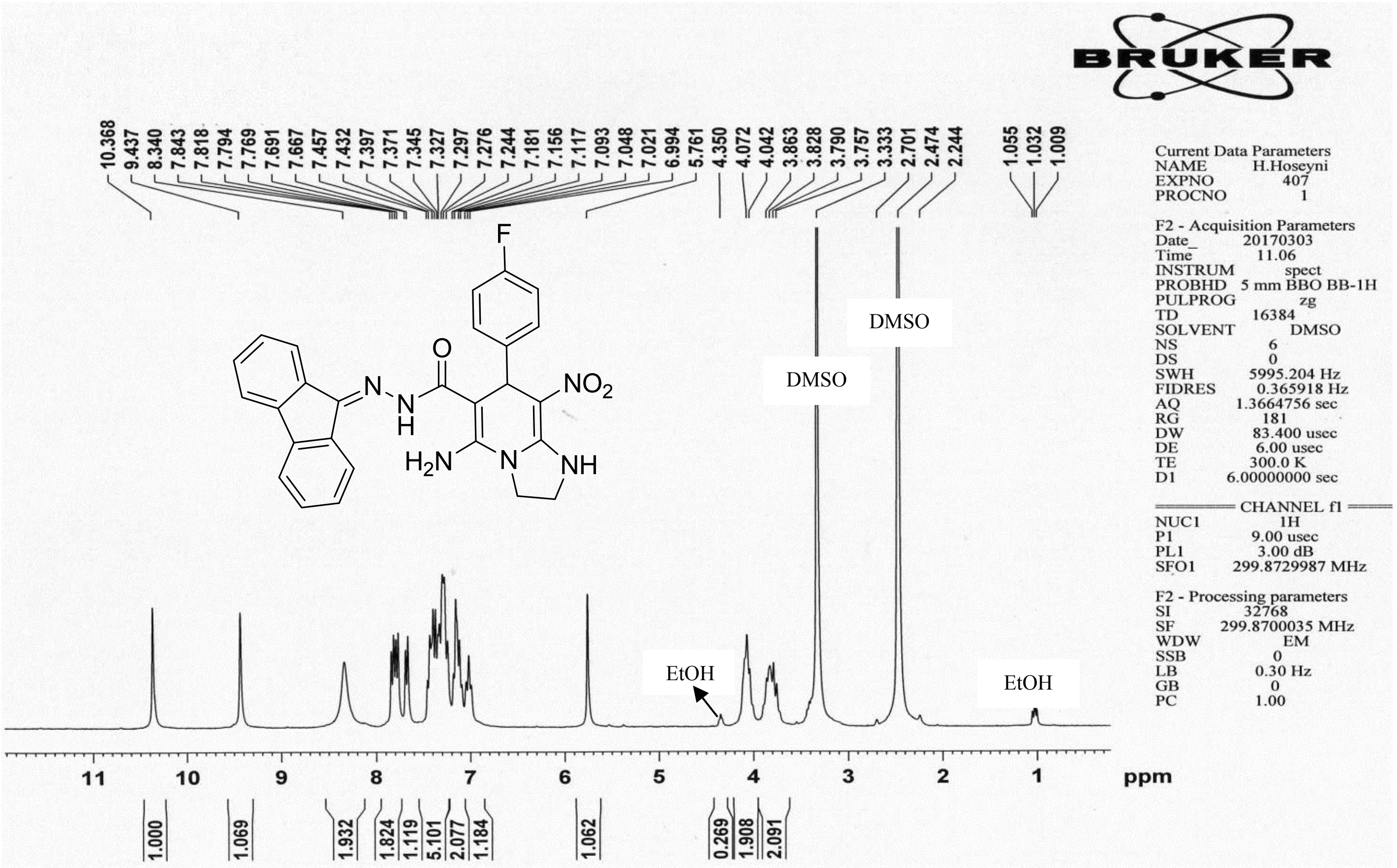
BRUKER
10.368
9.437
8.340
7.843
7.818
7.794
7.769
7.691
7.667
7.457
7.432
7.397
7.371
7.345
7.327
7.297
7.276
7.244
7.181
7.156
7.117
7.093
7.048
7.021
6.994
5.761
4.350
4.072
4.042
3.863
3.828
3.790
3.757
3.333
2.701
2.474
2.244
1.055
1.032
1.009
F
O
N
N
H
NO2
H2N
N
NH
DMSO
DMSO
EtOH
EtOH
Current Data Parameters
NAME H.Hoseyni
EXPNO 407
PROCNO 1
F2 - Acquisition Parameters
Date_ 20170303
Time 11.06
INSTRUM spect
PROBHD 5 mm BBO BB-1H
PULPROG zg
TD 16384
SOLVENT DMSO
NS 6
DS 0
SWH 5995.204 Hz
FIDRES 0.365918 Hz
AQ 1.3664756 sec
RG 181
DW 83.400 usec
DE 6.00 usec
TE 300.0 K
D1 6.00000000 sec
CHANNEL f1
NUC1 1H
P1 9.00 usec
PL1 3.00 dB
SFO1 299.8729987 MHz
F2 - Processing parameters
SI 32768
SF 299.8700035 MHz
WDW EM
SSB 0
LB 0.30 Hz
GB 0
PC 1.00
11
10
9
8
7
6
5
4
3
2
1
ppm
1.000
1.069
1.932
1.824
1.119
5.101
2.077
1.184
1.062
0.269
1.908
2.091

^{1}H NMR of 6a

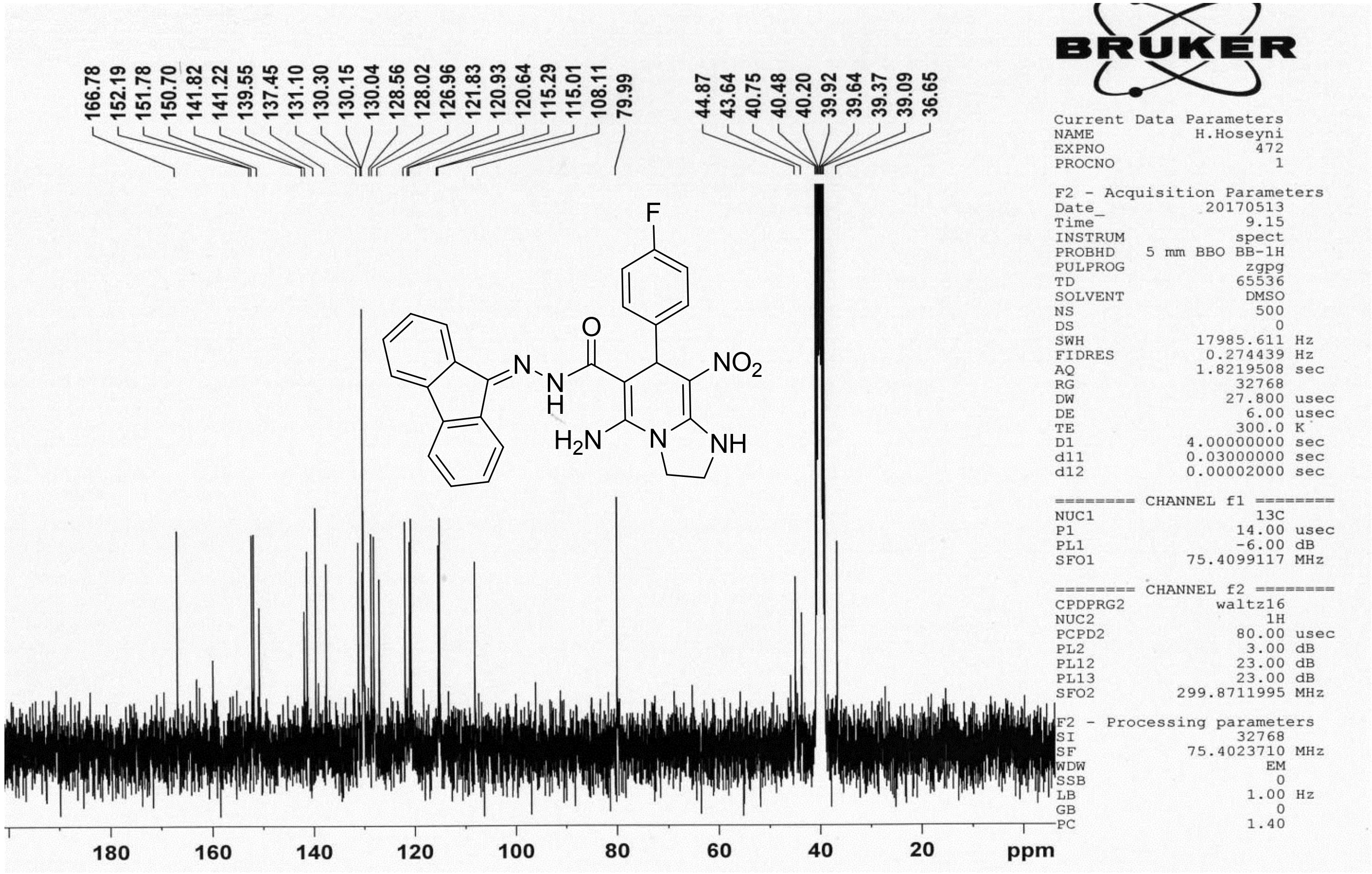
166.78
152.19
151.78
150.70
141.82
141.22
139.55
137.45
131.10
130.30
130.15
130.04
128.56
128.02
126.96
121.83
120.93
120.64
115.29
115.01
108.11
79.99
44.87
43.64
40.75
40.48
40.20
39.92
39.64
39.37
39.09
36.65
BRUKER
Current Data Parameters
NAME H.Hoseyni
EXPNO 472
PROCNO 1
F2 - Acquisition Parameters
Date_ 20170513
Time 9.15
INSTRUM spect
PROBHD 5 mm BBO BB-1H
PULPROG zgpg
TD 65536
SOLVENT DMSO
NS 500
DS 0
SWH 17985.611 Hz
FIDRES 0.274439 Hz
AQ 1.8219508 sec
RG 32768
DW 27.800 usec
DE 6.00 usec
TE 300.0 K
D1 4.00000000 sec
d11 0.03000000 sec
d12 0.00002000 sec
======== CHANNEL f1 ========
NUC1 13C
P1 14.00 usec
PL1 -6.00 dB
SFO1 75.4099117 MHz
======== CHANNEL f2 ========
CPDPRG2 waltz16
NUC2 1H
PCPD2 80.00 usec
PL2 3.00 dB
PL12 23.00 dB
PL13 23.00 dB
SFO2 299.8711995 MHz
F2 - Processing parameters
SI 32768
SF 75.4023710 MHz
WDW EM
SSB 0
LB 1.00 Hz
GB 0
PC 1.40
180
160
140
120
100
80
60
40
20
ppm
F
O
N
N
H
NO2
H2N
N
NH

^{13}C NMR of 6a

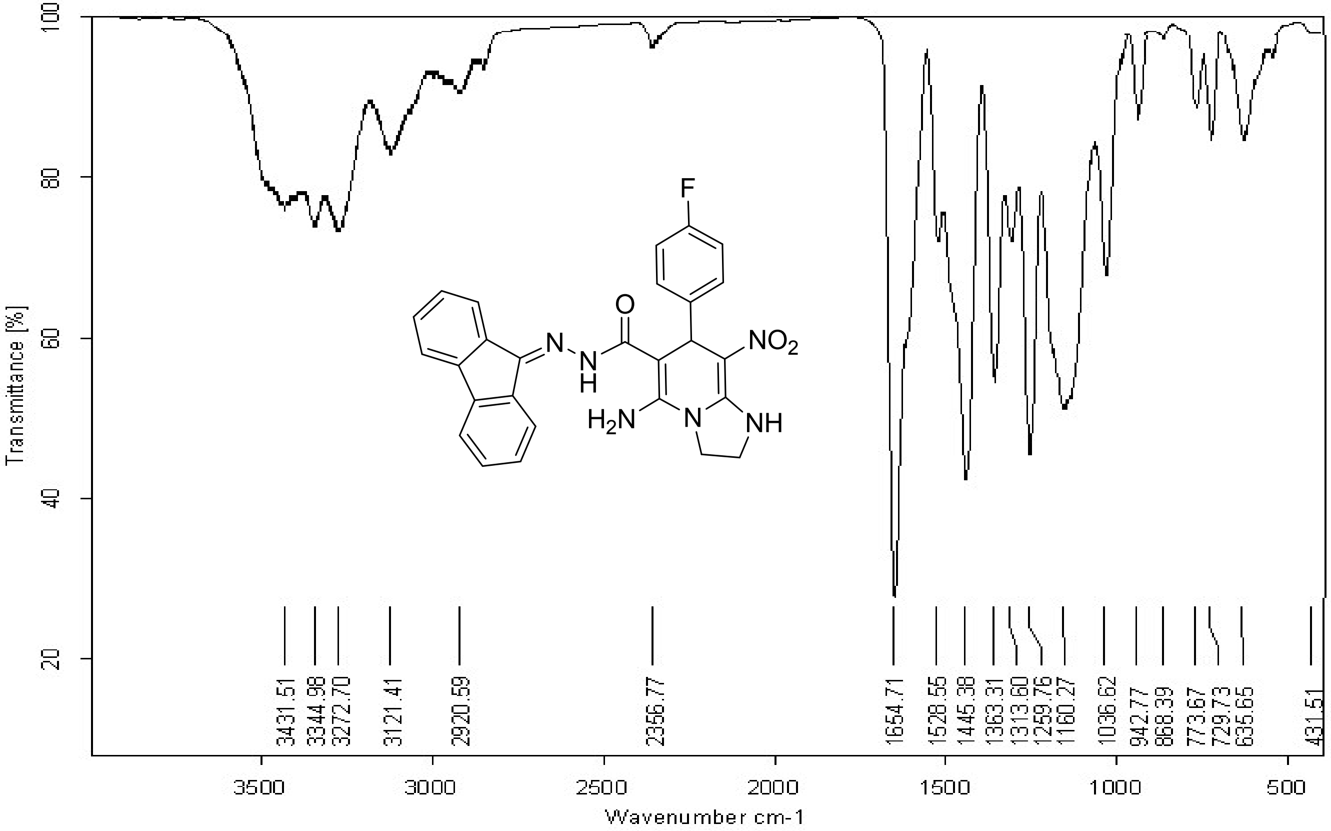

Transmittance [%]
100
80
60
40
20
Wavenumber cm-1
3500
3000
2500
2000
1500
1000
500
3431.51
3344.98
3272.70
3121.41
2920.59
2356.77
1654.71
1528.55
1445.38
1363.31
1313.60
1259.76
1160.27
1036.62
942.77
868.39
773.67
729.73
635.65
431.51
F
O
N
N
H
NO2
H2N
N
NH

IR of 6a

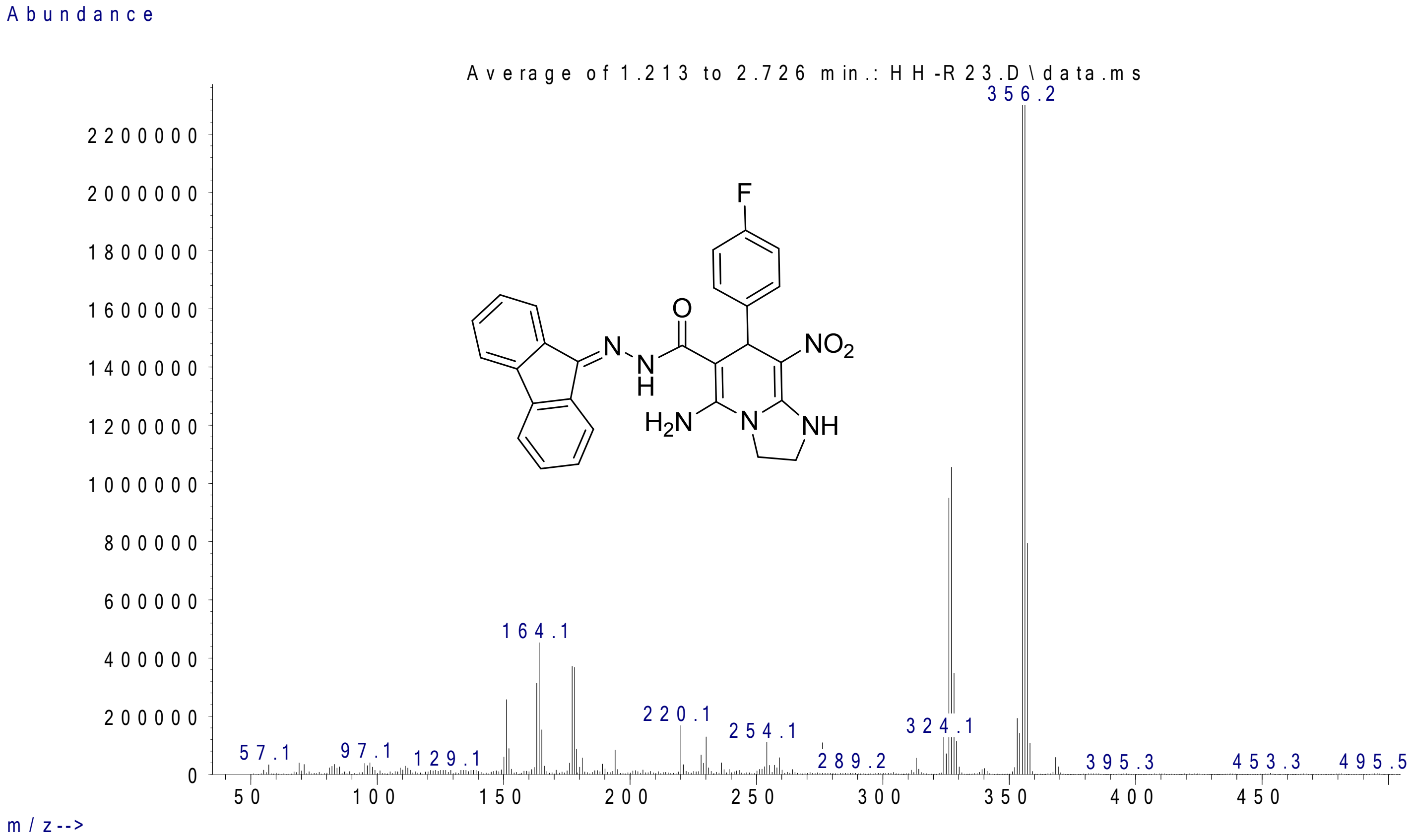
Abundance
Average of 1.213 to 2.726 min.: HH-R23.D\data.ms
356.2
2200000
2000000
1800000
1600000
1400000
1200000
1000000
800000
600000
400000
200000
0
164.1
220.1
254.1
289.2
324.1
57.1
97.1
129.1
395.3
453.3
495.5
50
100
150
200
250
300
350
400
450
m/z-->
F
O
N
N
H
NO2
H2N
N
NH

MS of 6a

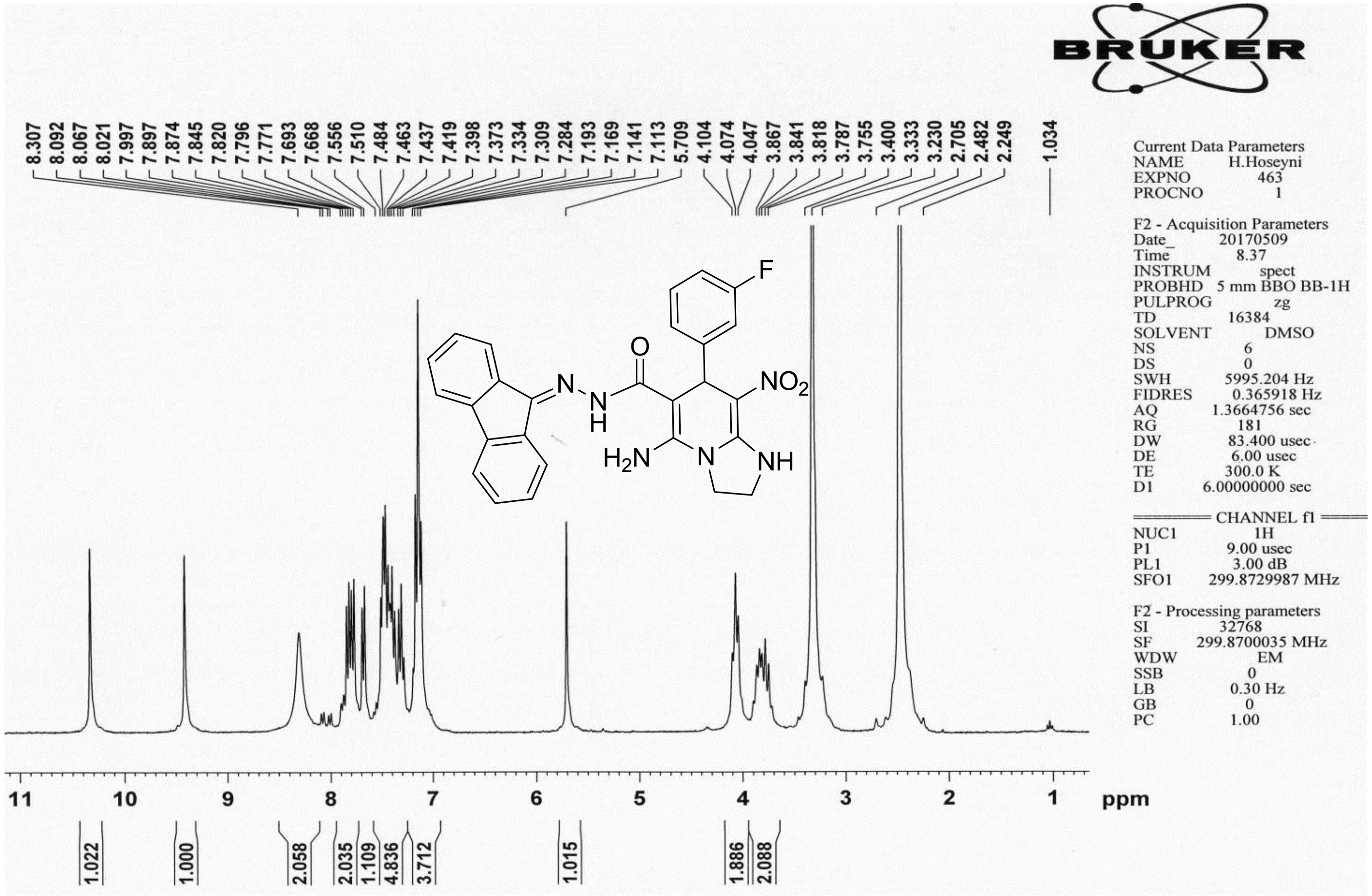
BRUKER
8.307 8.092 8.067 8.021 7.997 7.897 7.874 7.845 7.820 7.796 7.771 7.693 7.668 7.556 7.510 7.484 7.463 7.437 7.419 7.398 7.373 7.334 7.309 7.284 7.193 7.169 7.141 7.113 5.709 4.104 4.074 4.047 3.867 3.841 3.818 3.787 3.755 3.400 3.333 3.230 2.705 2.482 2.249 1.034
F
O
N
N
H
NO2
H2N
N
NH
11 10 9 8 7 6 5 4 3 2 1 ppm
1.022 1.000 2.058 2.035 1.109 4.836 3.712 1.015 1.886 2.088
Current Data Parameters
NAME H.Hoseyni
EXPNO 463
PROCNO 1
F2 - Acquisition Parameters
Date_ 20170509
Time 8.37
INSTRUM spect
PROBHD 5 mm BBO BB-1H
PULPROG zg
TD 16384
SOLVENT DMSO
NS 6
DS 0
SWH 5995.204 Hz
FIDRES 0.365918 Hz
AQ 1.3664756 sec
RG 181
DW 83.400 usec
DE 6.00 usec
TE 300.0 K
D1 6.00000000 sec
CHANNEL f1
NUC1 1H
P1 9.00 usec
PL1 3.00 dB
SFO1 299.8729987 MHz
F2 - Processing parameters
SI 32768
SF 299.8700035 MHz
WDW EM
SSB 0
LB 0.30 Hz
GB 0
PC 1.00

^{1}H NMR of 6b

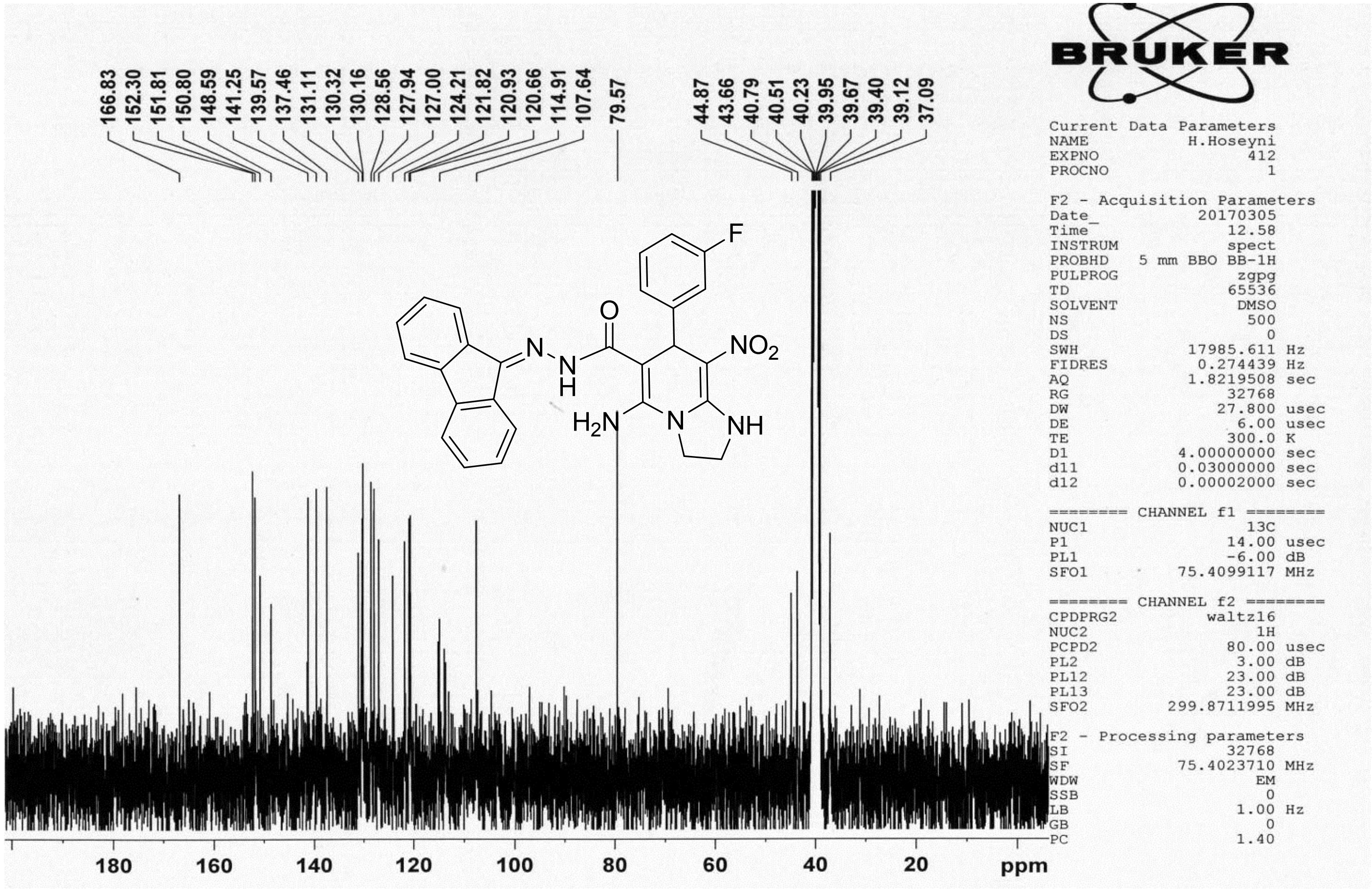

166.83
152.30
151.81
150.80
148.59
141.25
139.57
137.46
131.11
130.32
130.16
128.56
127.94
127.00
124.21
121.82
120.93
120.66
114.91
107.64
79.57
44.87
43.66
40.79
40.51
40.23
39.95
39.67
39.40
39.12
37.09
BRUKER
Current Data Parameters
NAME H.Hoseyni
EXPNO 412
PROCNO 1
F2 - Acquisition Parameters
Date_ 20170305
Time 12.58
INSTRUM spect
PROBHD 5 mm BBO BB-1H
PULPROG zgpg
TD 65536
SOLVENT DMSO
NS 500
DS 0
SWH 17985.611 Hz
FIDRES 0.274439 Hz
AQ 1.8219508 sec
RG 32768
DW 27.800 usec
DE 6.00 usec
TE 300.0 K
D1 4.00000000 sec
d11 0.03000000 sec
d12 0.00002000 sec
======== CHANNEL f1 ========
NUC1 13C
P1 14.00 usec
PL1 -6.00 dB
SFO1 75.4099117 MHz
======== CHANNEL f2 ========
CPDPRG2 waltz16
NUC2 1H
PCPD2 80.00 usec
PL2 3.00 dB
PL12 23.00 dB
PL13 23.00 dB
SFO2 299.8711995 MHz
F2 - Processing parameters
SI 32768
SF 75.4023710 MHz
WDW EM
SSB 0
LB 1.00 Hz
GB 0
PC 1.40
F
O
N
N
H
NO2
H2N
N
NH
180 160 140 120 100 80 60 40 20 ppm

^{13}C NMR of 6b

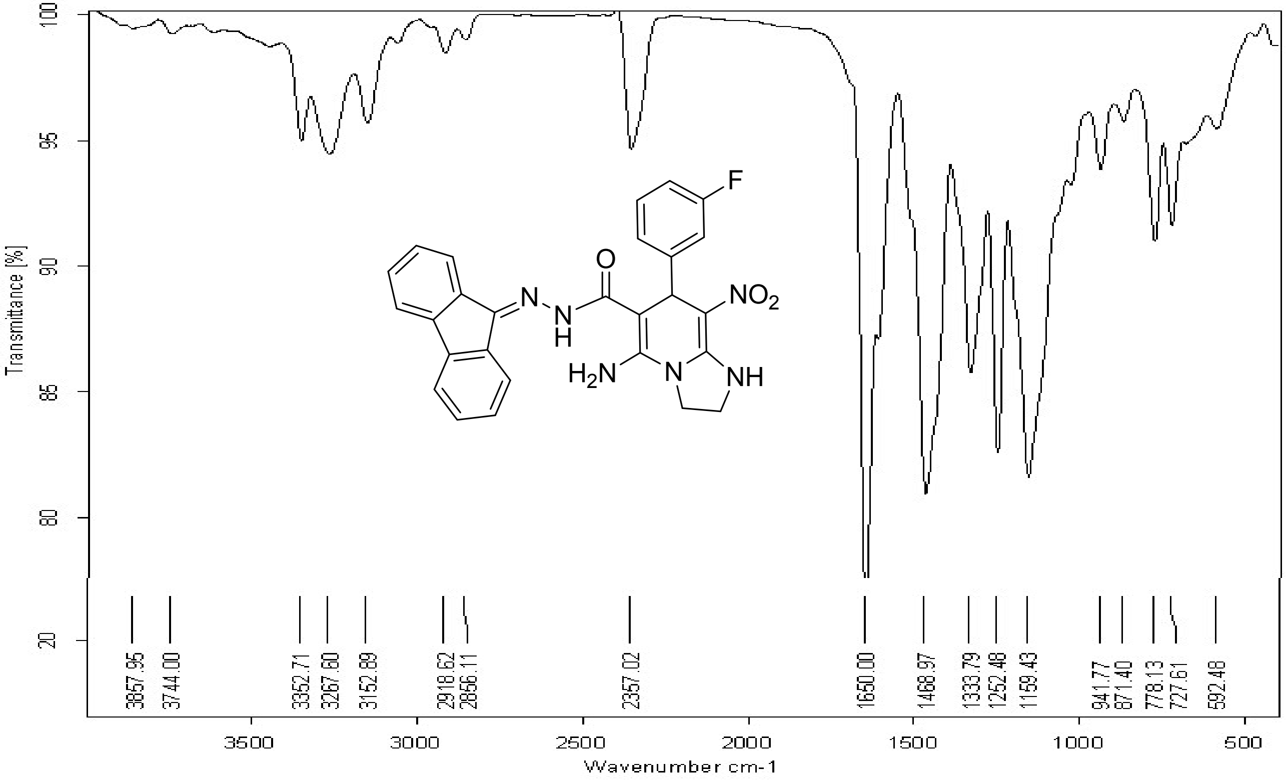
Transmittance [%]
100
95
90
85
80
20
Wavenumber cm-1
3500
3000
2500
2000
1500
1000
500
3857.95
3744.00
3352.71
3267.60
3152.89
2918.62
2856.11
2357.02
1650.00
1468.97
1333.79
1252.48
1159.43
941.77
871.40
778.13
727.61
592.48
F
O
NO2
N
N
H
H2N
N
NH

IR of 6b

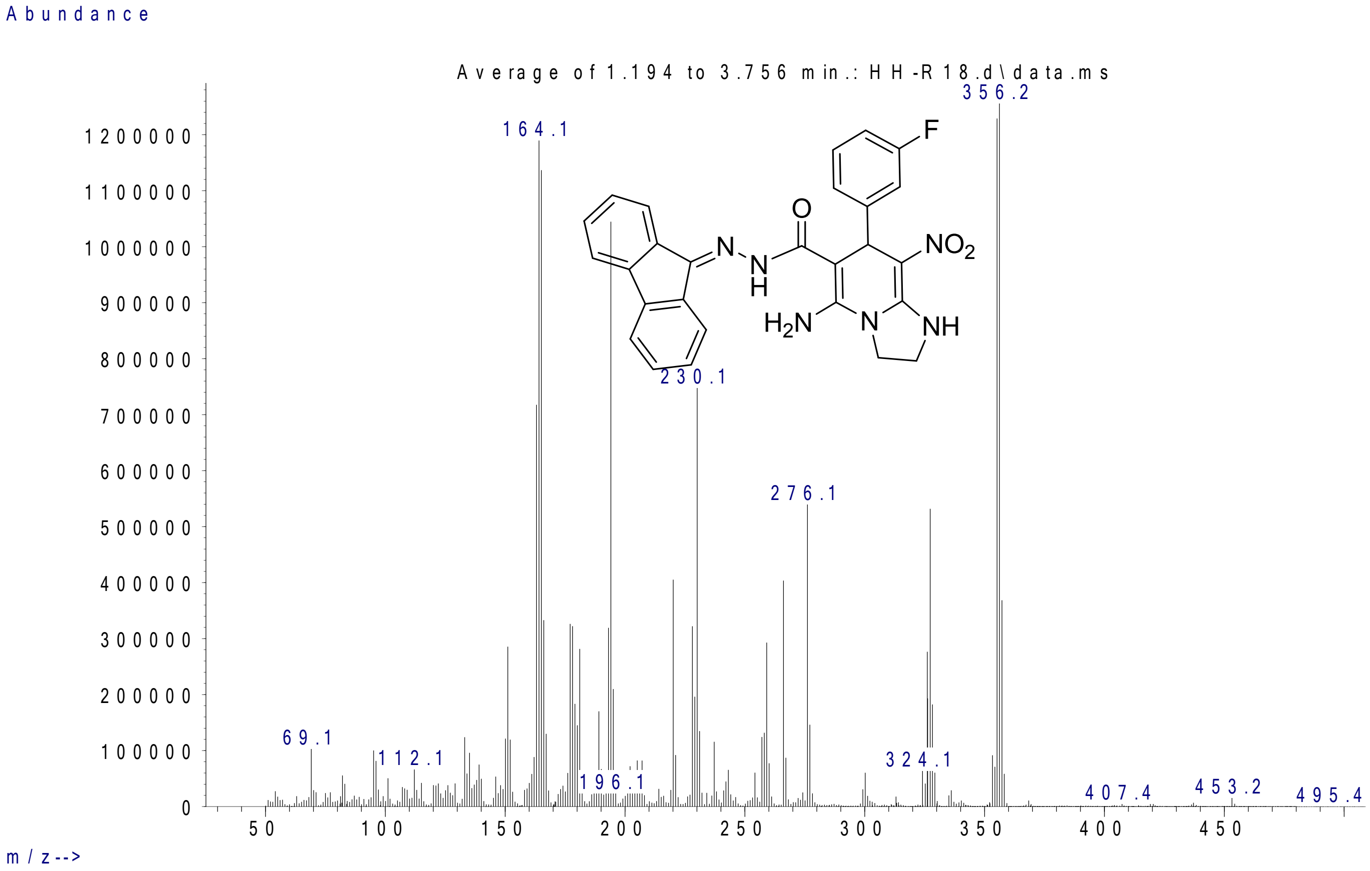
Abundance
Average of 1.194 to 3.756 min.: HH-R18.d\data.ms
356.2
164.1
230.1
276.1
69.1
112.1
196.1
324.1
407.4
453.2
495.4
1200000
1100000
1000000
900000
800000
700000
600000
500000
400000
300000
200000
100000
0
50
100
150
200
250
300
350
400
450
m/z-->
F
O
N
N
H
NO_2
H_2N
N
NH

MS of 6b

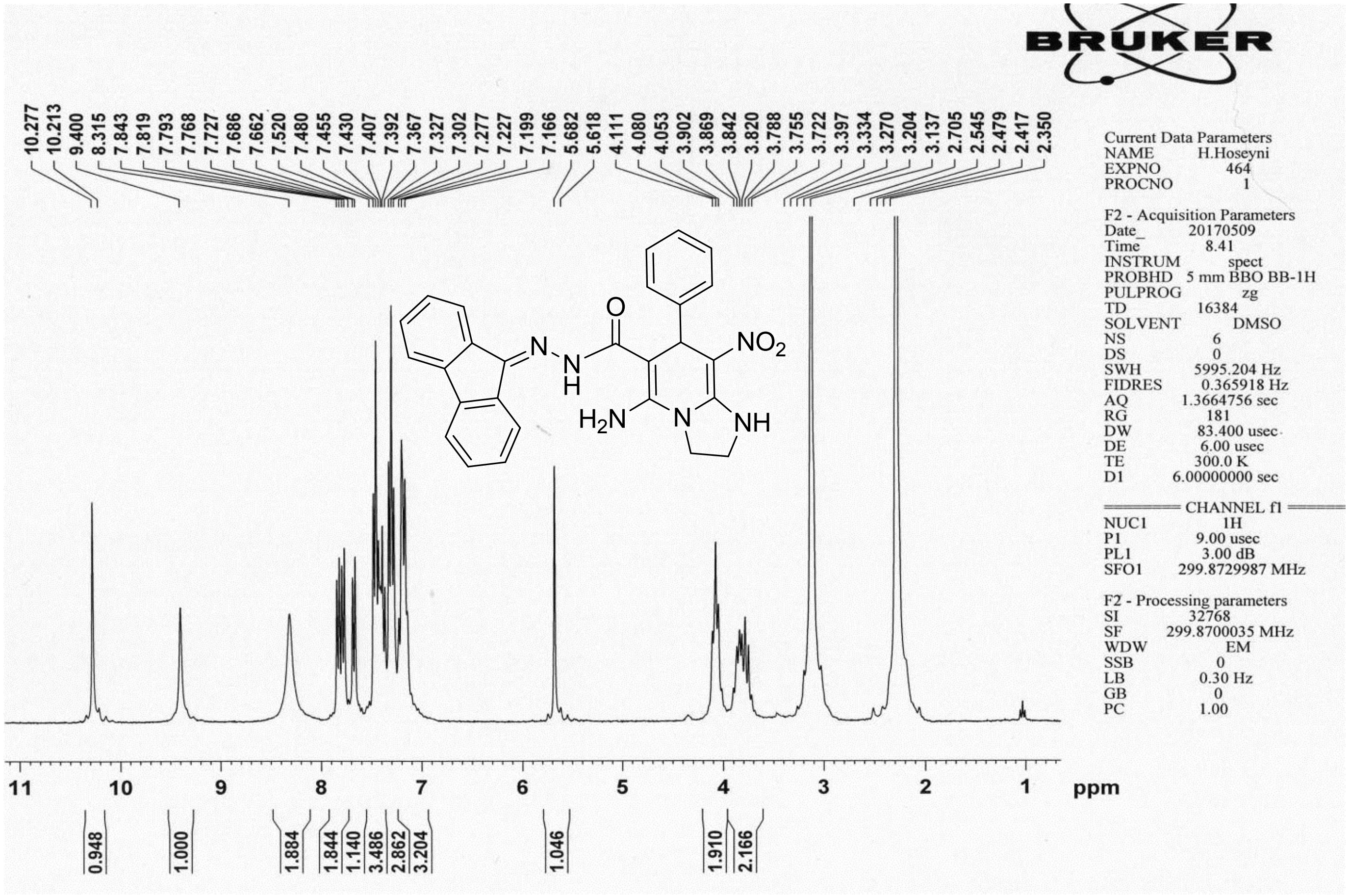

BRUKER
10.277 10.213 9.400 8.315 7.843 7.819 7.793 7.768 7.727 7.686 7.662 7.520 7.480 7.455 7.430 7.407 7.392 7.367 7.327 7.302 7.277 7.227 7.199 7.166 5.682 5.618 4.111 4.080 4.053 3.902 3.869 3.842 3.820 3.788 3.755 3.722 3.397 3.334 3.270 3.204 3.137 2.705 2.545 2.479 2.417 2.350
Current Data Parameters
NAME H.Hoseyni
EXPNO 464
PROCNO 1
F2 - Acquisition Parameters
Date_ 20170509
Time 8.41
INSTRUM spect
PROBHD 5 mm BBO BB-1H
PULPROG zg
TD 16384
SOLVENT DMSO
NS 6
DS 0
SWH 5995.204 Hz
FIDRES 0.365918 Hz
AQ 1.3664756 sec
RG 181
DW 83.400 usec
DE 6.00 usec
TE 300.0 K
D1 6.00000000 sec
CHANNEL f1
NUC1 1H
P1 9.00 usec
PL1 3.00 dB
SFO1 299.8729987 MHz
F2 - Processing parameters
SI 32768
SF 299.8700035 MHz
WDW EM
SSB 0
LB 0.30 Hz
GB 0
PC 1.00
NO2
H2N
NH
O
N
H
11 10 9 8 7 6 5 4 3 2 1 ppm
0.948 1.000 1.884 1.844 1.140 3.486 2.862 3.204 1.046 1.910 2.166

^{1}H NMR of 6c

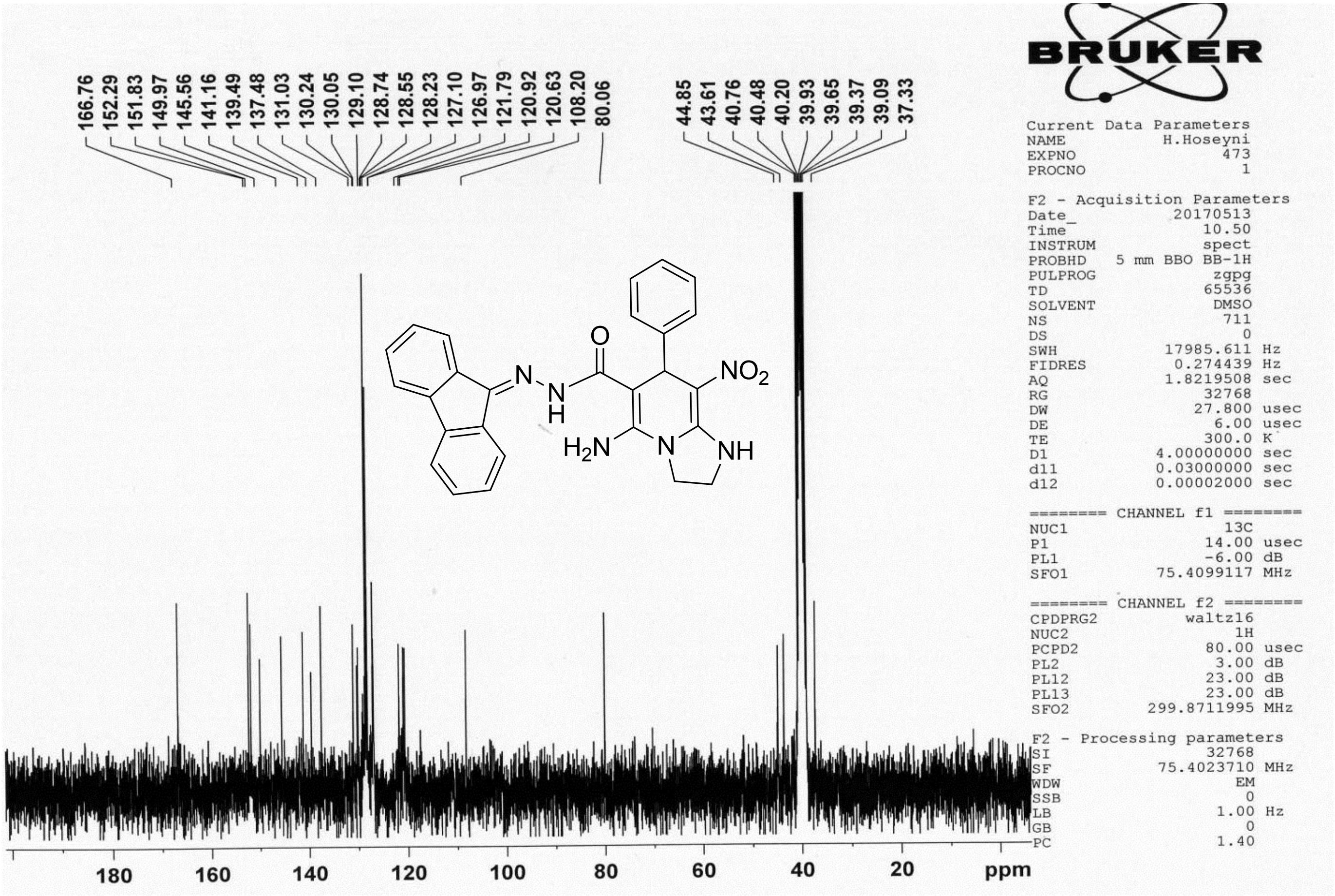

166.76
152.29
151.83
149.97
145.56
141.16
139.49
137.48
131.03
130.24
130.05
129.10
128.74
128.55
128.23
127.10
126.97
121.79
120.92
120.63
108.20
80.06
44.85
43.61
40.76
40.48
40.20
39.93
39.65
39.37
39.09
37.33
BRUKER
Current Data Parameters
NAME H.Hoseyni
EXPNO 473
PROCNO 1
F2 - Acquisition Parameters
Date_ 20170513
Time 10.50
INSTRUM spect
PROBHD 5 mm BBO BB-1H
PULPROG zgpg
TD 65536
SOLVENT DMSO
NS 711
DS 0
SWH 17985.611 Hz
FIDRES 0.274439 Hz
AQ 1.8219508 sec
RG 32768
DW 27.800 usec
DE 6.00 usec
TE 300.0 K
D1 4.00000000 sec
d11 0.03000000 sec
d12 0.00002000 sec
======== CHANNEL f1 ========
NUC1 13C
P1 14.00 usec
PL1 -6.00 dB
SFO1 75.4099117 MHz
======== CHANNEL f2 ========
CPDPRG2 waltz16
NUC2 1H
PCPD2 80.00 usec
PL2 3.00 dB
PL12 23.00 dB
PL13 23.00 dB
SFO2 299.8711995 MHz
F2 - Processing parameters
SI 32768
SF 75.4023710 MHz
WDW EM
SSB 0
LB 1.00 Hz
GB 0
PC 1.40
O
N
N
H
NO2
H2N
N
NH
180
160
140
120
100
80
60
40
20
ppm

^{13}C NMR of 6c

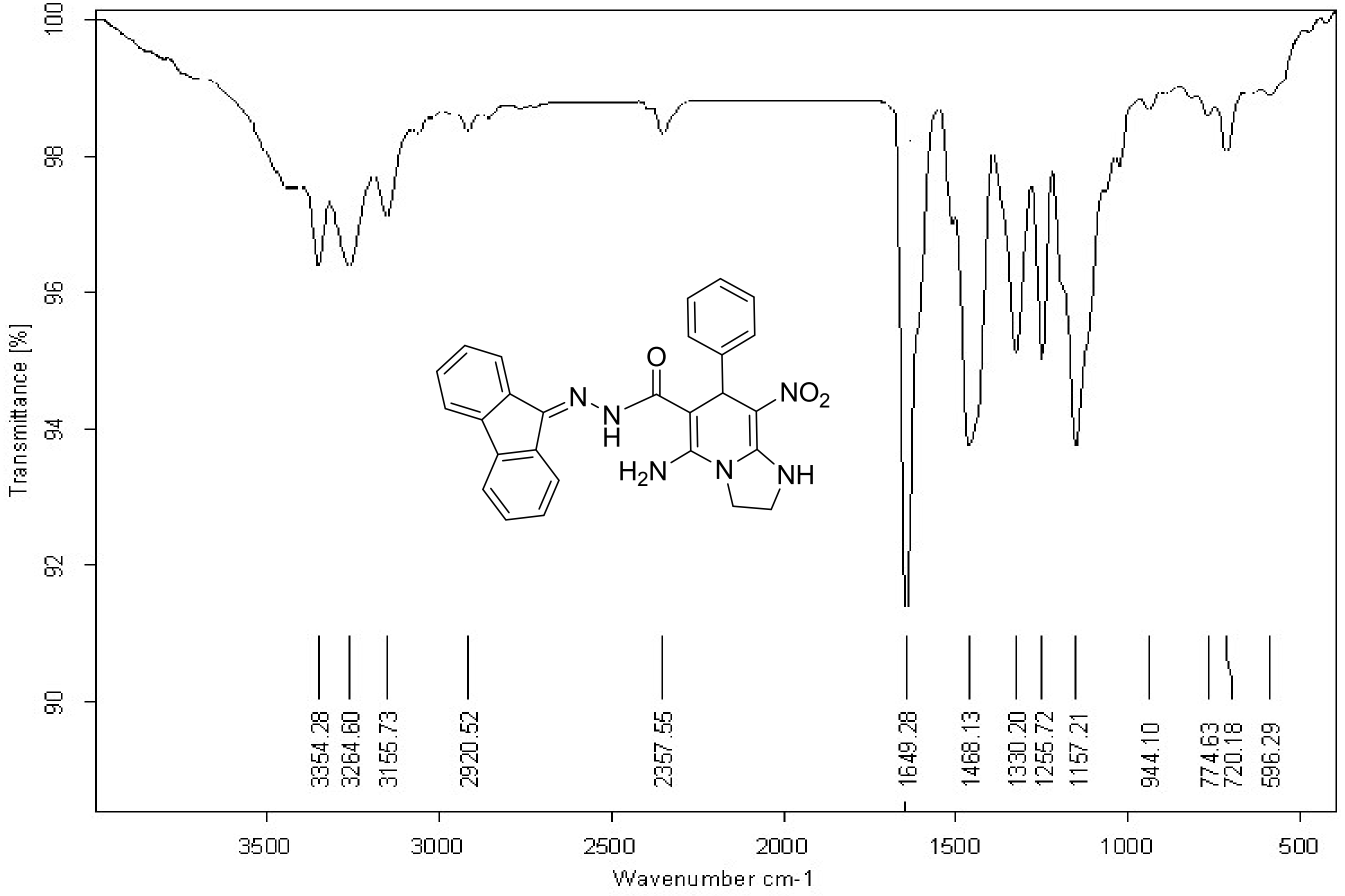

Transmittance [%]
100
98
96
94
92
90
Wavenumber cm-1
3500
3000
2500
2000
1500
1000
500
3354.28
3264.60
3155.73
2920.52
2357.55
1649.28
1468.13
1330.20
1255.72
1157.21
944.10
774.63
720.18
596.29
O
N
N
H
NO2
H2N
N
NH

IR of 6c

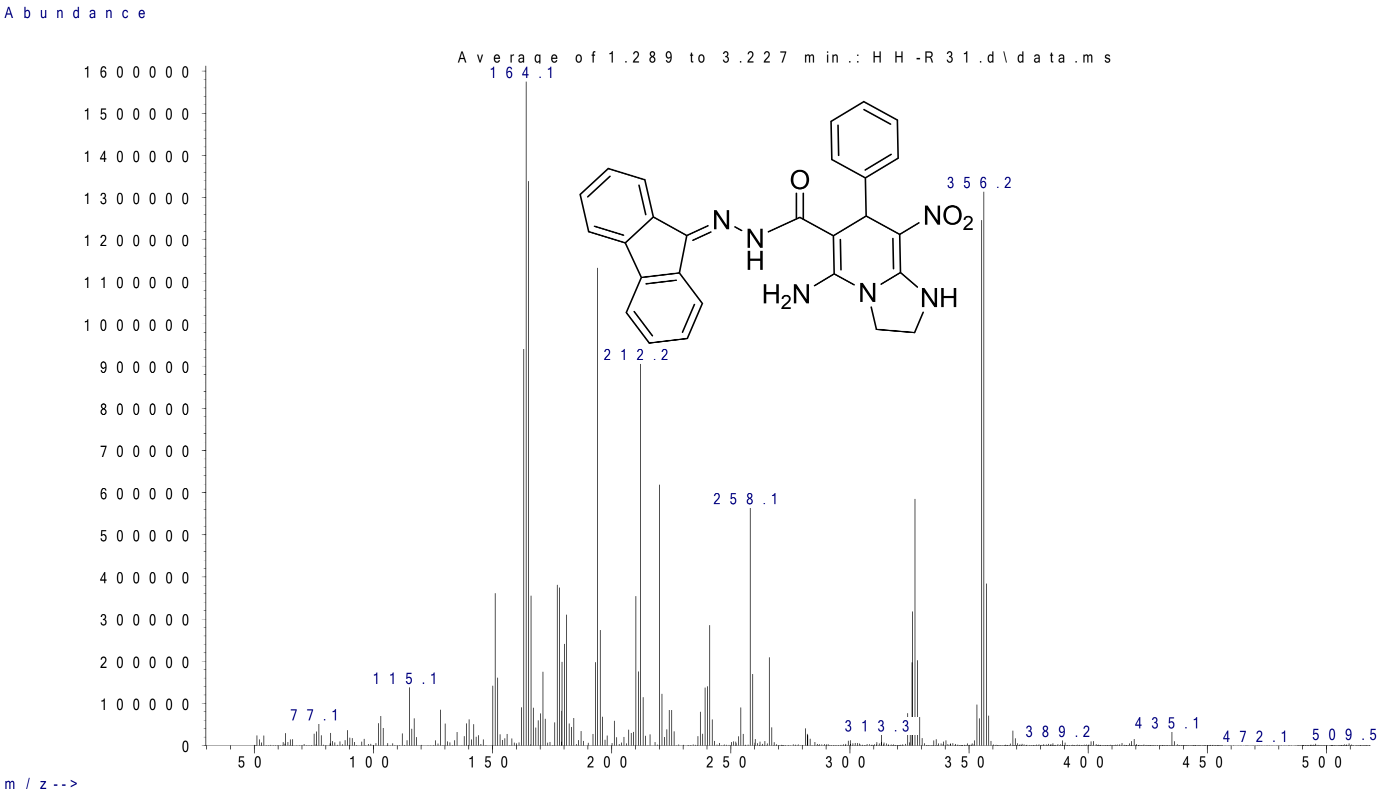

Abundance
Average of 1.289 to 3.227 min.: HH-R31.d\data.ms
1600000
1500000
1400000
1300000
1200000
1100000
1000000
900000
800000
700000
600000
500000
400000
300000
200000
100000
0
164.1
356.2
212.2
258.1
115.1
77.1
313.3
389.2
435.1
472.1
509.5
50
100
150
200
250
300
350
400
450
500
m/z-->
O
N
N
H
NO_2
H_2N
N
NH

MS of 6c

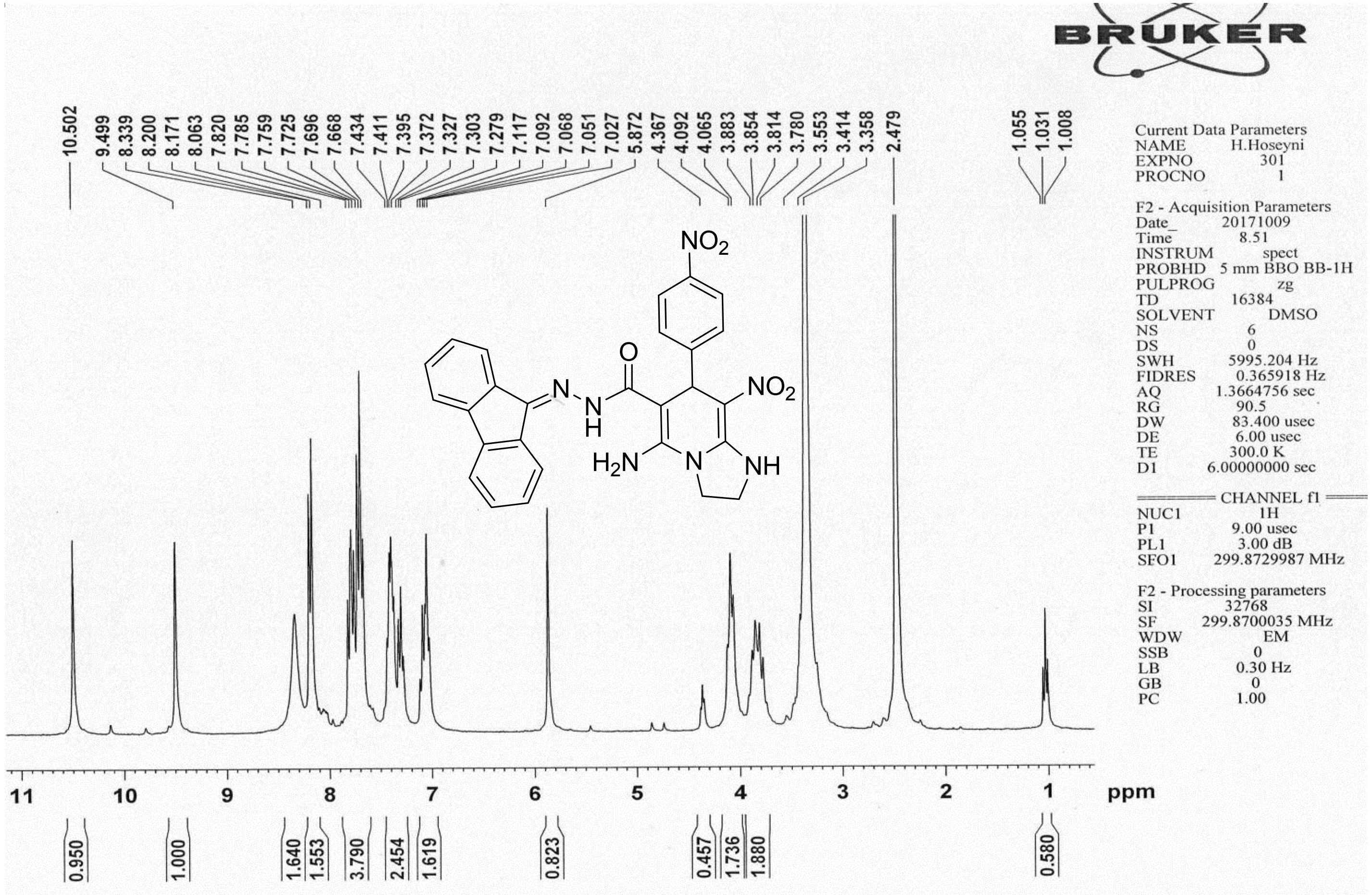

BRUKER
10.502
9.499
8.339
8.200
8.171
8.063
7.820
7.785
7.759
7.725
7.696
7.668
7.434
7.411
7.395
7.372
7.327
7.303
7.279
7.117
7.092
7.068
7.051
7.027
5.872
4.367
4.092
4.065
3.883
3.854
3.814
3.780
3.553
3.414
3.358
2.479
1.055
1.031
1.008
NO_2
O
N
N
H
NO_2
H_2N
N
NH
Current Data Parameters
NAME H.Hoseyni
EXPNO 301
PROCNO 1
F2 - Acquisition Parameters
Date_ 20171009
Time 8.51
INSTRUM spect
PROBHD 5 mm BBO BB-1H
PULPROG zg
TD 16384
SOLVENT DMSO
NS 6
DS 0
SWH 5995.204 Hz
FIDRES 0.365918 Hz
AQ 1.3664756 sec
RG 90.5
DW 83.400 usec
DE 6.00 usec
TE 300.0 K
D1 6.00000000 sec
CHANNEL f1
NUC1 1H
P1 9.00 usec
PL1 3.00 dB
SFO1 299.8729987 MHz
F2 - Processing parameters
SI 32768
SF 299.8700035 MHz
WDW EM
SSB 0
LB 0.30 Hz
GB 0
PC 1.00
11 10 9 8 7 6 5 4 3 2 1 ppm
0.950
1.000
1.640
1.553
3.790
2.454
1.619
0.823
0.457
1.736
1.880
0.580

^1H NMR of 6d

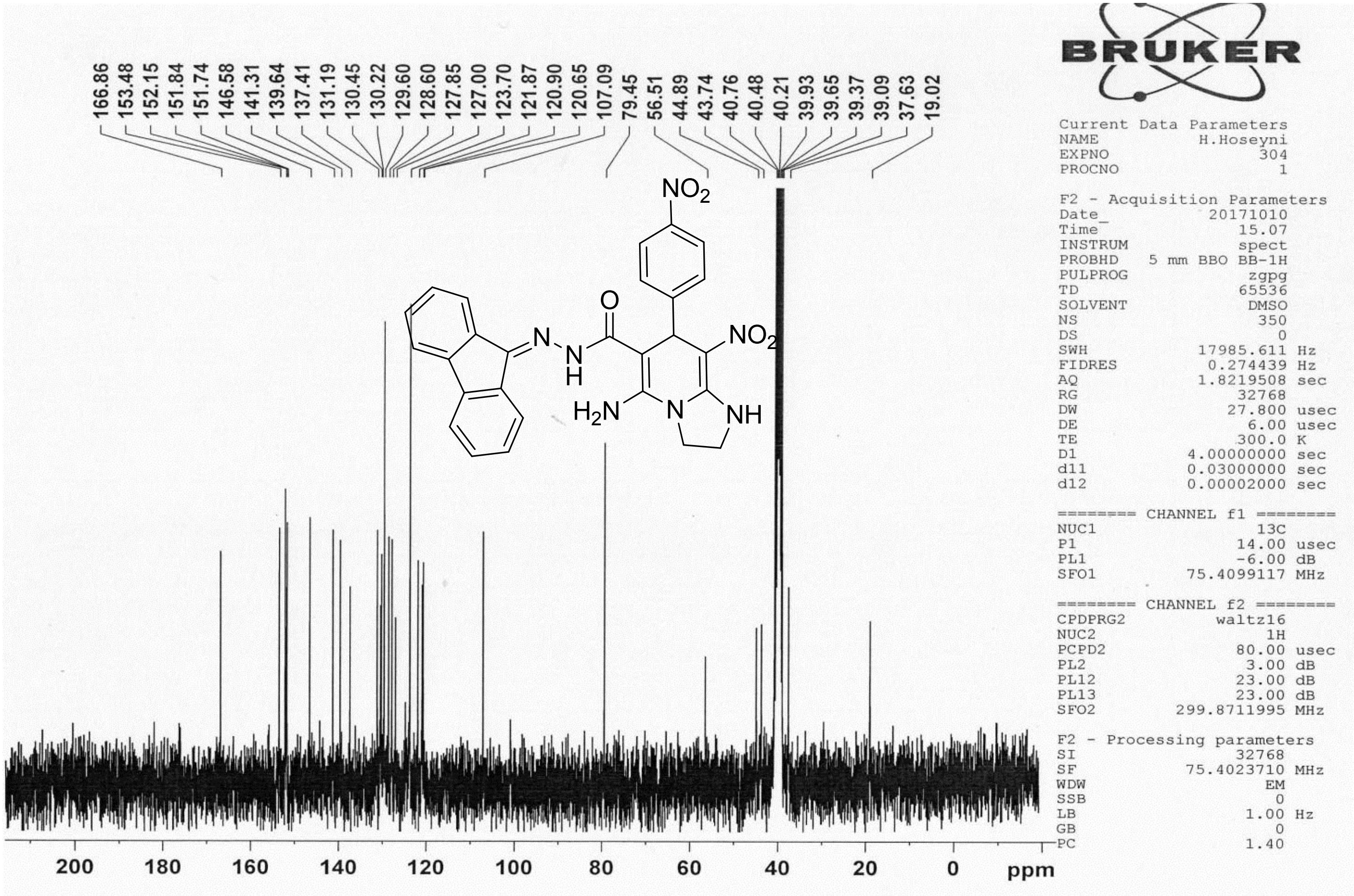
166.86
153.48
152.15
151.84
151.74
146.58
141.31
139.64
137.41
131.19
130.45
130.22
129.60
128.60
127.85
127.00
123.70
121.87
120.90
120.65
107.09
79.45
56.51
44.89
43.74
40.76
40.48
40.21
39.93
39.65
39.37
39.09
37.63
19.02
BRUKER
Current Data Parameters
NAME H.Hoseyni
EXPNO 304
PROCNO 1
F2 - Acquisition Parameters
Date_ 20171010
Time 15.07
INSTRUM spect
PROBHD 5 mm BBO BB-1H
PULPROG zgpg
TD 65536
SOLVENT DMSO
NS 350
DS 0
SWH 17985.611 Hz
FIDRES 0.274439 Hz
AQ 1.8219508 sec
RG 32768
DW 27.800 usec
DE 6.00 usec
TE 300.0 K
D1 4.00000000 sec
d11 0.03000000 sec
d12 0.00002000 sec
======== CHANNEL f1 ========
NUC1 13C
P1 14.00 usec
PL1 -6.00 dB
SFO1 75.4099117 MHz
======== CHANNEL f2 ========
CPDPRG2 waltz16
NUC2 1H
PCPD2 80.00 usec
PL2 3.00 dB
PL12 23.00 dB
PL13 23.00 dB
SFO2 299.8711995 MHz
F2 - Processing parameters
SI 32768
SF 75.4023710 MHz
WDW EM
SSB 0
LB 1.00 Hz
GB 0
PC 1.40
NO2
O
N
N
H
NO2
H2N
N
NH
200
180
160
140
120
100
80
60
40
20
0
ppm

^{13}C NMR of 6d

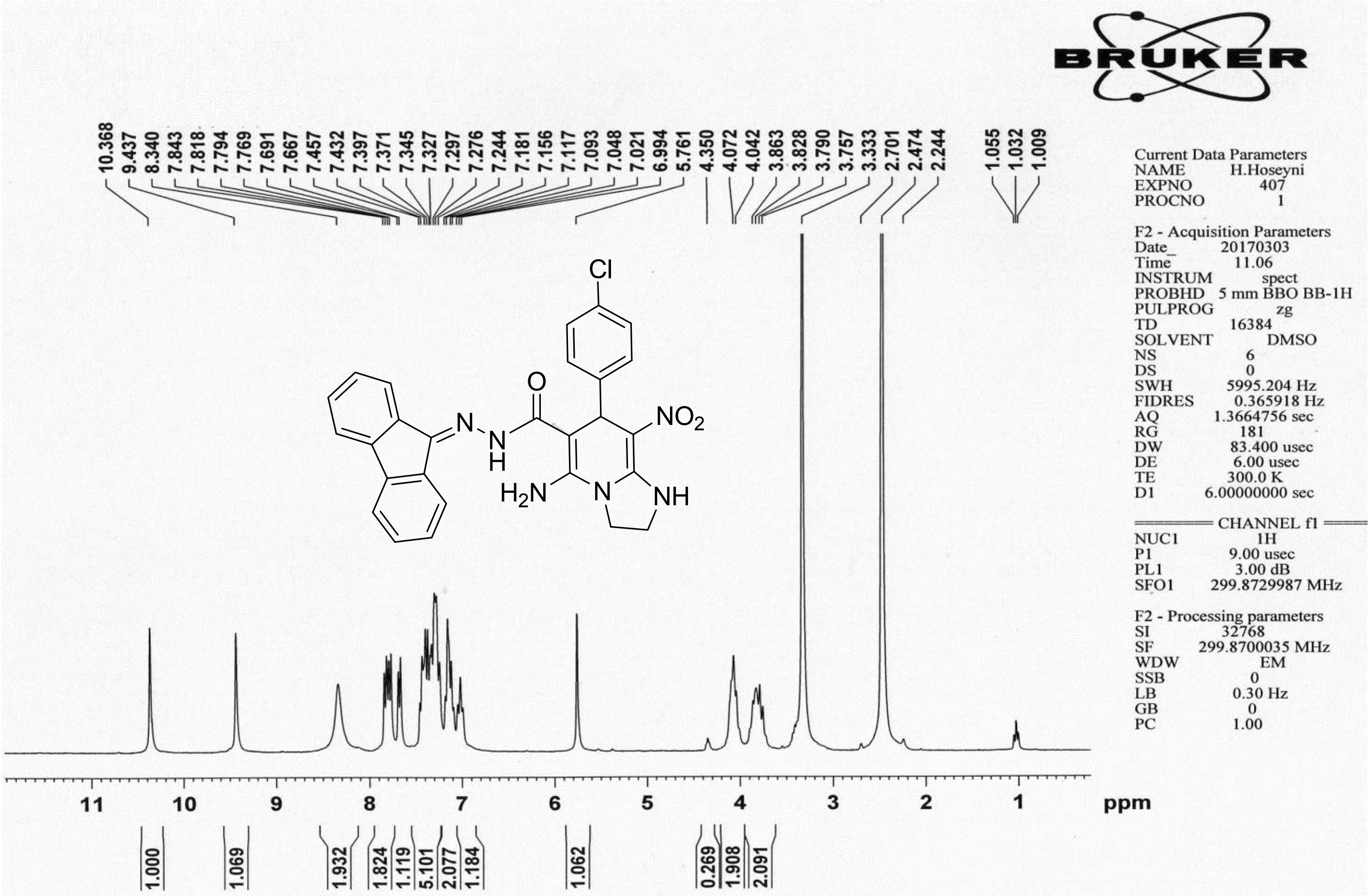
BRUKER
Current Data Parameters
NAME H.Hoseyni
EXPNO 407
PROCNO 1
F2 - Acquisition Parameters
Date_ 20170303
Time 11.06
INSTRUM spect
PROBHD 5 mm BBO BB-1H
PULPROG zg
TD 16384
SOLVENT DMSO
NS 6
DS 0
SWH 5995.204 Hz
FIDRES 0.365918 Hz
AQ 1.3664756 sec
RG 181
DW 83.400 usec
DE 6.00 usec
TE 300.0 K
D1 6.00000000 sec
CHANNEL f1
NUC1 1H
P1 9.00 usec
PL1 3.00 dB
SFO1 299.8729987 MHz
F2 - Processing parameters
SI 32768
SF 299.8700035 MHz
WDW EM
SSB 0
LB 0.30 Hz
GB 0
PC 1.00
ppm

^{1}H NMR of 6e

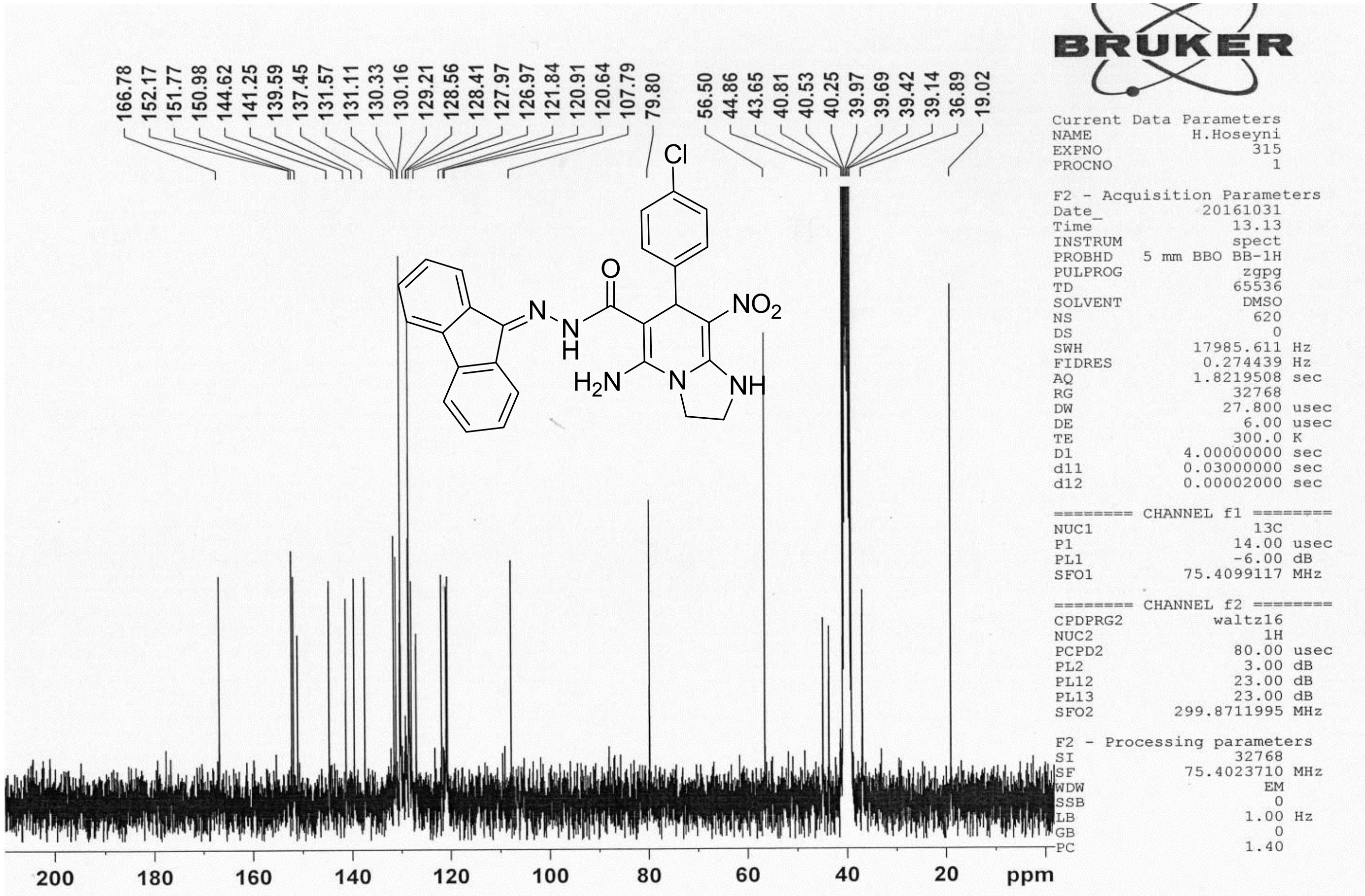

^{13}C NMR of 6e

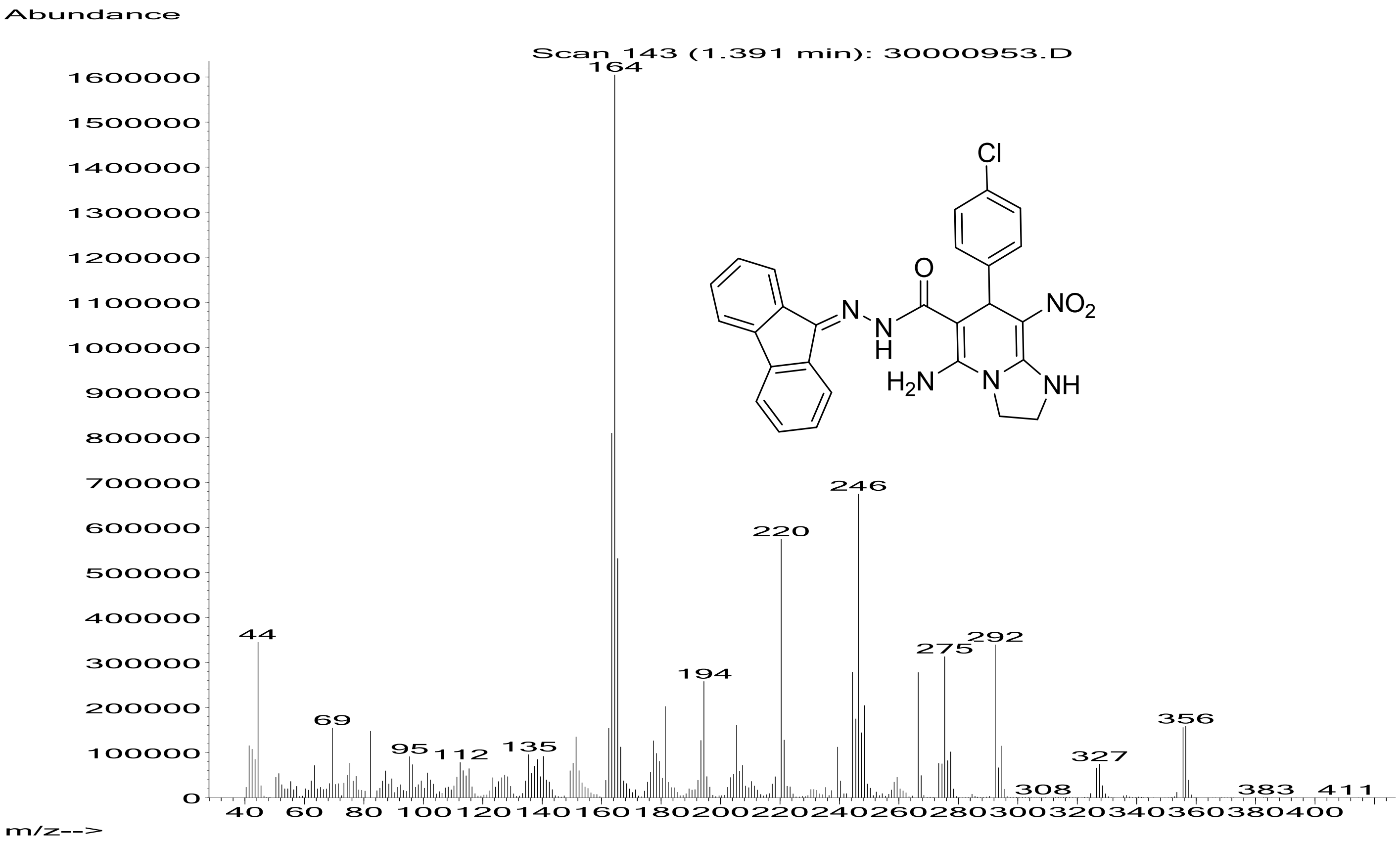
Abundance
Scan 143 (1.391 min): 30000953.D
1600000
1500000
1400000
1300000
1200000
1100000
1000000
900000
800000
700000
600000
500000
400000
300000
200000
100000
0
164
246
220
44
275
292
194
69
95
112
135
356
327
308
383
411
40 60 80 100 120 140 160 180 200 220 240 260 280 300 320 340 360 380 400
m/z-->
Cl
O
NO2
N
N
H
H2N
N
NH

MS of 6e

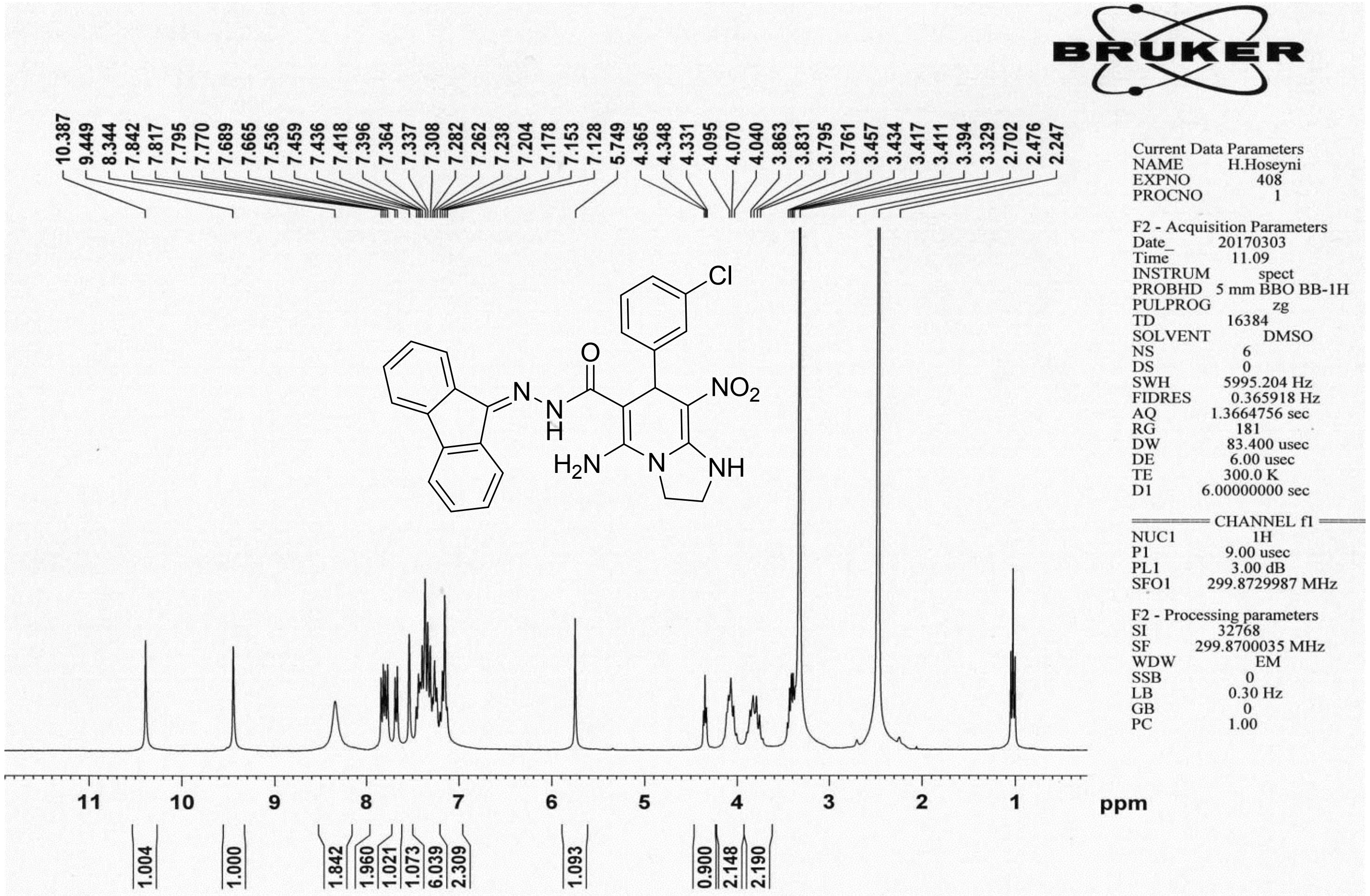
BRUKER
10.387 9.449 8.344 7.842 7.817 7.795 7.770 7.689 7.665 7.536 7.459 7.436 7.418 7.396 7.364 7.337 7.308 7.282 7.262 7.238 7.204 7.178 7.153 7.128 5.749 4.365 4.348 4.331 4.095 4.070 4.040 3.863 3.831 3.795 3.761 3.457 3.434 3.417 3.411 3.394 3.329 2.702 2.476 2.247
Current Data Parameters
NAME H.Hoseyni
EXPNO 408
PROCNO 1
F2 - Acquisition Parameters
Date_ 20170303
Time 11.09
INSTRUM spect
PROBHD 5 mm BBO BB-1H
PULPROG zg
TD 16384
SOLVENT DMSO
NS 6
DS 0
SWH 5995.204 Hz
FIDRES 0.365918 Hz
AQ 1.3664756 sec
RG 181
DW 83.400 usec
DE 6.00 usec
TE 300.0 K
D1 6.00000000 sec
CHANNEL f1
NUC1 1H
P1 9.00 usec
PL1 3.00 dB
SFO1 299.8729987 MHz
F2 - Processing parameters
SI 32768
SF 299.8700035 MHz
WDW EM
SSB 0
LB 0.30 Hz
GB 0
PC 1.00
Cl
O
N
N
H
NO2
H2N
N
NH
11 10 9 8 7 6 5 4 3 2 1 ppm
1.004 1.000 1.842 1.960 1.021 1.073 6.039 2.309 1.093 0.900 2.148 2.190

^{1}H NMR of 6f

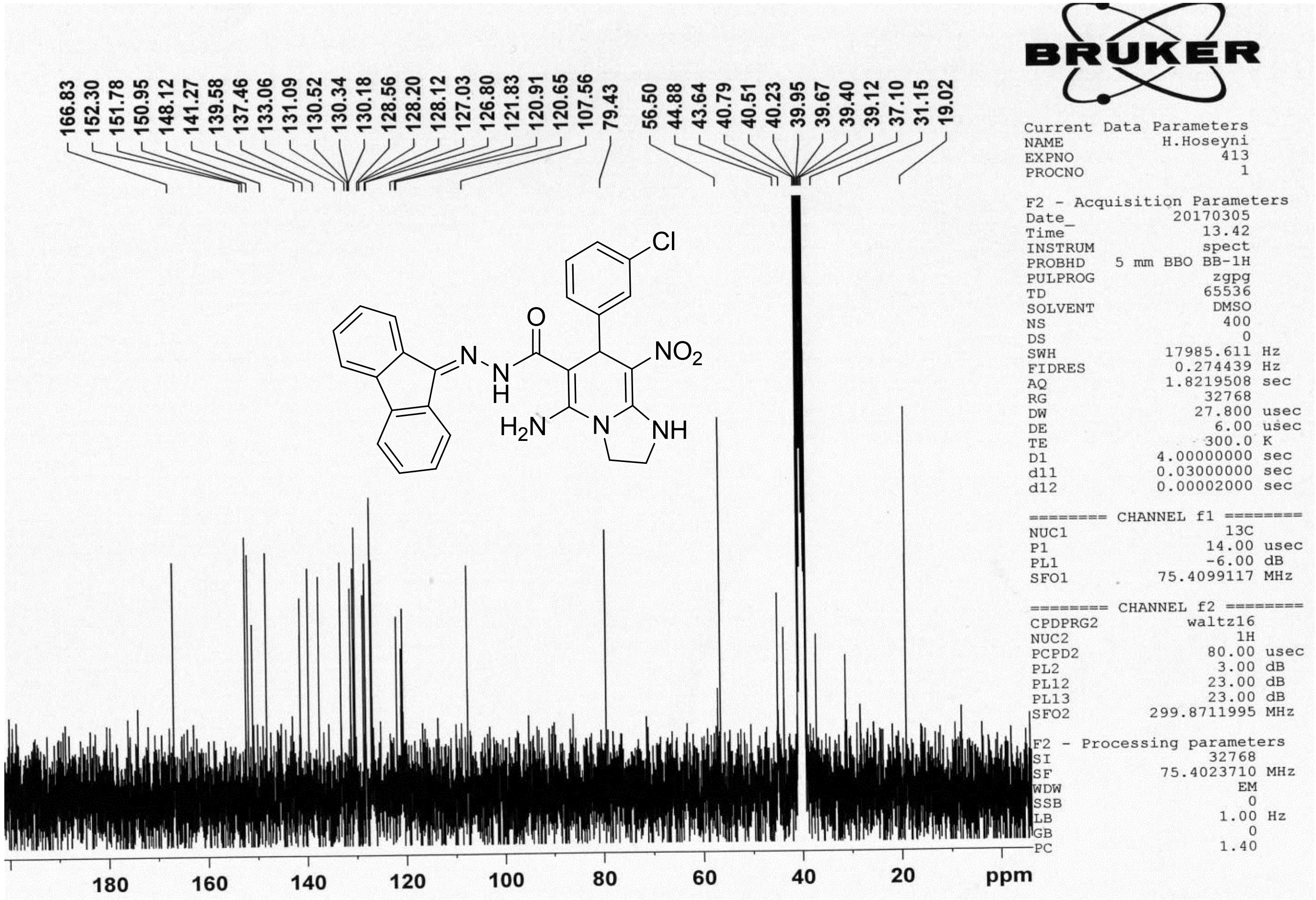

166.83
152.30
151.78
150.95
148.12
141.27
139.58
137.46
133.06
131.09
130.52
130.34
130.18
128.56
128.20
128.12
127.03
126.80
121.83
120.91
120.65
107.56
79.43
56.50
44.88
43.64
40.79
40.51
40.23
39.95
39.67
39.40
39.12
37.10
31.15
19.02
BRUKER
Current Data Parameters
NAME H.Hoseyni
EXPNO 413
PROCNO 1
F2 - Acquisition Parameters
Date_ 20170305
Time 13.42
INSTRUM spect
PROBHD 5 mm BBO BB-1H
PULPROG zgpg
TD 65536
SOLVENT DMSO
NS 400
DS 0
SWH 17985.611 Hz
FIDRES 0.274439 Hz
AQ 1.8219508 sec
RG 32768
DW 27.800 usec
DE 6.00 usec
TE 300.0 K
D1 4.00000000 sec
d11 0.03000000 sec
d12 0.00002000 sec
======== CHANNEL f1 ========
NUC1 13C
P1 14.00 usec
PL1 -6.00 dB
SFO1 75.4099117 MHz
======== CHANNEL f2 ========
CPDPRG2 waltz16
NUC2 1H
PCPD2 80.00 usec
PL2 3.00 dB
PL12 23.00 dB
PL13 23.00 dB
SFO2 299.8711995 MHz
F2 - Processing parameters
SI 32768
SF 75.4023710 MHz
WDW EM
SSB 0
LB 1.00 Hz
GB 0
PC 1.40
180
160
140
120
100
80
60
40
20
ppm

^{13}C NMR of 6f

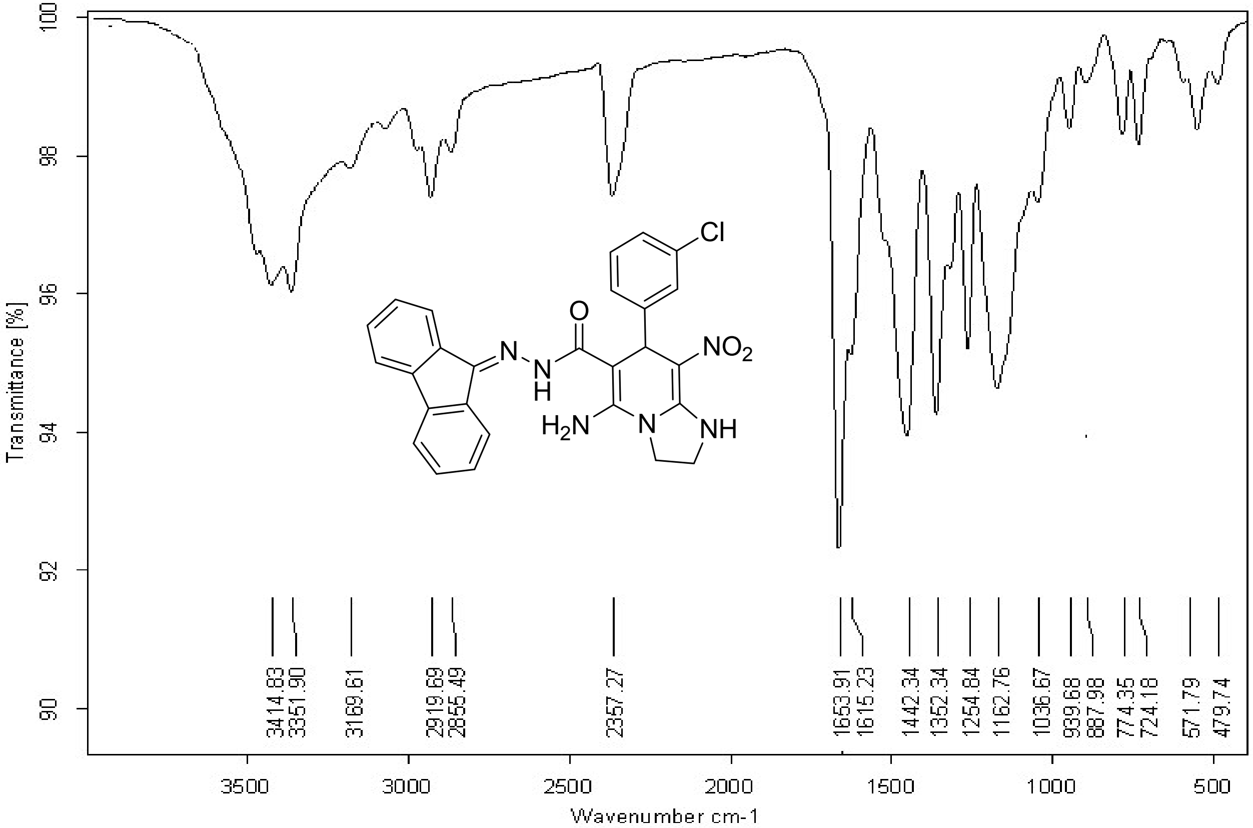

Transmittance [%]
100
98
96
94
92
90
3414.83
3351.90
3169.61
2919.69
2855.49
2357.27
1653.91
1615.23
1442.34
1352.34
1254.84
1162.76
1036.67
939.68
887.98
774.35
724.18
571.79
479.74
3500
3000
2500
2000
1500
1000
500
Wavenumber cm-1
Cl
O
N
N
H
NO2
H2N
N
NH

IR of 6f

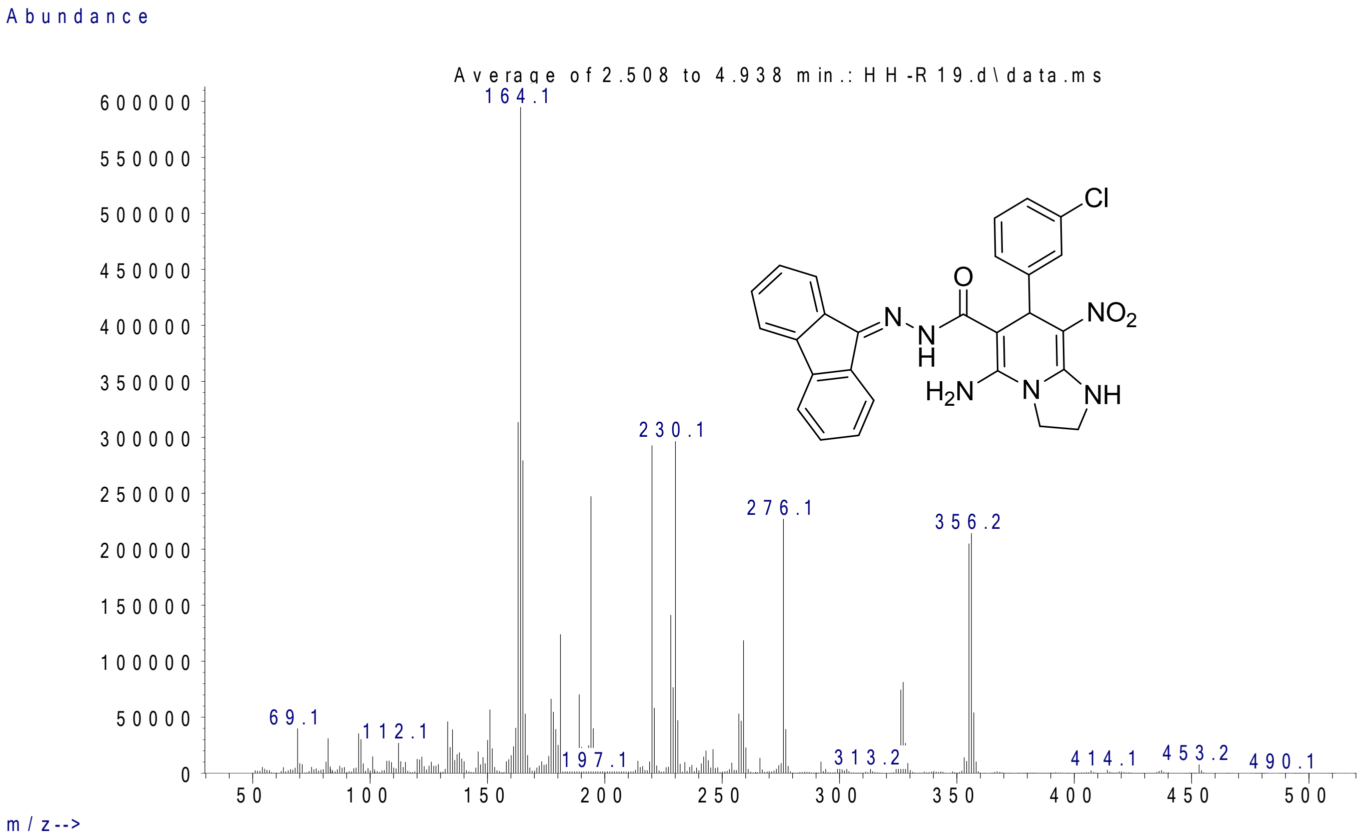
Abundance
Average of 2.508 to 4.938 min.: HH-R19.d\data.ms
600000
550000
500000
450000
400000
350000
300000
250000
200000
150000
100000
50000
0
164.1
230.1
276.1
356.2
69.1
112.1
197.1
313.2
414.1
453.2
490.1
50
100
150
200
250
300
350
400
450
500
m/z-->
Cl
O
N
N
H
NO2
H2N
N
NH

MS of 6f

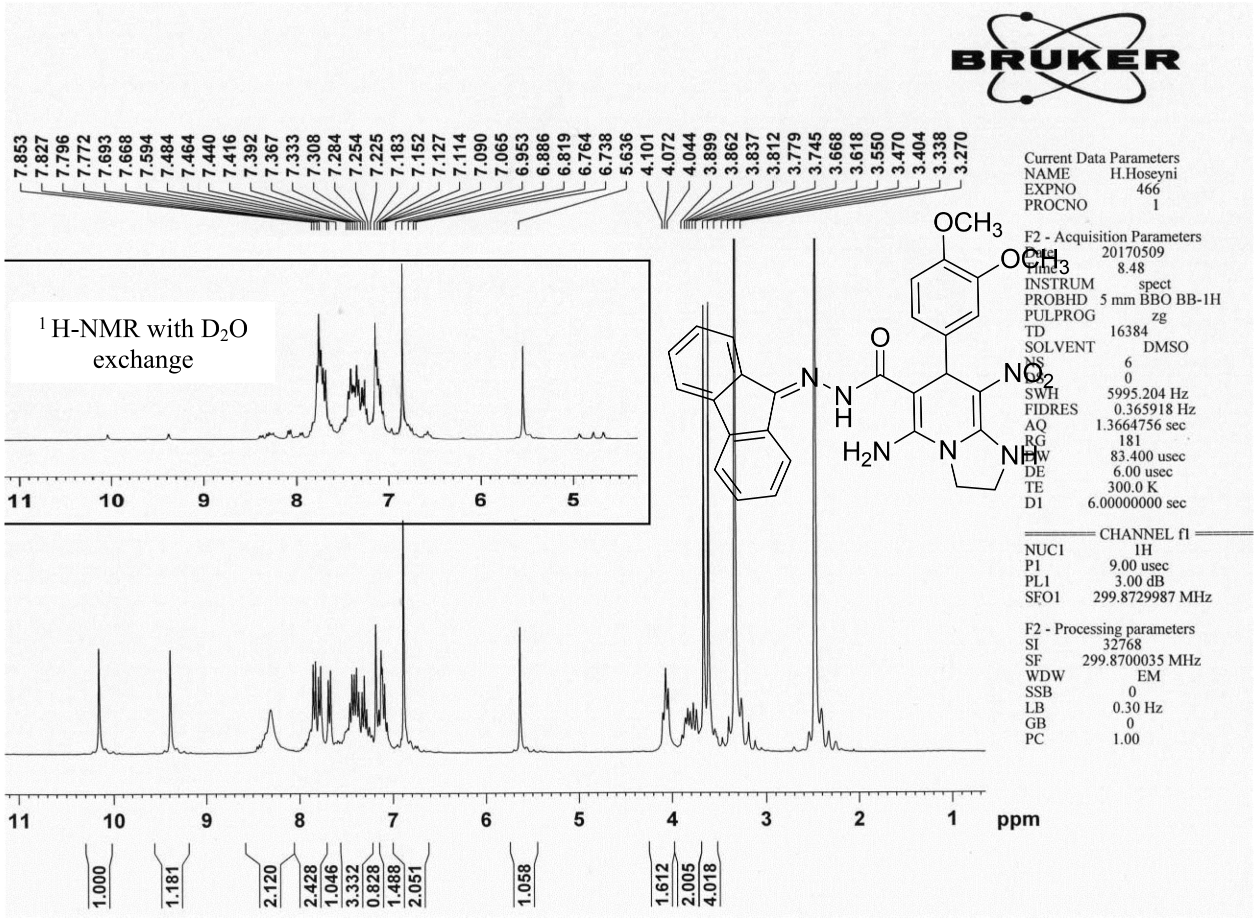
BRUKER
[1] H-NMR with D2O exchange
ppm
Current Data Parameters
NAME H.Hoseyni
EXPNO 466
PROCNO 1
F2 - Acquisition Parameters
INSTRUM spect
PROBHD 5 mm BBO BB-1H
PULPROG zg
TD 16384
SOLVENT DMSO
SWH 5995.204 Hz
FIDRES 0.365918 Hz
AQ 1.3664756 sec
DE 6.00 usec
TE 300.0 K
D1 6.00000000 sec
CHANNEL f1
NUC1 1H
P1 9.00 usec
PL1 3.00 dB
SFO1 299.8729987 MHz
F2 - Processing parameters
SI 32768
SF 299.8700035 MHz
WDW EM
SSB 0
LB 0.30 Hz
GB 0
PC 1.00

¹H NMR of 6g

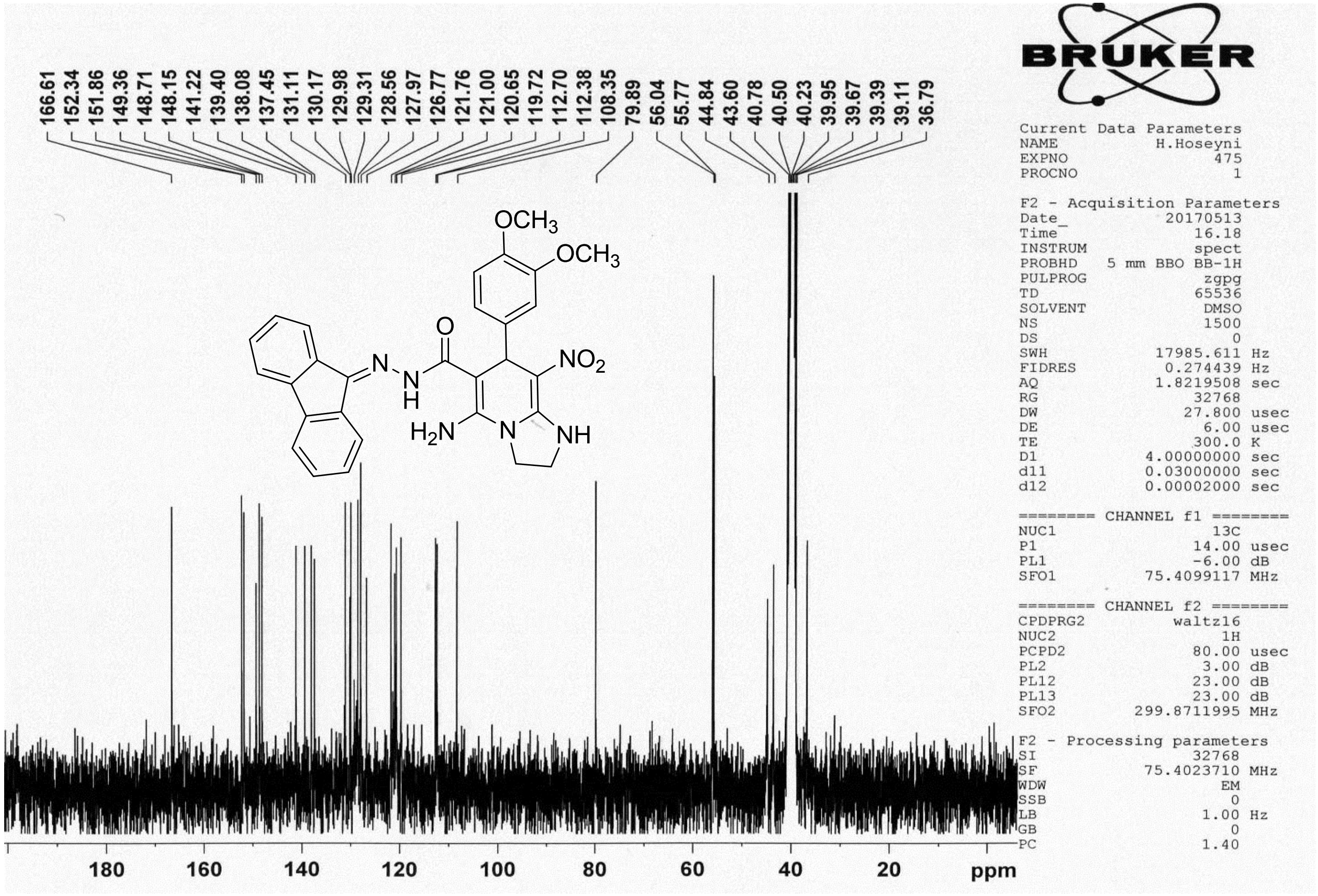

^{13}C NMR of 6g

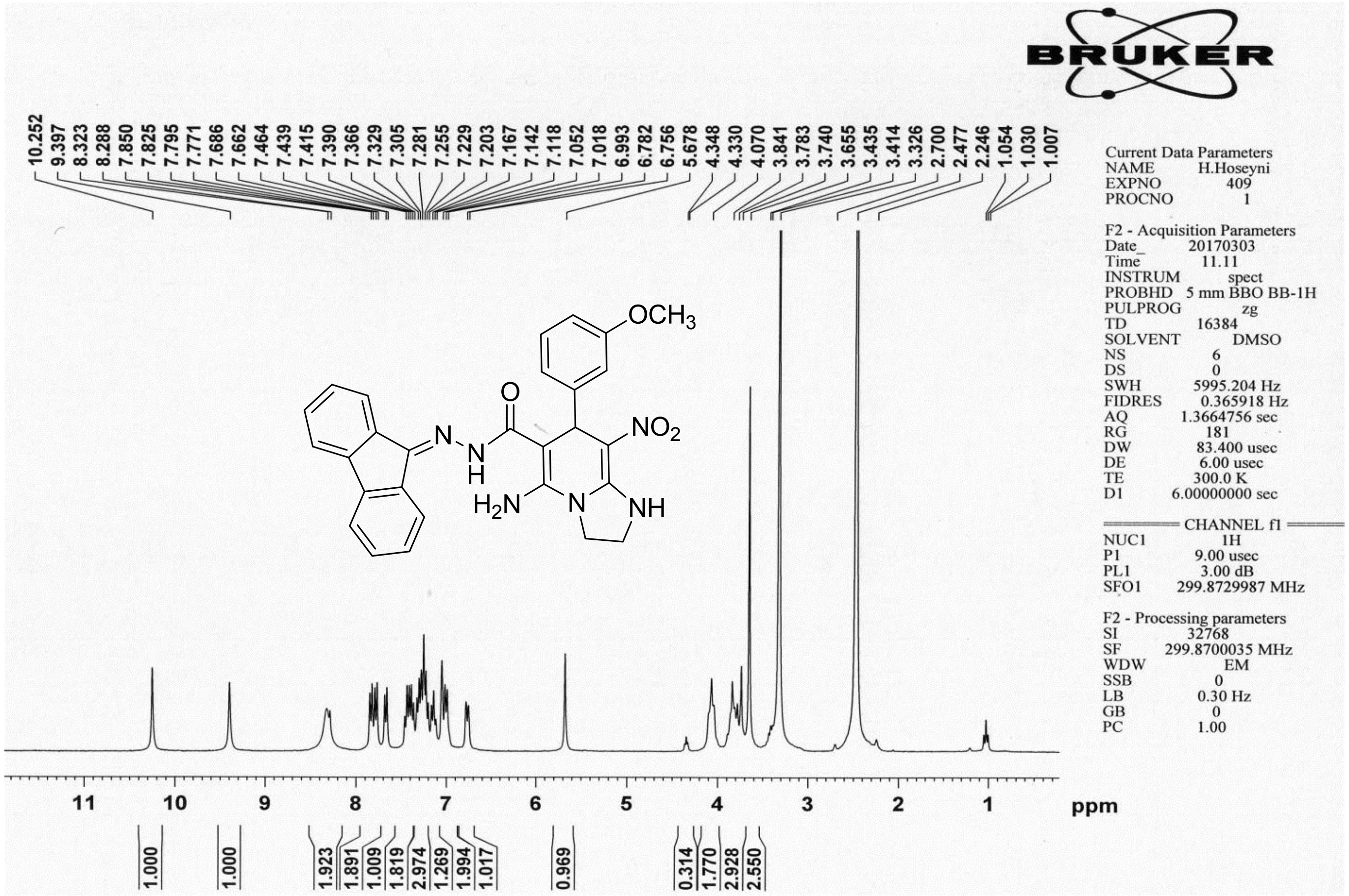
BRUKER
10.252
9.397
8.323
8.288
7.850
7.825
7.795
7.771
7.686
7.662
7.464
7.439
7.415
7.390
7.366
7.329
7.305
7.281
7.255
7.229
7.203
7.167
7.142
7.118
7.052
7.018
6.993
6.782
6.756
5.678
4.348
4.330
4.070
3.841
3.783
3.740
3.655
3.435
3.414
3.326
2.700
2.477
2.246
1.054
1.030
1.007
Current Data Parameters
NAME H.Hoseyni
EXPNO 409
PROCNO 1
F2 - Acquisition Parameters
Date_ 20170303
Time 11.11
INSTRUM spect
PROBHD 5 mm BBO BB-1H
PULPROG zg
TD 16384
SOLVENT DMSO
NS 6
DS 0
SWH 5995.204 Hz
FIDRES 0.365918 Hz
AQ 1.3664756 sec
RG 181
DW 83.400 usec
DE 6.00 usec
TE 300.0 K
D1 6.00000000 sec
CHANNEL f1
NUC1 1H
P1 9.00 usec
PL1 3.00 dB
SFO1 299.8729987 MHz
F2 - Processing parameters
SI 32768
SF 299.8700035 MHz
WDW EM
SSB 0
LB 0.30 Hz
GB 0
PC 1.00
OCH3
O
NO2
N
N
H
H2N
N
NH
11
10
9
8
7
6
5
4
3
2
1
ppm
1.000
1.000
1.923
1.891
1.009
1.819
2.974
1.269
1.994
1.017
0.969
0.314
1.770
2.928
2.550

^{1}H NMR of 6h

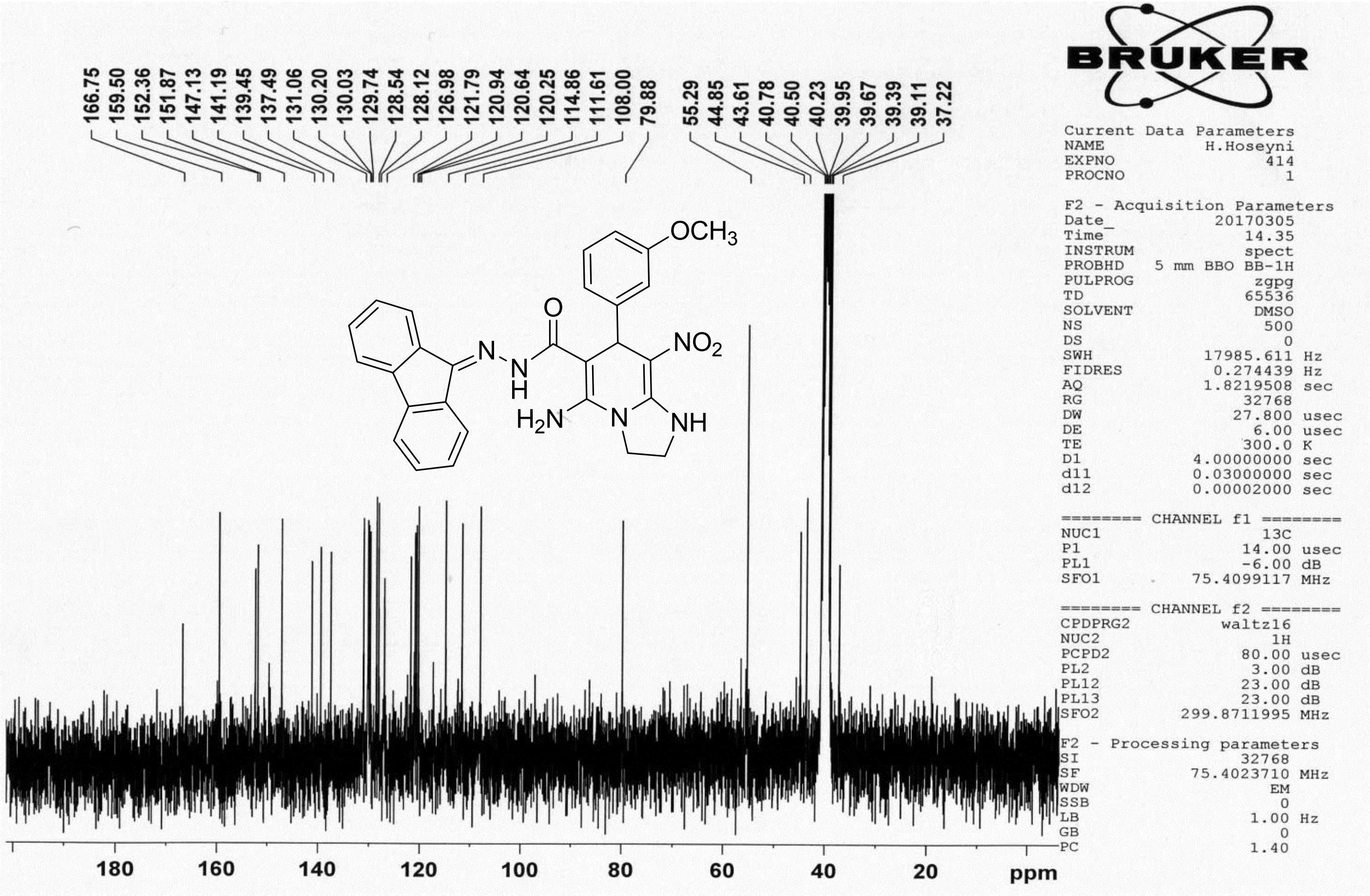

^{13}C NMR of 6h

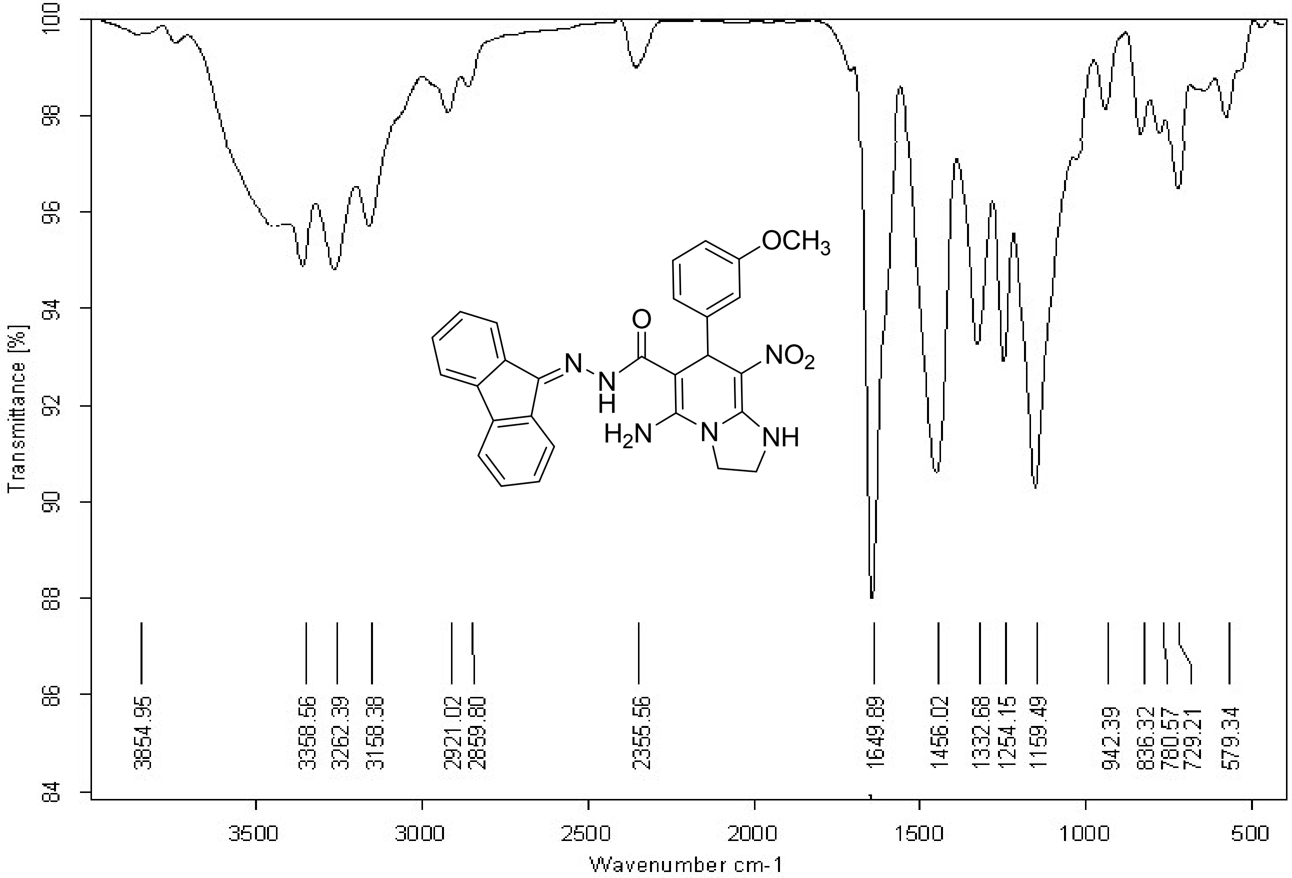
Transmittance [%]
100
98
96
94
92
90
88
86
84
3854.95
3358.56
3262.39
3158.38
2921.02
2859.80
2355.56
1649.89
1456.02
1332.68
1254.15
1159.49
942.39
836.32
780.57
729.21
579.34
3500
3000
2500
2000
1500
1000
500
Wavenumber cm-1
OCH3
O
NO2
N
N
H
H2N
N
NH

IR of 6h

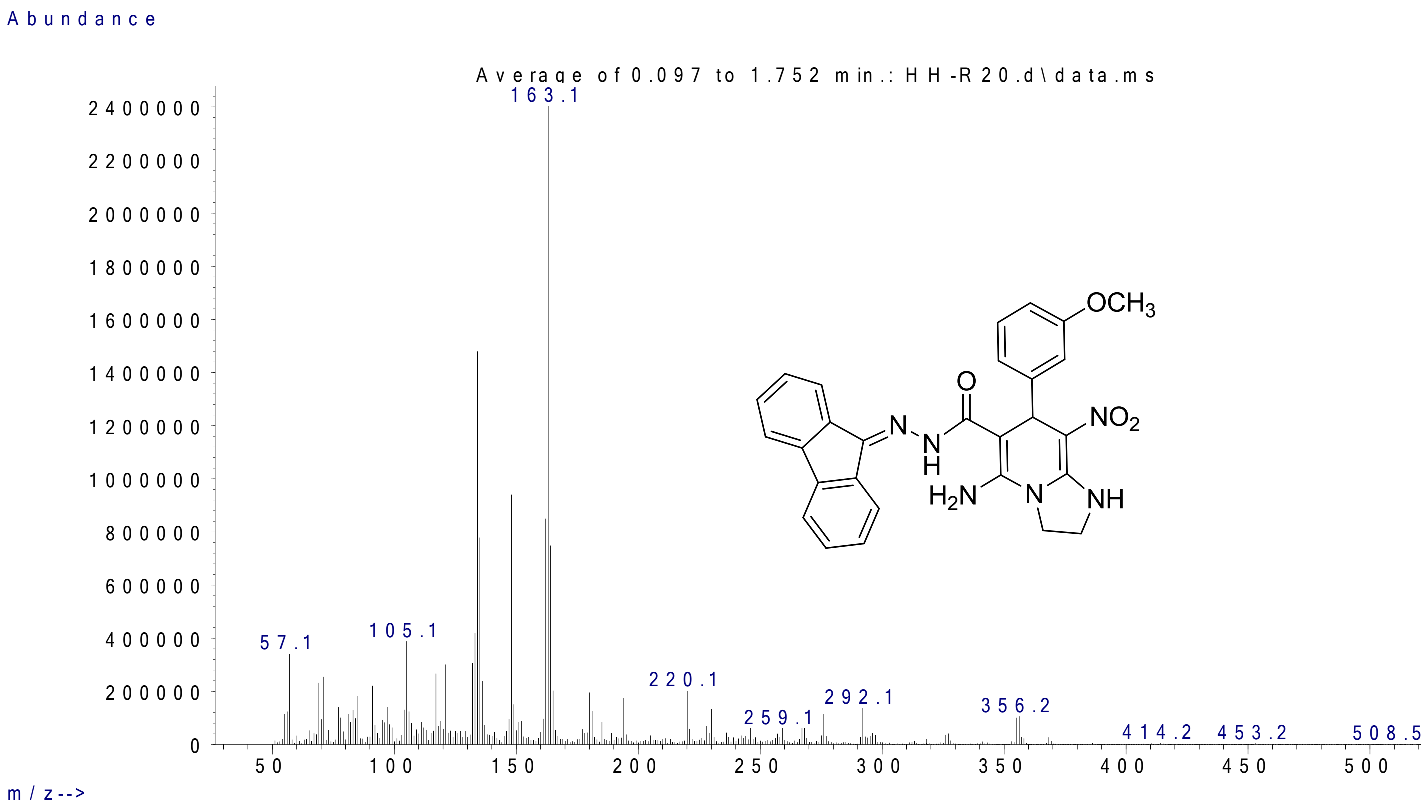
Abundance
Average of 0.097 to 1.752 min.: HH-R20.d\data.ms
163.1
2400000
2200000
2000000
1800000
1600000
1400000
1200000
1000000
800000
600000
400000
200000
0
57.1
105.1
220.1
259.1
292.1
356.2
414.2
453.2
508.5
50
100
150
200
250
300
350
400
450
500
m/z-->
OCH3
O
N
N
H
NO2
H2N
N
NH

MS of 6h

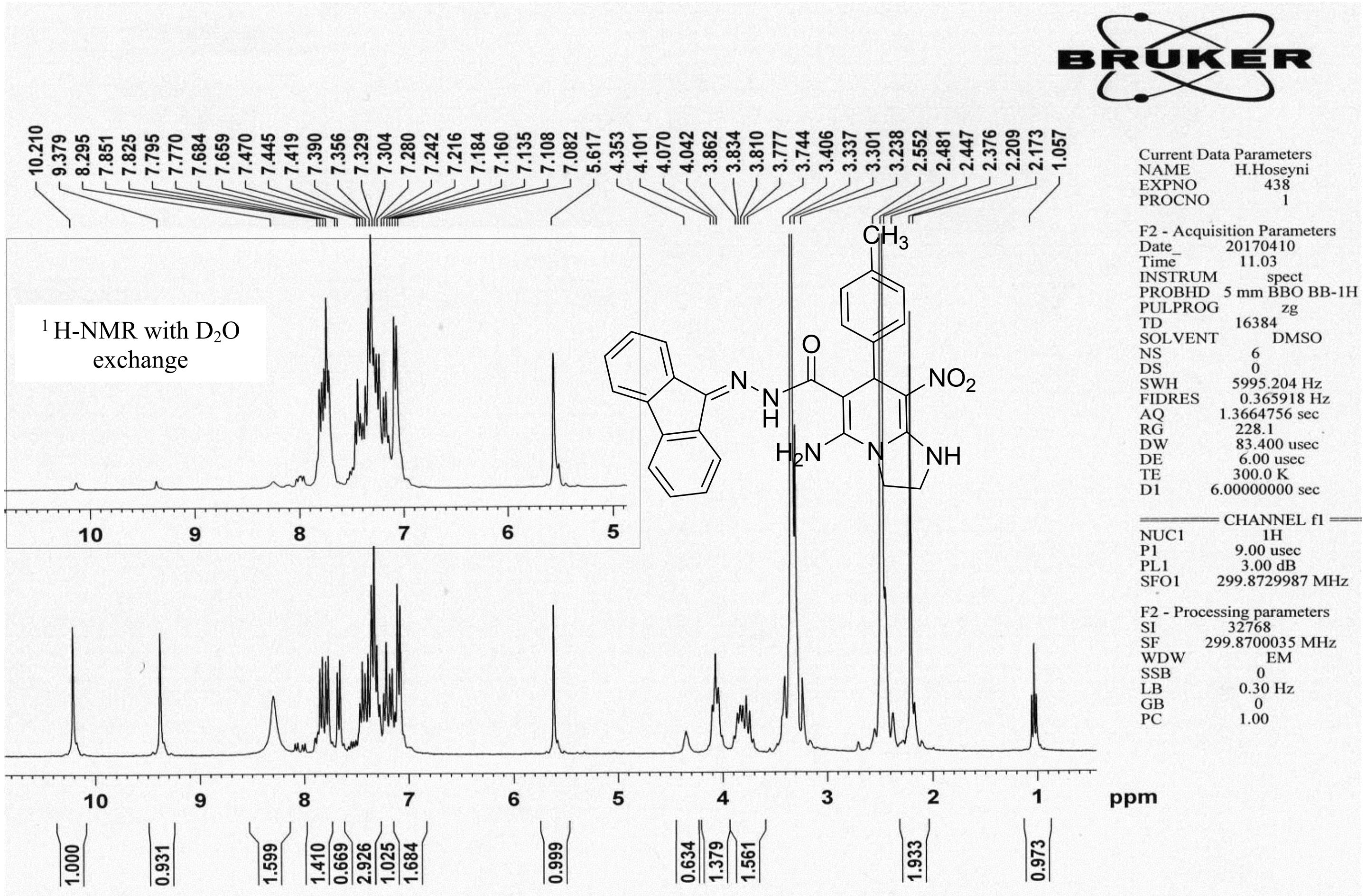
BRUKER
10.210 9.379 8.295 7.851 7.825 7.795 7.770 7.684 7.659 7.470 7.445 7.419 7.390 7.356 7.329 7.304 7.280 7.242 7.216 7.184 7.160 7.135 7.108 7.082 5.617 4.353 4.101 4.070 4.042 3.862 3.834 3.810 3.777 3.744 3.406 3.337 3.301 3.238 2.552 2.481 2.447 2.376 2.209 2.173 1.057
Current Data Parameters
NAME H.Hoseyni
EXPNO 438
PROCNO 1
F2 - Acquisition Parameters
Date_ 20170410
Time 11.03
INSTRUM spect
PROBHD 5 mm BBO BB-1H
PULPROG zg
TD 16384
SOLVENT DMSO
NS 6
DS 0
SWH 5995.204 Hz
FIDRES 0.365918 Hz
AQ 1.3664756 sec
RG 228.1
DW 83.400 usec
DE 6.00 usec
TE 300.0 K
D1 6.00000000 sec
CHANNEL f1
NUC1 1H
P1 9.00 usec
PL1 3.00 dB
SFO1 299.8729987 MHz
F2 - Processing parameters
SI 32768
SF 299.8700035 MHz
WDW EM
SSB 0
LB 0.30 Hz
GB 0
PC 1.00
1 H-NMR with D2O exchange
CH3
O
N
N
H
NO2
H2N
N
NH
10 9 8 7 6 5 4 3 2 1 ppm
1.000 0.931 1.599 1.410 0.669 2.926 1.025 1.684 0.999 0.634 1.379 1.561 1.933 0.973

^{1}H NMR of 6i

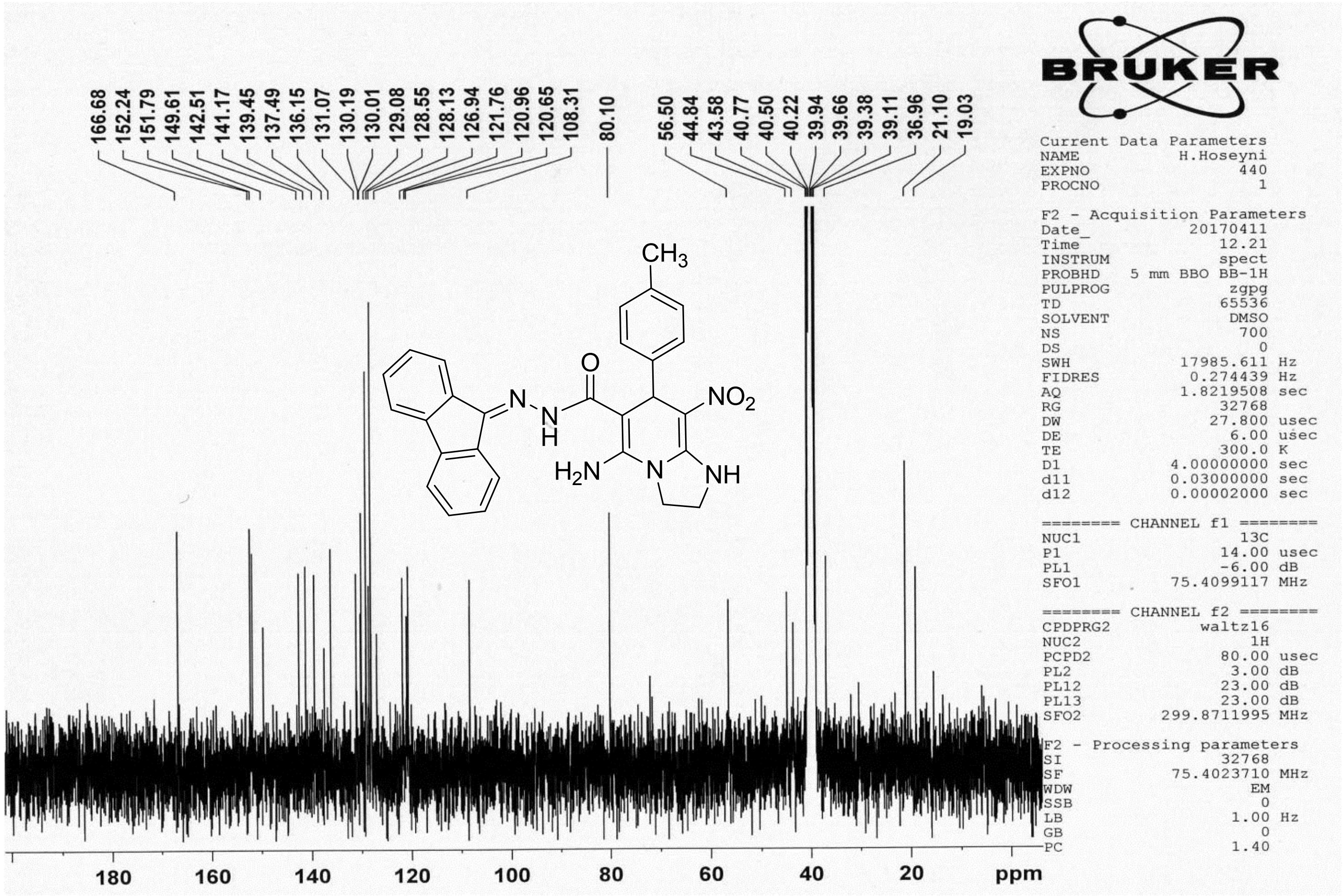
166.68
152.24
151.79
149.61
142.51
141.17
139.45
137.49
136.15
131.07
130.19
130.01
129.08
128.55
128.13
126.94
121.76
120.96
120.65
108.31
80.10
56.50
44.84
43.58
40.77
40.50
40.22
39.94
39.66
39.38
39.11
36.96
21.10
19.03
BRUKER
Current Data Parameters
NAME H.Hoseyni
EXPNO 440
PROCNO 1
F2 - Acquisition Parameters
Date_ 20170411
Time 12.21
INSTRUM spect
PROBHD 5 mm BBO BB-1H
PULPROG zgpg
TD 65536
SOLVENT DMSO
NS 700
DS 0
SWH 17985.611 Hz
FIDRES 0.274439 Hz
AQ 1.8219508 sec
RG 32768
DW 27.800 usec
DE 6.00 usec
TE 300.0 K
D1 4.00000000 sec
d11 0.03000000 sec
d12 0.00002000 sec
======== CHANNEL f1 ========
NUC1 13C
P1 14.00 usec
PL1 -6.00 dB
SFO1 75.4099117 MHz
======== CHANNEL f2 ========
CPDPRG2 waltz16
NUC2 1H
PCPD2 80.00 usec
PL2 3.00 dB
PL12 23.00 dB
PL13 23.00 dB
SFO2 299.8711995 MHz
F2 - Processing parameters
SI 32768
SF 75.4023710 MHz
WDW EM
SSB 0
LB 1.00 Hz
GB 0
PC 1.40
CH3
O
N
N
H
NO2
H2N
N
NH
180
160
140
120
100
80
60
40
20
ppm

^{13}C NMR of 6i

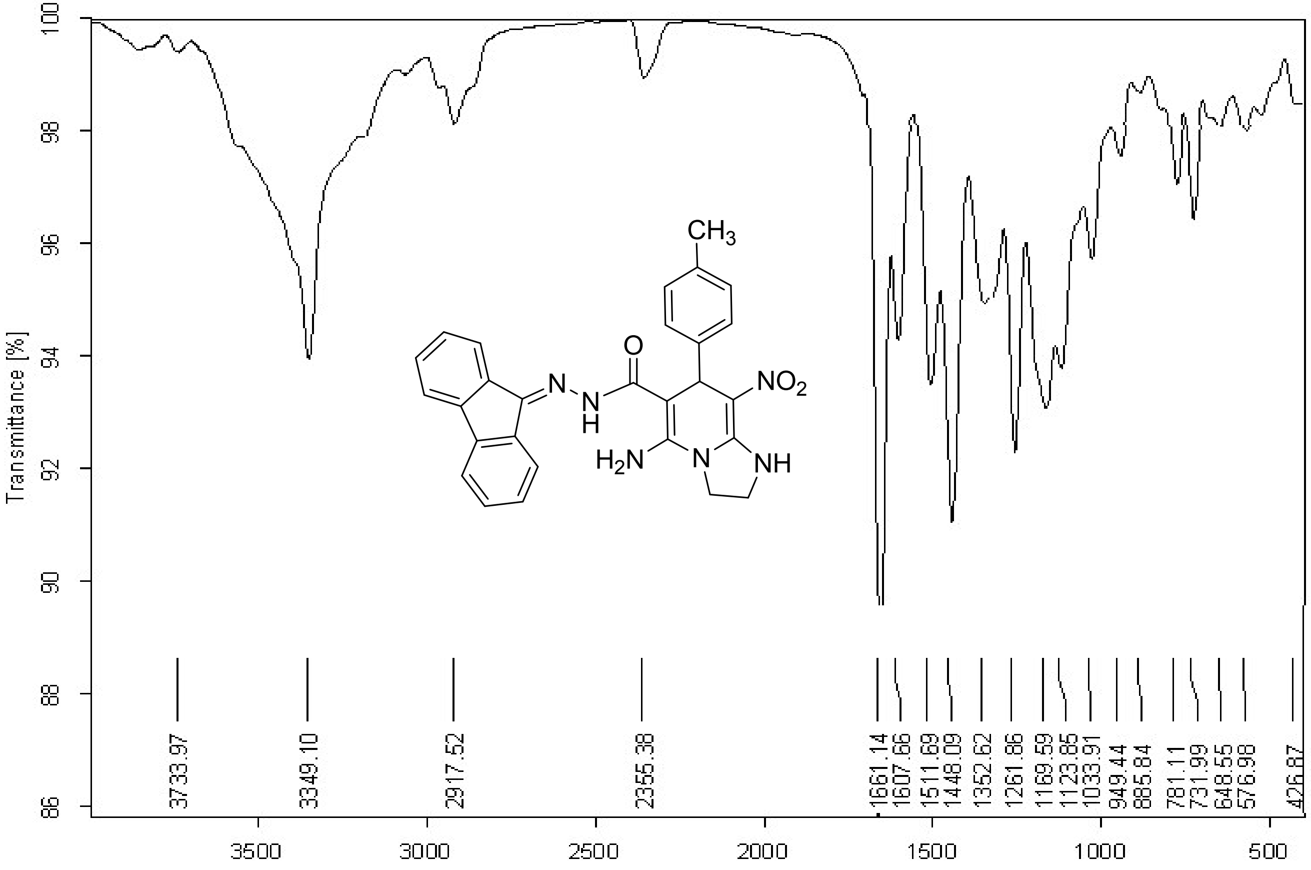
Transmittance [%]
100
98
96
94
92
90
88
86
3500
3000
2500
2000
1500
1000
500
3733.97
3349.10
2917.52
2355.38
1661.14
1607.66
1511.69
1448.09
1352.62
1261.86
1169.59
1123.85
1033.91
949.44
885.84
781.11
731.99
648.55
576.98
426.87
CH3
O
N
N
H
NO2
H2N
N
NH

IR of 6i

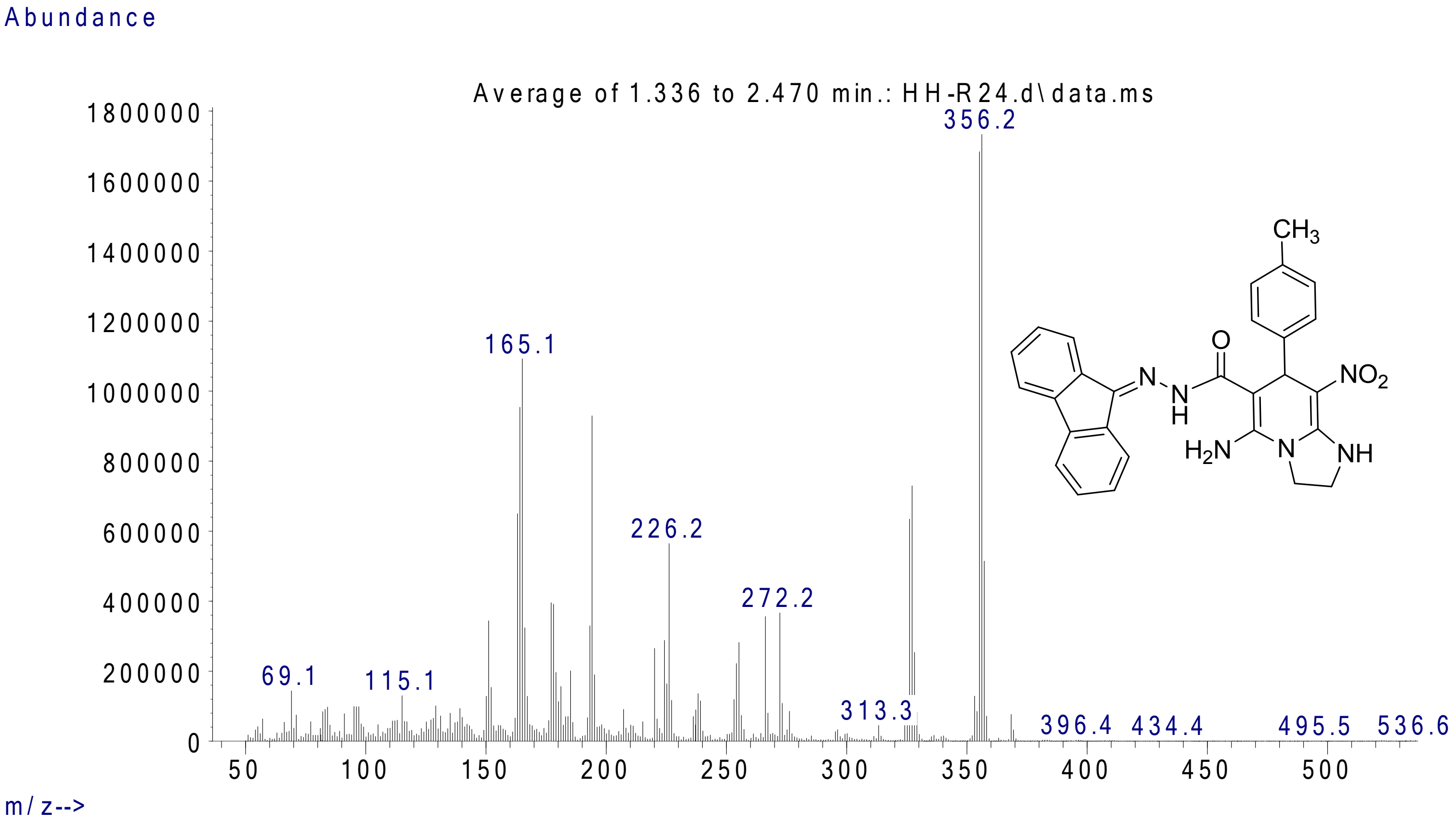
Abundance
Average of 1.336 to 2.470 min.: HH-R24.d\data.ms
1800000
1600000
1400000
1200000
1000000
800000
600000
400000
200000
0
356.2
165.1
226.2
272.2
69.1
115.1
313.3
396.4
434.4
495.5
536.6
50
100
150
200
250
300
350
400
450
500
m/z-->
CH3
O
N
N
H
NO2
H2N
N
NH

MS of 6i

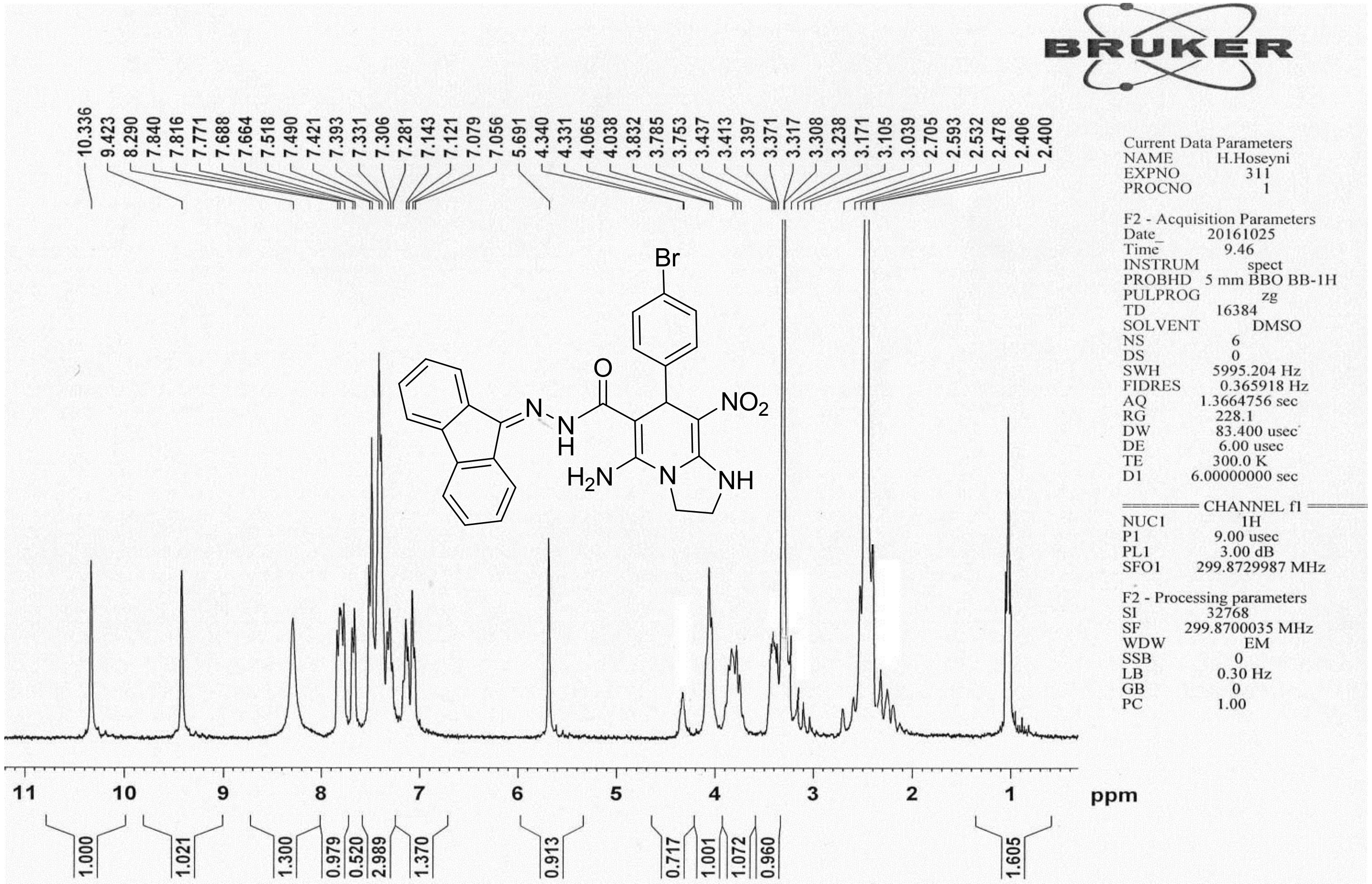
BRUKER
10.336
9.423
8.290
7.840
7.816
7.771
7.688
7.664
7.518
7.490
7.421
7.393
7.331
7.306
7.281
7.143
7.121
7.079
7.056
5.691
4.340
4.331
4.065
4.038
3.832
3.785
3.753
3.437
3.413
3.397
3.371
3.317
3.308
3.238
3.171
3.105
3.039
2.705
2.593
2.532
2.478
2.406
2.400
Current Data Parameters
NAME H.Hoseyni
EXPNO 311
PROCNO 1
F2 - Acquisition Parameters
Date_ 20161025
Time 9.46
INSTRUM spect
PROBHD 5 mm BBO BB-1H
PULPROG zg
TD 16384
SOLVENT DMSO
NS 6
DS 0
SWH 5995.204 Hz
FIDRES 0.365918 Hz
AQ 1.3664756 sec
RG 228.1
DW 83.400 usec
DE 6.00 usec
TE 300.0 K
D1 6.00000000 sec
CHANNEL f1
NUC1 1H
P1 9.00 usec
PL1 3.00 dB
SFO1 299.8729987 MHz
F2 - Processing parameters
SI 32768
SF 299.8700035 MHz
WDW EM
SSB 0
LB 0.30 Hz
GB 0
PC 1.00
Br
O
N
N
H
NO2
H2N
N
NH
11 10 9 8 7 6 5 4 3 2 1 ppm
1.000
1.021
1.300
0.979
0.520
2.989
1.370
0.913
0.717
1.001
1.072
0.960
1.605

^{1}H NMR of 6j

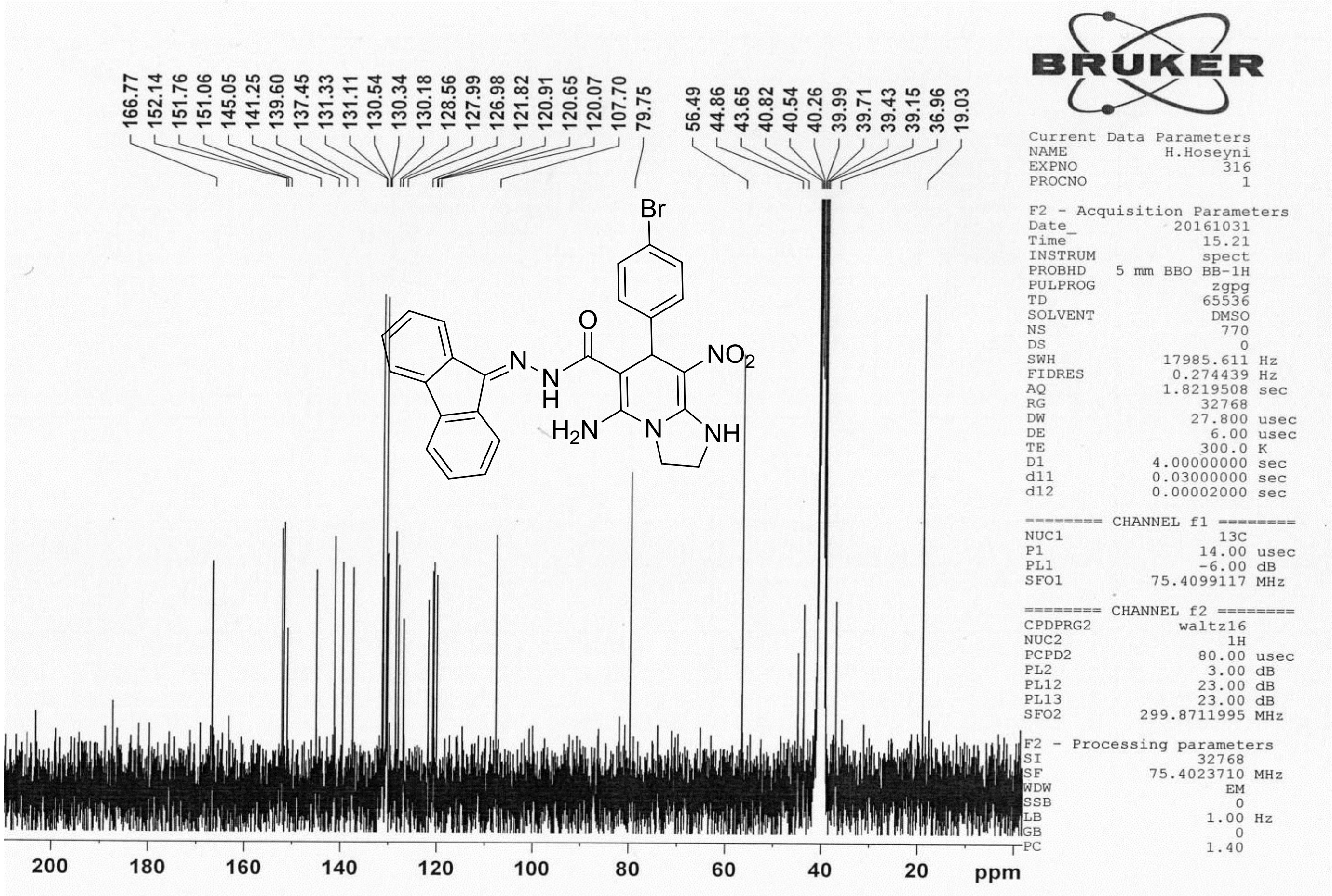

166.77
152.14
151.76
151.06
145.05
141.25
139.60
137.45
131.33
131.11
130.54
130.34
130.18
128.56
127.99
126.98
121.82
120.91
120.65
120.07
107.70
79.75
56.49
44.86
43.65
40.82
40.54
40.26
39.99
39.71
39.43
39.15
36.96
19.03
BRUKER
Current Data Parameters
NAME H.Hoseyni
EXPNO 316
PROCNO 1
F2 - Acquisition Parameters
Date_ 20161031
Time 15.21
INSTRUM spect
PROBHD 5 mm BBO BB-1H
PULPROG zgpg
TD 65536
SOLVENT DMSO
NS 770
DS 0
SWH 17985.611 Hz
FIDRES 0.274439 Hz
AQ 1.8219508 sec
RG 32768
DW 27.800 usec
DE 6.00 usec
TE 300.0 K
D1 4.00000000 sec
d11 0.03000000 sec
d12 0.00002000 sec
======== CHANNEL f1 ========
NUC1 13C
P1 14.00 usec
PL1 -6.00 dB
SFO1 75.4099117 MHz
======== CHANNEL f2 ========
CPDPRG2 waltz16
NUC2 1H
PCPD2 80.00 usec
PL2 3.00 dB
PL12 23.00 dB
PL13 23.00 dB
SFO2 299.8711995 MHz
F2 - Processing parameters
SI 32768
SF 75.4023710 MHz
WDW EM
SSB 0
LB 1.00 Hz
GB 0
PC 1.40
Br
O
N
N
H
NO2
H2N
N
NH
200
180
160
140
120
100
80
60
40
20
ppm

^{13}C NMR of 6j

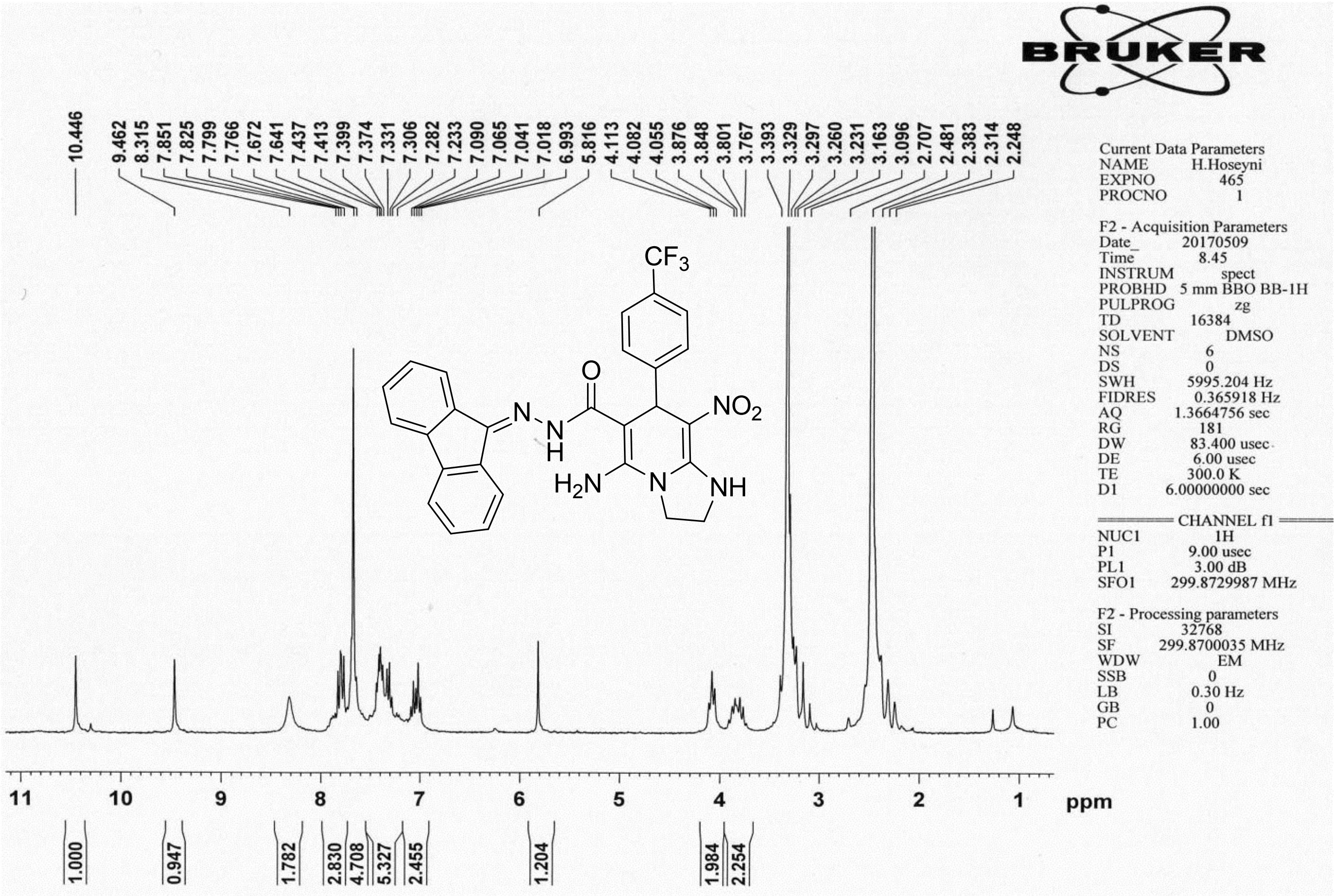
BRUKER
10.446
9.462
8.315
7.851
7.825
7.799
7.766
7.672
7.641
7.437
7.413
7.399
7.374
7.331
7.306
7.282
7.233
7.090
7.065
7.041
7.018
6.993
5.816
4.113
4.082
4.055
3.876
3.848
3.801
3.767
3.393
3.329
3.297
3.260
3.231
3.163
3.096
2.707
2.481
2.383
2.314
2.248
CF3
O
N
N
H
NO2
H2N
N
NH
Current Data Parameters
NAME H.Hoseyni
EXPNO 465
PROCNO 1
F2 - Acquisition Parameters
Date_ 20170509
Time 8.45
INSTRUM spect
PROBHD 5 mm BBO BB-1H
PULPROG zg
TD 16384
SOLVENT DMSO
NS 6
DS 0
SWH 5995.204 Hz
FIDRES 0.365918 Hz
AQ 1.3664756 sec
RG 181
DW 83.400 usec
DE 6.00 usec
TE 300.0 K
D1 6.00000000 sec
CHANNEL f1
NUC1 1H
P1 9.00 usec
PL1 3.00 dB
SFO1 299.8729987 MHz
F2 - Processing parameters
SI 32768
SF 299.8700035 MHz
WDW EM
SSB 0
LB 0.30 Hz
GB 0
PC 1.00
11
10
9
8
7
6
5
4
3
2
1
ppm
1.000
0.947
1.782
2.830
4.708
5.327
2.455
1.204
1.984
2.254

^{1}H NMR of 6k

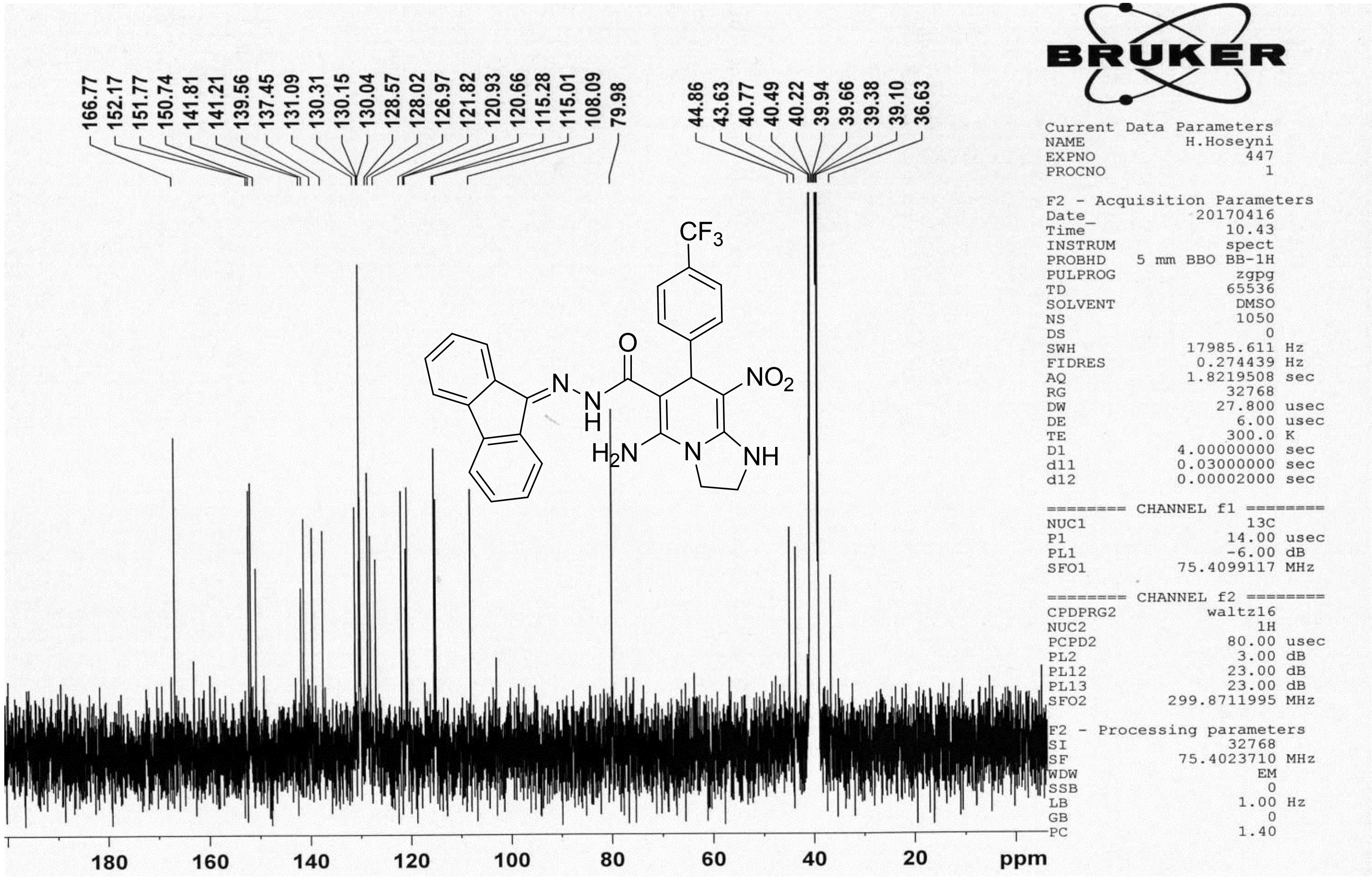

166.77
152.17
151.77
150.74
141.81
141.21
139.56
137.45
131.09
130.31
130.15
130.04
128.57
128.02
126.97
121.82
120.93
120.66
115.28
115.01
108.09
79.98
44.86
43.63
40.77
40.49
40.22
39.94
39.66
39.38
39.10
36.63
BRUKER
Current Data Parameters
NAME H.Hoseyni
EXPNO 447
PROCNO 1
F2 - Acquisition Parameters
Date_ 20170416
Time 10.43
INSTRUM spect
PROBHD 5 mm BBO BB-1H
PULPROG zgpg
TD 65536
SOLVENT DMSO
NS 1050
DS 0
SWH 17985.611 Hz
FIDRES 0.274439 Hz
AQ 1.8219508 sec
RG 32768
DW 27.800 usec
DE 6.00 usec
TE 300.0 K
D1 4.00000000 sec
d11 0.03000000 sec
d12 0.00002000 sec
======== CHANNEL f1 ========
NUC1 13C
P1 14.00 usec
PL1 -6.00 dB
SFO1 75.4099117 MHz
======== CHANNEL f2 ========
CPDPRG2 waltz16
NUC2 1H
PCPD2 80.00 usec
PL2 3.00 dB
PL12 23.00 dB
PL13 23.00 dB
SFO2 299.8711995 MHz
F2 - Processing parameters
SI 32768
SF 75.4023710 MHz
WDW EM
SSB 0
LB 1.00 Hz
GB 0
PC 1.40
CF3
O
N
N
H
NO2
H2N
N
NH
180
160
140
120
100
80
60
40
20
ppm

^{13}C NMR of 6k
